# Isoxazolium N-ylides and 1-oxa-5-azahexa-1,3,5-trienes on the way from isoxazoles to 2*H*-1,3-oxazines

**DOI:** 10.3762/bjoc.10.197

**Published:** 2014-08-14

**Authors:** Alexander F Khlebnikov, Mikhail S Novikov, Yelizaveta G Gorbunova, Ekaterina E Galenko, Kirill I Mikhailov, Viktoriia V Pakalnis, Margarita S Avdontceva

**Affiliations:** 1 Institute of Chemistry, Saint-Petersburg State University, Universitetskii pr. 26, 198504 St. Petersburg, Russia

**Keywords:** diazo esters, isoxazoles, isoxazolium N-ylides, 2-azabuta-1,3-dienes, 2*H*-1,3-oxazines

## Abstract

Theoretical and experimental studies of the reaction of isoxazoles with diazo compounds show that the formation of 2*H*-1,3-oxazines proceeds via the formation of (3*Z*)-1-oxa-5-azahexa-1,3,5-trienes which undergo a 6π-cyclization. The stationary points corresponding to the probable reaction intermediates, isoxazolium N-ylides, were located by DFT calculations at the B3LYP/6-31G(d) level only for derivatives without a substituent in position 3 of the isoxazole ring. These isoxazolium *N*-ylides are thermodynamically and kinetically very unstable. According to the calculations and experimental results 2*H*-1,3-oxazines are usually more thermodynamically stable than the corresponding open-chain isomers, (3*Z*)-1-oxa-5-azahexa-1,3,5-trienes. The exception are oxaazahexatrienes derived from 5-alkoxyisoxazoles, which are thermodynamically more stable than the corresponding 2*H*-1,3-oxazines. Therefore, the reaction of diazo esters with 5-alkoxyisoxazoles is a good approach to 1,4-di(alkoxycarbonyl)-2-azabuta-1,3-dienes. The reaction conditions for the preparation of aryl- and halogen-substituted 2*H*-1,3-oxazines and 1,4-di(alkoxycarbonyl)-2-azabuta-1,3-dienes from isoxazoles were investigated.

## Introduction

Isoxazoles are versatile building blocks, which have found extensive use in organic synthesis [1–3]. However, reactions of isoxazoles with diazo compounds have scarcely been studied [1–5]. In 2008 Davies and Manning [4–5] discovered the Rh-catalyzed reaction of diazo esters with 3,5-dialkylisoxazoles, benzo[*d*]isoxazole and 3-chlorobenzo[*d*]isothiazole leading to the corresponding 2*H*-1,3-oxazines, 2*H*-benzo[*e*][1,3]oxazine and 2*H*-benzo[*e*][1,3]thiazine.

**Scheme 1:**
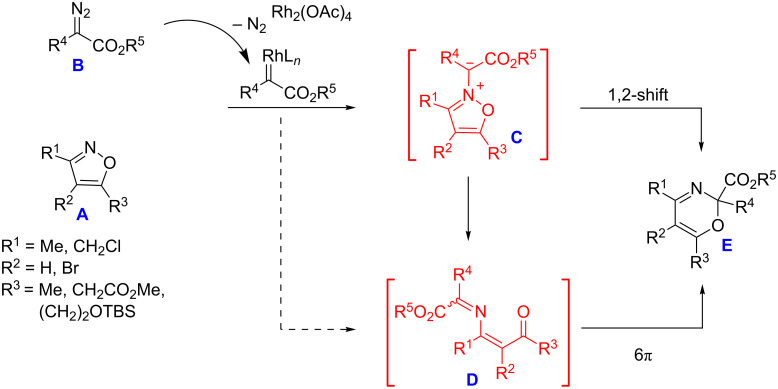
Mechanistic scheme of the formation of 2*H*-1,3-oxazine by the reaction of isoxazoles with a diazo compound.

The authors assumed that the reaction of isoxazoles **A** with diazo esters **B** involved an isoxazolium N-ylide intermediate **C** formed by an attack of the rhodium carbenoid onto the isoxazole nitrogen. Furthermore, ylide **C** could undergo either a 1,2-shift to directly generate oxazine **E** or a ring opening to 1-oxa-5-azahexa-1,3,5-triene **D**, followed by a 6π-electrocyclization to give oxazine **E** (Scheme 1). At the same time, the third mechanism of the reaction, involving a one-step formation of 1-oxa-5-azahexa-1,3,5-triene **D**, cannot be excluded.

The reaction of a carbenoid with isoxazoles is the only known one-step intermolecular reaction which can in principle produce isoxazolium N-ylides from N-unsubstituted isoxazole derivatives. The formation of such ylides as reactive intermediates in the reactions of bases on isoxazolium salts was earlier supposed [6–9]. However, the detection of isoxazolium N-ylides has never been reported.

**Scheme 2:**
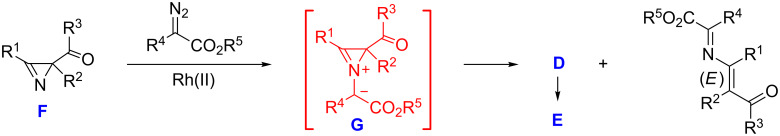
Mechanistic scheme of the formation of 2*H*-1,3-oxazine by the reaction of azirine with a diazo compound.

Recently, we found an alternative synthetic approach to derivatives of 2*H*-1,3-oxazines via a Rh_2_(OAc)_4_-catalyzed reaction of diazo esters with 2-acyl-2*H*-azirines **F**. This reaction involves the intermediate formation of azirinium ylides **G**, their transformation into 1-oxa-5-azahexa-1,3,5-triene **D**, and finally the 6π-electrocyclization of the latter to give oxazine **E** (Scheme 2) [10–11].

Azirinium ylides **G**, formed by the reaction of azirines **F** with carbenoids, can transform into (3*Z*)- and (3*E*)-1-oxa-5-azahexa-1,3,5-triene **D**, but only the former can cyclize into 1,3-oxazines **E**. In contrast, the reaction of isoxazoles with carbenoids results in the exclusive formation of (3*Z*)-1-oxa-5-azahexa-1,3,5-trienes due to geometrical reasons.

The first aim we set ourselves in the present work was to gain insight into the mechanism of the reaction of isoxazoles with diazo compounds by answering the two following questions: “Are isoxazolium N-ylides really formed in this reaction?” and “What is their reactivity?” To this end, we carried out quantum-chemical calculations of the formation and the ring opening of isoxazolium N-ylides. Probing the type of the mechanistic scheme of the 2*H*-1,3-oxazine formation was also conducted by searching for the isoxazoles capable of providing stable 1-oxa-5-azahexa-1,3,5-trienes **D** under reaction with diazo esters and by comparing the experimental results of the reactions of carbenoids with a complementary pair of isoxazole and azirine.

An analysis of recent literature shows that 2*H*-1,3-oxazine derivatives exhibit various types of bioactivity, e.g., herbicidal [12], inhibition of cell growth and enzyme activity [13–18], inhibition of voltage-gated sodium channels [19] and metabotropic glutamate receptor-5a (hmGluR5a) [20]. Consequently, our second aim was to extend the reaction to the preparation of aryl- and halogen-substituted 1,3-oxazines, taking into account that the latter are potential candidates for metal-catalyzed couplings and thus allow further modifications.

## Results and Discussion

**Figure 1:**
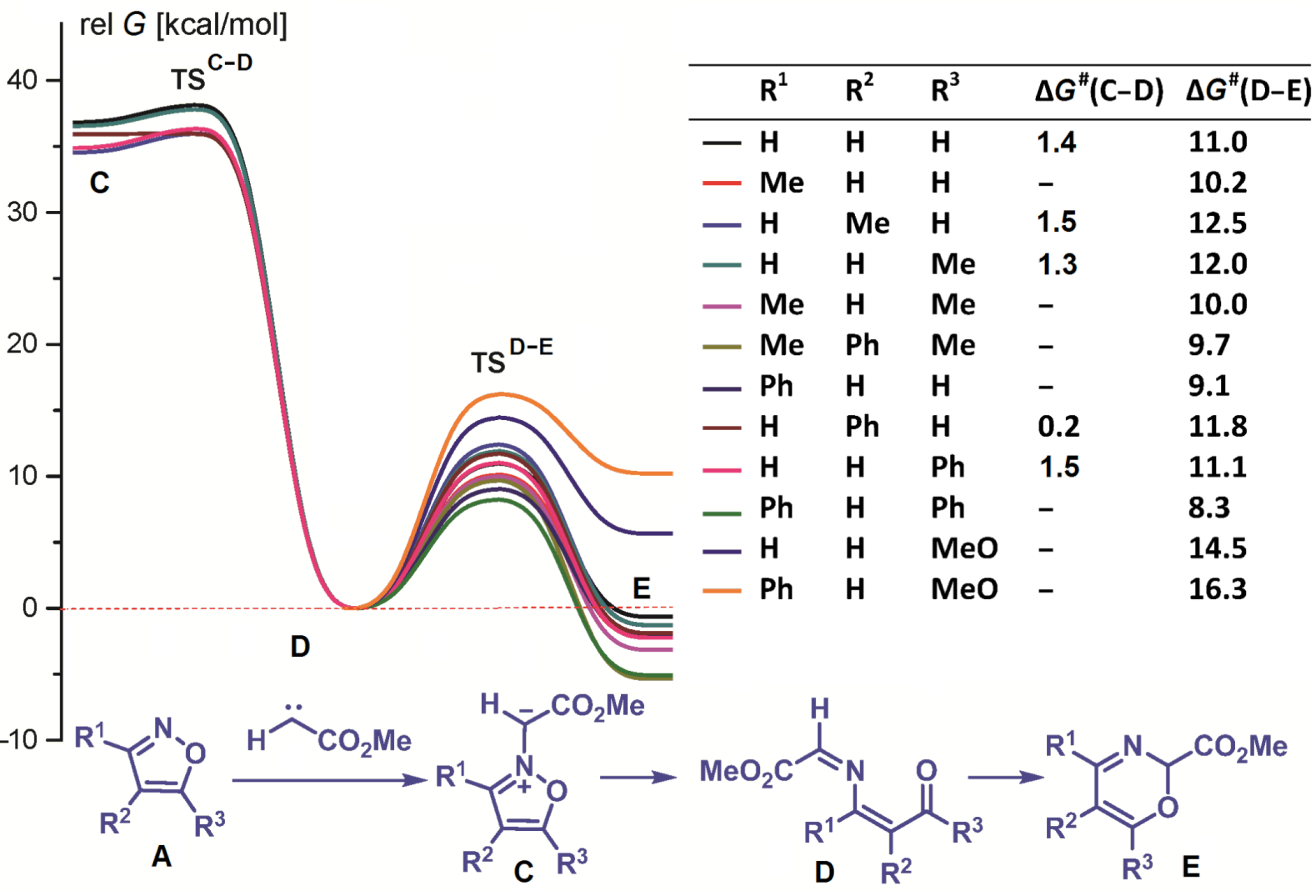
Energy profiles for the transformations of ylides **C**, (3*Z*)-1-oxa-5-azahexa-1,3,5-triene **D** and oxazines **E** derived from methoxycarbonylcarbene and isoxazole **A**. Relative free energies [kcal/mol, 298 K, CH_2_Cl_2_ (PCM)] computed at DFT B3LYP/6-31G(d) level.

The theoretical study of the reaction mechanism was started with an evaluation of the thermodynamic and kinetic stabilities of isoxazolium N-ylides, probable intermediates in a carbenoid- or carbene-mediated one-atom isoxazole ring expansion. Preliminary calculations at the DFT B3LYP/6-31G(d) level with the PCM solvation model for dichloromethane were performed for the model reaction of isoxazoles **A** with methoxycarbonylcarbene (Figure 1). The stationary points corresponding to isoxazolium N-ylides **C** formed by an attack from the methoxycarbonylcarbene on the nitrogen of isoxazole **A** were found only for isoxazoles without substituent R^1^ in position 3.

Further, these ylides undergo a ring opening via very low activation barriers (0.2–1.5 kcal/mol) to give (3*Z*)-1-oxa-5-azahexa-1,3,5-trienes. This is expected, because the oxazole N–O bond is very weak and the reaction is pseudopericyclic [21–22]. The calculated low thermodynamic and kinetic stabilities of the isoxazolium ylides (Figure 1) give only a small chance of detecting their formation even in cases where they can theoretically be formed. If the starting isoxazole contains substituent R^3^, an attack of a carbene on the isoxazole nitrogen leads to (3*Z*)-1-oxa-5-azahexa-1,3,5-triene without an activation barrier. The latter derived from isoxazoles without a methoxy substituent in position 5 can cyclize via a low activation barrier (<12.5 kcal/mol) to the corresponding 2*H*-1,3-oxazines. All calculated 1-oxa-5-azahexa-1,3,5-trienes, excluding the ones derived from 5-methoxy-substituted isoxazoles, are thermodynamically less stable than 2*H*-1,3-oxazines. In contrast, 1,4-di(methoxycarbonyl)-2-azabuta-1,3-dienes are much more stable than the corresponding 1,3-oxazines. We also evaluated the possibility of an attack of methoxycarbonylcarbene on the isoxazole oxygen. According to calculations (see Supporting Information File 1) a carbene attack on the isoxazole oxygen is significantly less favorable than an attack on the nitrogen.

**Figure 2:**
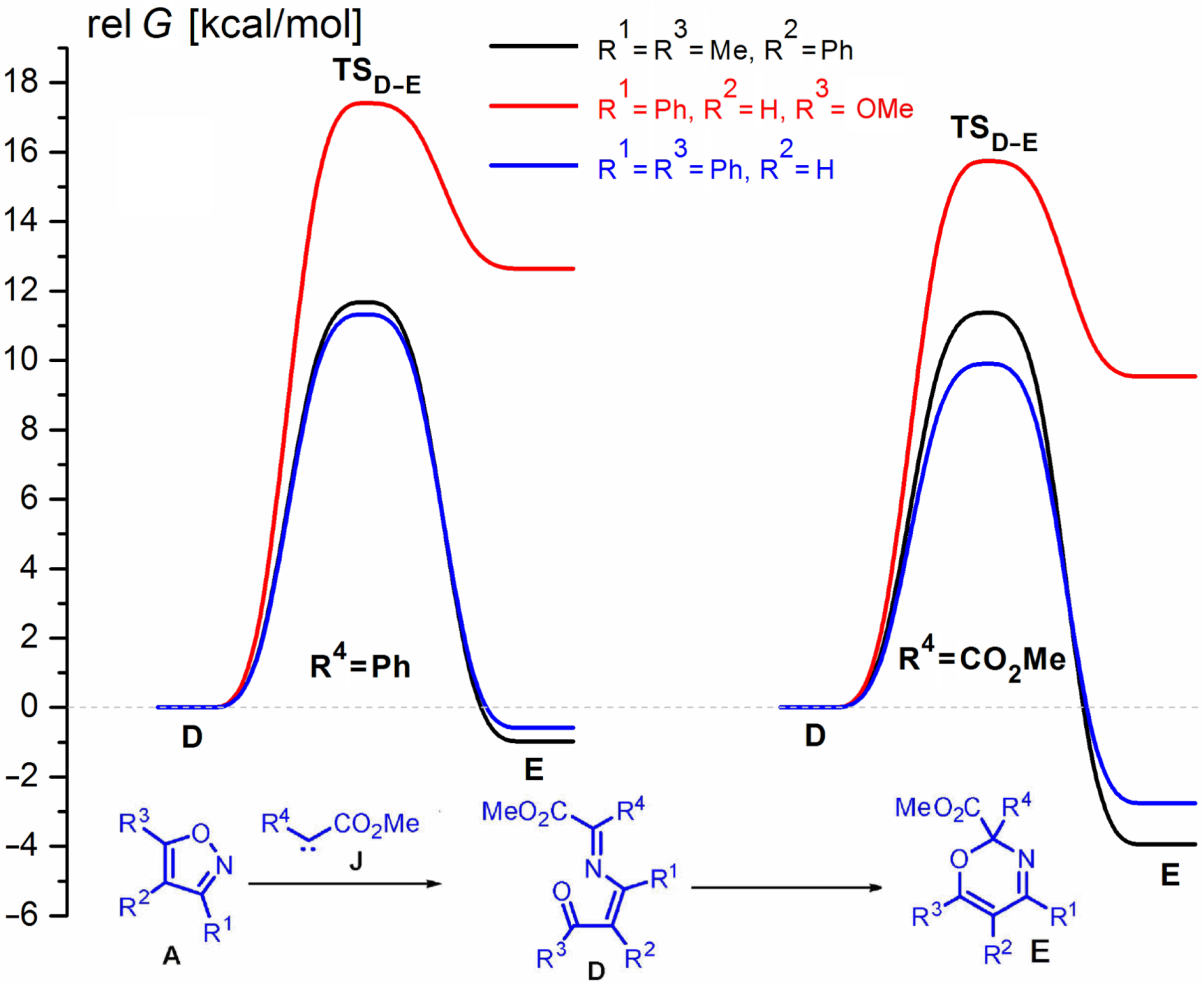
Energy profiles for the transformations of (3*Z*)-1-oxa-5-azahexa-1,3,5-triene **D** and oxazines **E** derived from (methoxycarbonyl)phenylcarbene or di(methoxycarbonyl)carbene and oxazole **A**. Relative free energies [kcal/mol, 298 K, CH_2_Cl_2_ (PCM)] computed at DFT B3LYP/6-31G(d) level.

The results of the calculations do not fundamentally change if methoxycarbonylcarbene is substituted with (methoxycarbonyl)phenylcarbene or di(methoxycarbonyl)carbene (Figure 2). Again, only oxaazahexatrienes **D** derived from 5-methoxy-substituted isoxazoles are much more stable than the corresponding oxazines **E**. Therefore, one can expect the formation of only 1-oxa-5-azahexa-1,3,5-trienes when reacting diazo compounds with 5-methoxyisoxazoles.

**Scheme 3:**
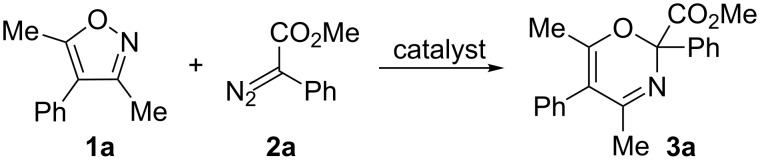
Reaction of isoxazole **1a** and diazo ester **2a**.

To start with, we reacted 4-phenyl-substituted isoxazole **1a** and phenyldiazoacetate **2a** under the reaction conditions used in [4] (catalyst: 1–3 mol % of Rh_2_(OAc)_4_, solvent: CH_2_Cl_2_ or ClCH_2_CH_2_Cl, 40 or 84 °C) (Scheme 3). Unexpectedly, attempts to prepare oxazine **3a** under these conditions were unsuccessful (Scheme 3) and isoxazole **1a** was completely recovered.

Oxazine **3a** was obtained in 14% yield when heated under reflux in CH_2_Cl_2_ and with the use of dirhodium tetraoctanoate instead of Rh_2_(OAc)_4_ as a catalyst. This unsatisfactory result prompted us to test a carbene instead of a Rh(II) carbenoid, since it has been found [23] that carbenes can be successfully generated by thermolysis of diazo compounds without a catalyst in inert solvents with high boiling points, such as trifluoromethylbenzene. These conditions were attempted for the preparation of oxazines from isoxazole **1a** and diazo compounds **2a–c** (Table 1, entries 1–5). The use of a higher boiling-point solvent may also be a means to overcome the low solubility of arylisoxazoles. The formation of an oxazine occurred only with phenyl diazoacetate **2a** under these conditions.

**Table 1:** Reaction of azirines **1a** with diazo compounds **2a–c** without a catalyst.

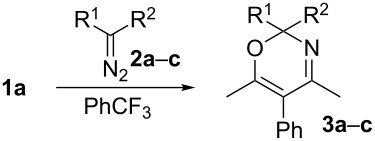					

entry	**2** (R^1^, R^2^)	ratio **1a:2**	time, h	*T*, °C	yield of **3**, %

1	**a** (Ph, CO_2_Me)	5:1	3.5	103	**a**, 34–42^a^
2^b^	**a** (Ph, CO_2_Me)	5:1	3.5	103	**a**, traces
3	**a** (Ph, CO_2_Me)	10:1	3.5	103	**a**, 88^a^
4	**b** (H, CO_2_Et)	5:1	12	103	**b**, traces
5	**c** (CO_2_Me, CO_2_Me)	5:1	38	103	**c**, –
6	**b** (H, CO_2_Et)	3:1	0.3	120, mw	**b**, traces
7	**b** (H, CO_2_Et)	5:1	0.3	120, mw	**b**, traces
8^b^	**c** (CO_2_Me, CO_2_Me)	2:1	0.3	120, mw	**c**, traces

^a^Based on consumed **1a**, the conversion of **1a** was 12–15% (entry 1) and 9% (entry 3); ^b^without solvent.

To overcome the inactivity of diazo compounds **2b**,**c** the use of higher temperature and microwave irradiation were investigated, but only traces of oxazines **3b**,**c** were then detected by ^1^H NMR spectroscopy (Table 1, entries 6–8).

The conditions of choice for the synthesis of aryl-substituted 2*H*-1,3-oxazines proved to be heating under reflux in PhCF_3_ and 1.5–3 mol % of Rh_2_(OAc)_2_ as a catalyst. Under these conditions oxazines **3a–m** were synthesized (Table 2). The yields of oxazines can be improved by using a higher excess of diazo compounds. However, this also leads to an increase of the formation of side products, “carbenoid dimers”, which attribute to a more difficult isolation of the target products in some cases.

**Table 2:** Synthesis of oxazines **3a–m**.

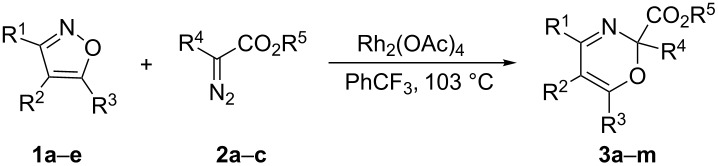								

**1**	**2**	R^1^	R^2^	R^3^	R^4^	R^5^	ratio **1**:**2**	**3**, yield,^a^ %

**a**	**a**	Ме	Ph	Me	Ph	Me	1:1.7 1:3.3	**a**, 26 **a**, 43
**a**	**b**	Ме	Ph	Me	H	Et	1:1.9	**b**, 35
**a**	**c**	Ме	Ph	Me	CO_2_Me	Me	1:1.2	**c**, 67
**b**	**a**	Ph	H	Ph	Ph	Me	1:2.3	**d**, 66 (70)
**b**	**b**	Ph	H	Ph	H	Et	1:3.0	**e**, 34
**b**	**c**	Ph	H	Ph	CO_2_Me	Me	1:3.4	**f**, 81
**c**	**b**	Ph	Cl	Ph	H	Et	1:3.7	**g**, 27 (73)
**c**	**c**	Ph	Cl	Ph	CO_2_Me	Me	1:1.9	**h** , 48
**d**	**b**	Ph	Br	Ph	H	Et	1:3.0	**i**, 19 (75)
**d**	**c**	Ph	Br	Ph	CO_2_Me	Me	1:1.5	**k**, 21
**e**	**b**	Ph	I	Ph	H	Et	1:3.3	**l**, 22 (63)
**e**	**c**	Ph	I	Ph	CO_2_Me	Me	1:1.9	**m**, 21 (36)

^a^Yields based on consumed isoxazole are listed in parentheses.

**Figure 3:**
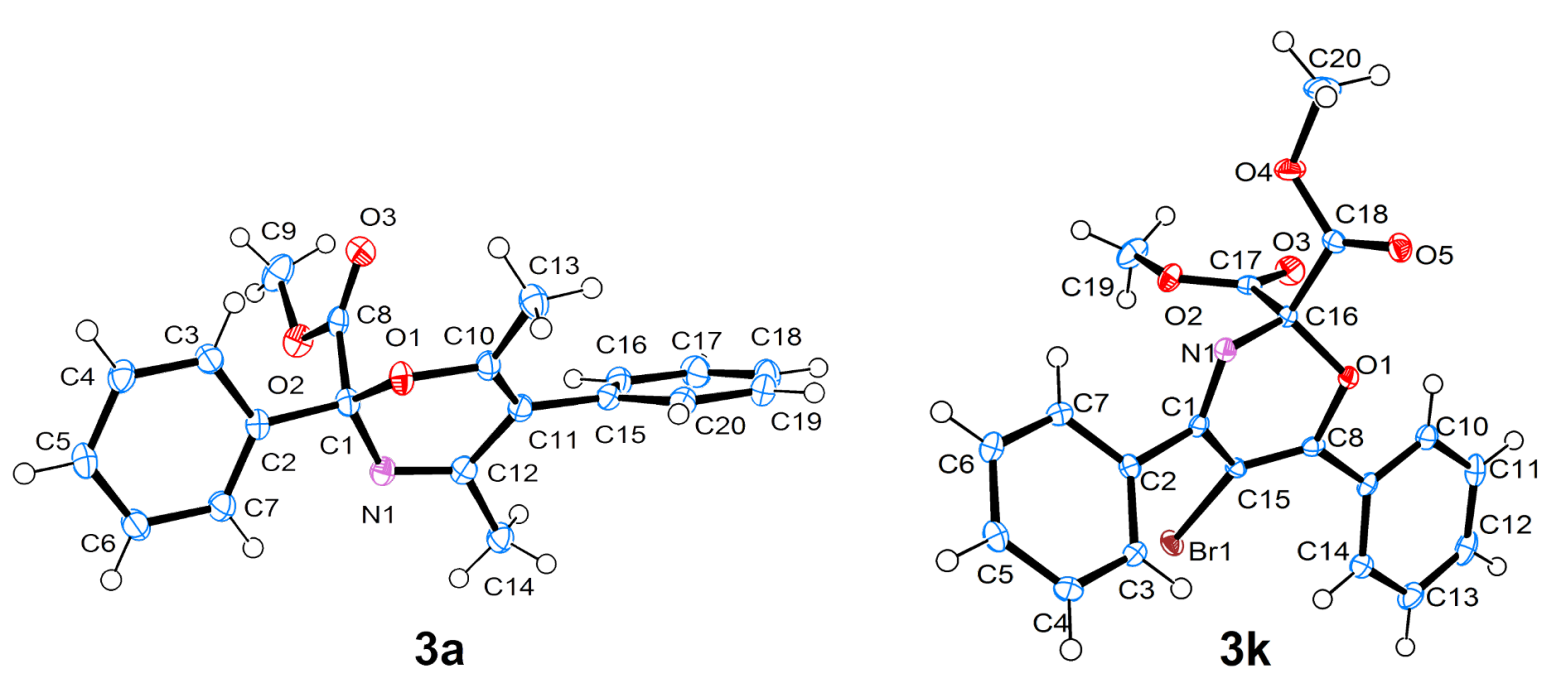
Molecular structures of compounds **3a**,**k**, displacement parameters are drawn at 50% probability level.

The structures of compounds **3** were verified by ^1^H NMR, ^13^C NMR, IR spectroscopy, HRMS, and elemental analysis. The structures of compounds **3a**,**k** were additionally confirmed by X-ray analysis (Figure 3).

The characteristic feature of the structure of compound **3a** is the pseudo-axial position of the methoxycarbonyl group. The structure of compound **3a** in the crystal corresponds to the most stable conformer according to calculations at DFT B3LYP/6-31G(d) level in vacuo (ΔΔ*G*^298 K^ (equatorial/axial = 1.2 kcal/mol). One of the possible reasons for the higher stability of conformer **3a** with a pseudo-axial methoxycarbonyl group in comparison to conformer **3a'** with a pseudo-equatorial methoxycarbonyl group, is assumed to be the anomeric effect [24]. The lengthening of the C–CO_2_Me bond in conformer **3a** compared to conformer **3a'** (1.563/1.554 Å), corroborates this hypothesis. The pseudo-axia position of the methoxycarbonyl group is preferred for all calculated oxazines.

In the reactions of diazo esters with 5-alkoxy-substituted isoxazoles **1f**,**h**, in contrast to isoxazoles **1a–e**, no formation of 1,3-oxazines was detected. Instead, the corresponding 1-oxa-5-azahexa-1,3,5-trienes **4a–f** were isolated in moderate to good yields (Table 3).

**Table 3:** Synthesis of 1-oxa-5-azahexa-1,3,5-trienes **4a–f**.

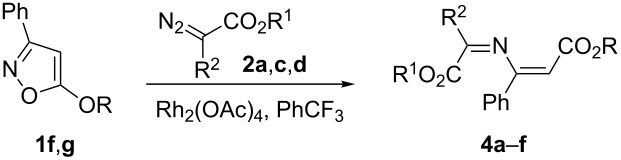					

**1**	**2**	R	R^1^	R^2^	**4**, yield,^a^ %

**f**	**a**	Me	Me	Ph	**4a** 51 (69)
**f**	**c**	Me	Me	CO_2_Me	**4b** 54 (61)
**f**	**d**	Me	Et	CO_2_Et	**4c** 80 (89)
**f**	**e**	Me	Et	CF_3_	**4d** 57
**g**	**c**	*t*-Bu	Me	CO_2_Me	**4e** 29 (45)
**g**	**d**	*t*-Bu	Et	CO_2_Et	**4f** 25 (38)

^a^Yields based on consumed isoxazole are listed in parentheses.

**Figure 4:**
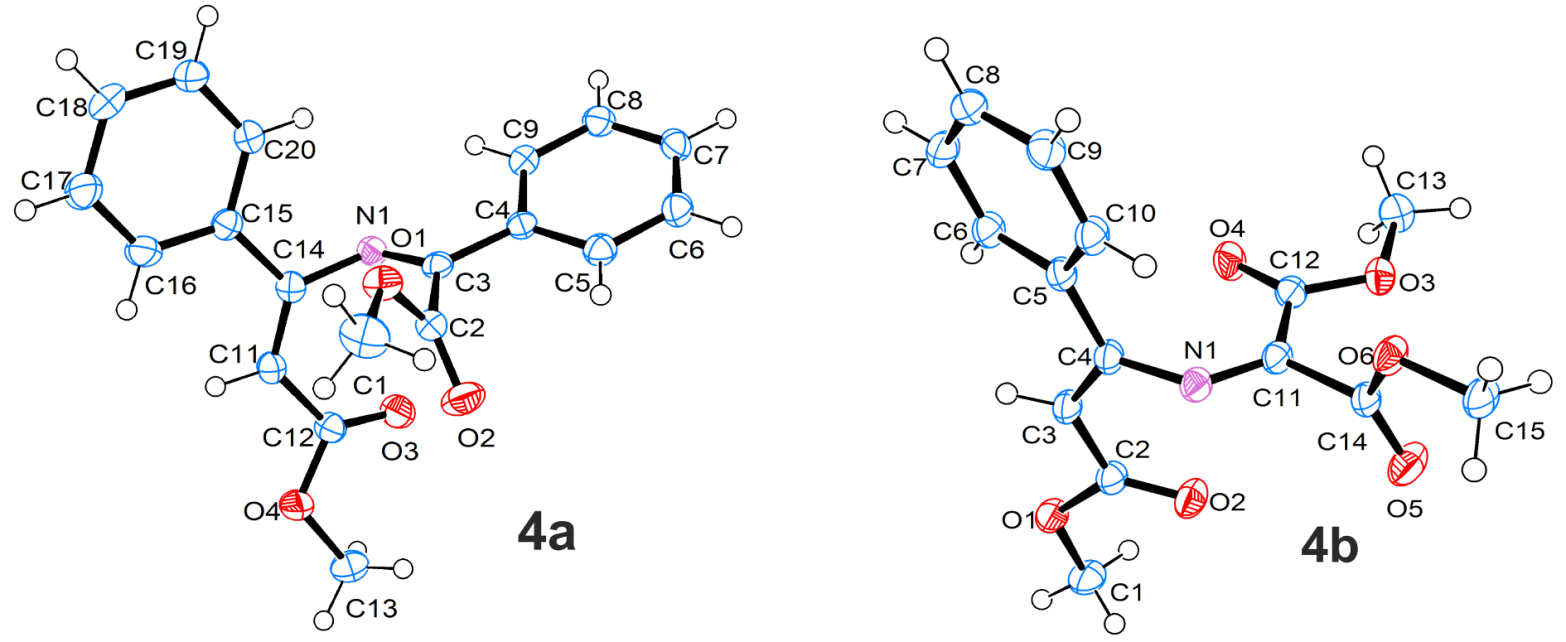
Molecular structures of compounds **4a**,**b**, displacement parameters are drawn at 50% probability level.

The structures of compounds **4a–f** were verified by ^1^H, ^13^C NMR, IR spectroscopy, and HRMS. Furthermore, the structures of compounds **4a**,**b** were confirmed by X-ray analysis (Figure 4). According to ^1^H NMR no corresponding 1,3-oxazines were formed.

**Scheme 4:**
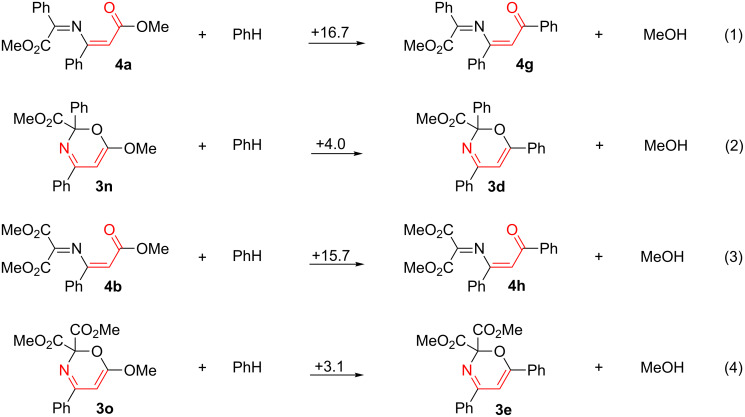
Isodesmic reactions for 1,3-oxazines **3d**,**e**,**n**,**o** and 1-oxa-5-azahexa-1,3,5-trienes **4a**,**b**,**g**,**h**.

Thus, only reactions of carbenoids with 5-alkoxy-substituted izoxazoles give the corresponding 1-oxa-5-azahexa-1,3,5-trienes instead of oxazines. To reveal the reason for this – either the destabilization of the oxazine or the stabilization of the 2-azabuta-1,3-diene when the phenyl group in **3d**,**e** or **4g**,**h** is exchanged for a methoxy group (compounds **4g**,**h** were not isolated) – the corresponding changes in Gibbs free energy were evaluated from isodesmic reactions 1–4 (Scheme 4). These calculations were based on the Gibbs free energy of the compounds, which were obtained by DFT B3LYP/6-31G(d) calculations (ΔΔ*G*^298 K^, kcal/mol).

Although the substitution of the Ph group to a MeO group in compounds **3d**,**e** results in a stabilization of the oxazines (Scheme 4, reactions 2 and 4), the formation of the corresponding 1-oxa-5-azahexa-1,3,5-trienes from 5-methoxy-substituted isoxazoles is mainly caused by the higher thermodynamic stability of 2-methoxy-substituted 1-oxa-5-azahexa-1,3,5-trienes **4a**,**b** compared to 2-phenyl-substituted 1-oxa-5-azahexa-1,3,5-trienes **4g**,**h** (Scheme 4, reactions 1 and 3). According to the X-ray analysis the R(MeO_2_C)C=N-group in compound **4** is not in conjugation with the remaining multiple bonds, that is, the methyl cinnamate conjugated system exerts a significantly greater influence on the stabilization than the corresponding chalcone system.

Thus, there is a good correspondence between the theoretical and experimental results, both of which support that Rh(II)-catalyzed reactions of diazo compounds with isoxazoles do not involve the formation of isoxazolium ylides but directly lead to the formation of azadienes, the latter can undergo a 6π-cyclization into the corresponding 1,3-oxazines. The position of the valence isomeric equilibrium depends on the relative thermodynamic stability of cyclic and acyclic isomers.

**Scheme 5:**
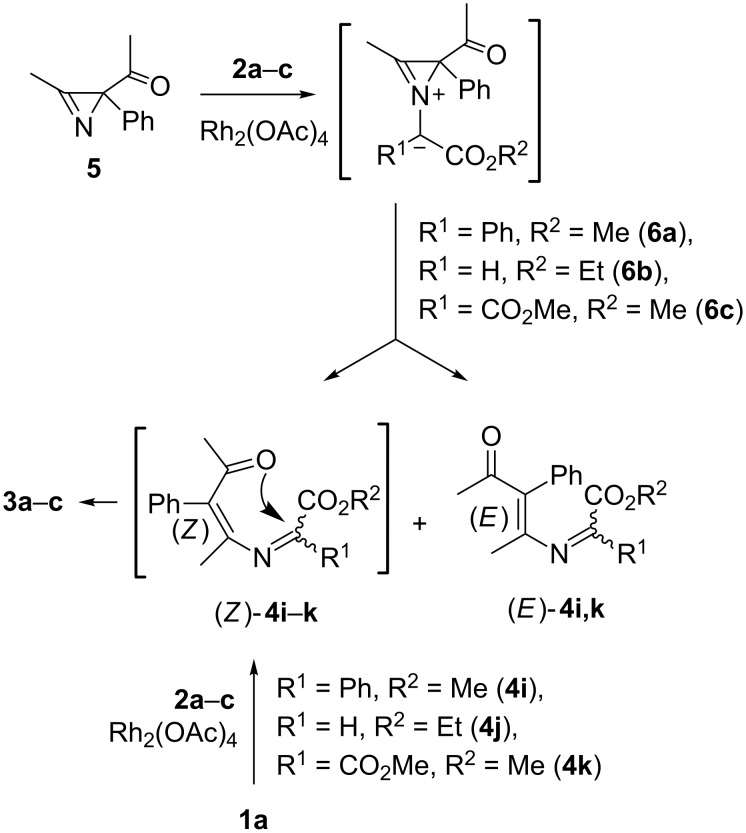
Reaction of complementary isoxazole **1a** and azirine **5** with diazo esters.

Additional evidence of a “one-step oxazahexatriene mechanism” of carbenoid-mediated isoxazole ring expansion originates from the results of the interaction of diazo compounds **2a**–**c** with the complimentary isoxazole **1a** and azirine **5** (Scheme 5). The reaction of isoxazoles with a carbenoid can only give a (3*Z*)-1-oxa-5-azahexa-1,3,5-triene due to geometrical restrictions. (3*Z*)-1-Oxa-5-azahexa-1,3,5-triene can then cyclize into the corresponding oxazine, so that the products of the reaction of isoxazole **1a** with diazo compounds **2a**–**c** were only oxazines **3a**–**c** (Table 2). In contrast, azirinium ylides **6a–c** formed by the reaction of azirine **5** with diazo compounds **2a**–**c** can transform into (3*Z*)- and (3*E*)-1-oxa-5-azahexa-1,3,5-triene **4i–k**, but only the former can cyclize into 1,3-oxazines **3a**–**c** (Scheme 5). In accordance with this, the reactions of azirine **5** with diazo compounds **2a**–**c** (3*E*)-1-oxa-5-azahexa-1,3,5-trienes (*E*)-**4i**,**k** were isolated as well as oxazines **3a**–**c** [10]. The corresponding ethyl 2-((*E*)-4-oxo-3-phenylpent-2-en-2-ylimino)acetate (*E*)-**4j** was not isolated from the reaction of azirine **5** with diazo compound **2b**, probably due to its instability.

## Conclusion

According to DFT calculations at the B3LYP/6-31G(d) level and experimental data the formation of 2*H*-1,3-oxazines from the reaction of isoxazoles with diazo compounds proceeds through an initial formation of (3*Z*)-1-oxa-5-azahexa-1,3,5-trienes that undergo 6π-cyclization. The stationary points corresponding to isoxazolium N-ylides from isoxazoles and methoxycarbonylcarbene were located only for derivatives without a substituent in position 3 of the isoxazole ring. These isoxazolium N-ylides are thermodynamically and kinetically very unstable and, therefore, there is a low probability to detect them, even though they may theoretically be formed. According to the calculations and experimental results 2*H*-1,3-oxazines are usually characterized by a greater thermodynamical stability than the corresponding open-chain isomers, (3*Z*)-1-oxa-5-azahexa-1,3,5-trienes. An exception is oxaazahexatrienes derived from 5-alkoxyisoxazoles which are thermodynamically more stable than the corresponding 2*H*-1,3-oxazines. Therefore, the reaction of diazo esters with 5-alkoxyisoxazoles is a good approach to yield 1,4-di(alkoxycarbonyl)-2-azabuta-1,3-dienes, which are useful building blocks in heterocyclic synthesis [25–26]. We found reaction conditions which allow for the preparation of aryl- and halogen-substituted 2*H*-1,3-oxazines as well as 1,4-di(alkoxycarbonyl)-2-azabuta-1,3-dienes starting from isoxazoles and diazo esters.

## Experimental

### General methods

Melting points were determined on a hot stage microscope and are uncorrected. ^1^H (300 MHz) and ^13^C (75 MHz) NMR spectra were determined in CDCl_3_ with a Bruker DPX 300 and a Bruker AVANCE III 400 spectrometer. Chemical shifts (δ) are reported in parts per million downfield from tetramethylsilane. Mass spectra were recorded on a Bruker maXis HRMS-ESI-QTOF by using electrospray ionization in the positive mode. IR spectra were recorded on a Bruker FTIR spectrometer Tensor 27 by using KBr disks and only characteristic absorption bands are indicated. Single crystal X-ray data were collected by means of an Agilent Technologies Supernova Atlas and an Agilent Technologies Excalibur Eos diffractometer. The crystals were kept at 100 K during data collection. The structures have been solved by the direct methods and refined by means of the SHELXL-97 program [27] incorporated in the OLEX2 program package [28]. Crystallographic data for the structures **3a** (CCDC 998319), **3k** (CCDC 998318), **4a** (CCDC 998317), **4b** (CCDC 998316) have been deposited with the Cambridge Crystallographic Data Centre. Isoxazoles **1a** [29], **1b** [30], **1c–e** [31], **1f**,**g** [32] were prepared by the reported procedures.

**General procedure of reacting isoxazoles with diazo compounds.** A long Schlenk tube containing a mixture of isoxazole (0.3–1.2 mmol) and diazo compound (1 equiv) in PhCF_3_ (1–2 mL) was put into an oil bath preheated to 110 °C. To the vigorously stirred mixture, Rh_2_(OAc)_4_ (1–5 mol %) was added in one portion and stirred until N_2_ evolution has been stopped (10–15 min). An additional amount of diazo compound was added dropwise, and then the mixture was heated for an additional 15 min. The reaction mixture was cooled, concentrated in vacuo, and the residue was separated by column chromatography on silica with a mixture of hexane/ethyl acetate as eluent.

#### Methyl 4,6-dimethyl-2,5-diphenyl-2*H*-1,3-oxazine-2-carboxylate (**3a**)

Compound **3a** (40 mg, 43%) was obtained from isoxazole **1a** (50 mg, 0.289 mmol), diazo ester **2a** (51 + 117 mg, 0.953 mmol) and Rh_2_(OAc)_4_ (3.8 mg, 3 mol %) in PhCF_3_ (1 mL). Colourless solid; mp 72–74 °C (CF_3_Ph); ^1^H NMR (400 MHz, CDCl_3_) δ 1.89 (s, 3H, Me), 1.96 (s, 3H, Me), 3.76 (s, 3H, MeO), 7.02–7.05 (m, 2H, Ar-H), 7.29–7.44 (m, 6H, Ar-H), 7.78–7.82 (m, 2H, Ar-H); ^13^C NMR (100 MHz, CDCl_3_) δ 17.4, 23.6, 53.0, 91.2, 116.2, 126.4, 127.4, 128.2, 128.5, 128.8, 130.3, 135.0, 138.9, 158.7, 165.5, 170.9; ESIMS (*m*/*z*): calculated for C_20_H_20_NO_3_^+^, 322.1438; found, 322.1444; Anal. calcd for C_20_H_19_NO_3_: C, 74.75; H, 5.96; N, 4.36; found: C, 75.04; H, 5.74; N, 4.64; IR (KBr, cm^−1^) ν: 1743 (C=O); crystal data for **3a**: C_20_H_19_NO_3_, *M* = 321.36, monoclinic, space group *P*2_1_/*n*, *a* = 10.2806(4), *b* = 10.5164(3), *c* = 15.3771(4) Å, β = 98.241(3)°, *V* = 1645.32(9) Å^3^, *Z* = 4, F(000) = 680, *D*_calc_ = 1.297 mg m^−3^, μ = 0.704 mm^−1^. 8556 reflections were collected yielding 3164 unique (*R*_int_ = 0.0191). The final *wR*_2_ = 0.1000 (all data) and *R*_1_ = 0.0354 for 2848 reflections with I ≥ 2σ, GOF = 1.030.

#### Ethyl 4,6-dimethyl-5-phenyl-2*H*-1,3-oxazine-2-carboxylate (**3b**)

Compound **3b** (52 mg, 35%) was obtained from isoxazole **1a** (100 mg, 0.577 mmol), diazo ester **2b** (66 + 58 mg, 1.09 mmol) and Rh_2_(OAc)_4_ (7.6 mg, 3 mol %) in PhCF_3_ (1 mL). Colorless solid; mp ca. 25 °C; ^1^H NMR (400 MHz, CDCl_3_) δ 1.37 (t, *J* = 7.1 Hz, 3H, Me), 1.83 (d, *J* = 0.9 Hz, 3H, Me), 1.85 (s, 3H, Me), 4.28–4.42 (m, 2H, CH_2_O), 5.60 (d, *J =* 0.9 Hz, 1H, 2-H), 7.11–7.13 (m, 2H, Ar-H), 7.32–7.39 (m, 3H, Ar-H); ^13^C NMR (100 MHz, CDCl_3_) δ 14.2, 17.0, 23.3, 62.0, 85.1, 116.0, 127.6, 128.6, 130.4, 135.1, 159.2, 166.2, 168.4; ESIMS (*m*/*z*): calcd for C_14_H_18_NO_3_^+^, 260.1281; found, 260.1281; IR (KBr, cm^−1^) ν: 1737 (C=O).

#### Methyl 2,4,6-triphenyl-2*H*-1,3-oxazine-2-carboxylate (**3d**)

Compound **3d** (245 mg, 66%; 70% based on consumed isoxazole) was obtained from isoxazole **1b** (221 mg, 1.00 mmol), diazo ester **2a** (176 + 224 mg, 2.27 mmol) and Rh_2_(OAc)_4_ (6.6 mg, 1.5 mol %) in PhCF_3_ (2 mL). Colorless solid; mp 114–115 °C (hexane/ether); ^1^H NMR (400 MHz, CDCl_3_) δ 3.72 (s, 3H, MeO), 6.69 (s, 1H, 5-H), 7.46–7.53 (m, 9H, Ar-H), 8.00–8.03 (m, 2H, Ar-H), 8.05–8.07 (m, 2H, Ar-H), 8.07–8.11 (m, 2H, Ar-H); ^13^C NMR (100 MHz, CDCl_3_, 323 K) δ 53.0, 93.2, 95.9, 126.7, 126.8, 127.2, 128.3, 128.5, 128.6, 129.0, 130.9, 131.3, 132.2, 136.7, 139.0, 161.5, 162.9, 170.8; ESIMS (*m*/*z*): calcd for C_24_H_20_NO_3_^+^, 370.1438; found, 370.1437; IR (KBr, cm^−1^) ν: 1735 (C=O).

In addition to the product starting isoxazole **1b** (11 mg) and MeO_2_C(Ph)C=N-N=C(Ph)CO_2_Me (104 mg, yellowish solid, mp 141**–**143 °C, ether (lit. [33]: mp 142**–**143 °C, MeOH)) were isolated.

#### Methyl 3-((*Z*)-(2-methoxy-2-oxo-1-phenylethylidene)amino)-3-phenylacrylate (**4a**)

Compound **4a** (139 mg, 51%; 69% based on consumed isoxazole) was obtained from isoxazole **1f** (175 mg, 1.00 mmol), diazo ester **2a** (176 + 224 mg, 2.27 mmol) and Rh_2_(OAc)_4_ (5 mg, 1.5 mol %) in PhCF_3_ (1 mL). Yellow solid; mp 83**–**85 °C (hexane/ether); ^1^H NMR (400 MHz, CDCl_3_) δ 3.66 (s, 3H, MeO), 3.76 (s, 3H, MeO), 5.66 (s, 1H, 2-H), 7.36–7.41 (m, 3H, Ar-H), 7.44–7.48 (m, 2H, Ar-H), 7.51–7.58 (m, 3H, Ar-H), 7.88–7.90 (m, 2H, Ar-H); ^13^C NMR (100 MHz, 323 K, CDCl_3_) δ 51.2, 52.1, 97.0, 126.5, 128.61, 128.62, 130.2, 131.9, 133.1, 135.7, 158.3, 160.8, 163.2, 166.1; ESIMS (*m*/*z*): calcd for C_19_H_18_NO_4_^+^, 324.1230; found, 324.1235; IR (KBr, cm^−1^) ν: 1743, 1714 (C=O); crystal data for **4a**: C_19_H_17_NO_4_, *M* = 326.36, monoclinic, space group *P*2_1_/*n*, *a* = 9.1055(2), *b* = 13.7411(3), *c* = 13.2422(2) Å, β = 101.397(2)°, *V* = 1624.19(5) Å^3^, *Z* = 4, F(000) = 692, *D*_calc_ = 1.335 mg m^−3^, μ = 0.766 mm^−1^. 21965 reflections were collected yielding 3407 unique (*R*_int_ = 0.0246). The final *wR*_2_ = 0.1033 (all data) and *R*_1_ = 0.0376 for 3207 reflections with I ≥ 2σ, GOF = 1.061.

#### Dimethyl (*Z*)-2-((3-methoxy-3-oxo-1-phenylprop-1-en-1-yl)imino)malonate (**4b**)

Compound **4b** (164 mg, 54%; 61% based on consumed isoxazole) was obtained from isoxazole **1f** (175 mg, 1.00 mmol), diazo ester **2c** (159 + 63 mg, 1.40 mmol) and Rh_2_(OAc)_4_ (5 mg, 1.5 mol %) in PhCF_3_ (1 mL). Yellow solid; mp 63**–**65 °C (hexane/ether); ^1^H NMR (400 MHz, CDCl_3_) δ 3.26 (s, 3H, MeO), 3.87 (br. s, 6H, MeO), 5.58 (s, 1H, 2’-H), 7.38–7.40 (m, 3H, Ar-H), 7.49–7.51 (m, 2H, Ar-H); ^13^C NMR (100 MHz, 323 K, CDCl_3_) δ 51.3, 53.1, 96.9, 126.5, 128.8, 130.6, 134.5, 150.4, 158.8, 160.3, 165.6; ESIMS (*m*/*z*): calcd for C_15_H_16_NO_6_^+^, 306.0972; found, 306.0979; IR (KBr, cm^−1^) ν: 1751, 1708 (C=O); Crystal data for **4b**: C_15_H_15_NO_6_, *M* = 305.28, triclinic, space group *P*-1, *a* = 7.2795(6), *b* = 9.9865(7), *c* = 10.7460(5) Å, α = 99.827(5), β = 93.454(6), γ = 105.608(7)°, *V* = 736.70(9) Å^3^, *Z* = 2, F(000) = 320, *D*_calc_ = 1.376 mg m^−3^, μ = 0.911 mm^−1^. 6917 reflections were collected yielding 2897 unique (*R*_int_ = 0.0627). The final *wR*_2_ = 0.2337 (all data) and *R*_1_ = 0.0596 for 2689 reflections with I ≥ 2σ, GOF = 0.986.

**Calculations.** All calculations were carried out at DFT B3LYP/6-31G(d) level [34–36] by using the Gaussian 09 suite of quantum chemical programs [37] at the Resource center ‘Computer center of Saint Petersburg State University’. Geometry optimizations of intermediates, transition states, reactants and products in benzene were performed by means of a PCM model. Intrinsic reaction coordinates were calculated to authenticate all transition states.

## Supporting Information

File 1 Detailed experimental procedures including characterization data for all synthesized compounds, ^1^H and ^13^C NMR spectra for all new compounds, and computational details (energies of molecules, transition states, and the Cartesian coordinates of atoms).

## References

[1] Baraldi P G, Barco A, Benetti S, Pollini G P, Simoni D (1987). Synthesis.

[2] Pinho e Melo T M V D (2005). Curr Org Chem.

[3] Hamama W S, Ibrahim M E, Zoorob H H (2013). Synth Commun.

[4] Manning J R, Davies H M L (2008). Tetrahedron.

[5] Manning J R, Davies H M L (2008). J Am Chem Soc.

[6] King J F, Durst T (1962). Can J Chem.

[7] Kashima C, Tsuda Y, Imada S, Nishio T (1980). J Chem Soc, Perkin Trans 1.

[8] DeShong P, Cipollina J A, Lowmaster N K (1988). J Org Chem.

[9] González-Nogal A M, Calle M (2009). Tetrahedron.

[10] Zavyalov K V, Novikov M S, Khlebnikov A F, Yufit D S (2013). Tetrahedron.

[11] Zavyalov K V, Novikov M S, Khlebnikov A F, Pakalnis V V (2014). Tetrahedron.

[12] Kai M, Furuhashi T, Masuzawa Y, Yano T, Saito F, Nakaya Y (2012). Haloalkylsulfonanilide derivative. U.S. Patent.

[13] Heine N, Fuchs K, Eickmeier C, Peters S, Dorner-Ciossek C, Handschuh S, Nar H, Klinder K (2010). Compounds for the treatment of alzheimer's disease. U.S. Patent.

[14] Claremon D A (2010). Cyclic inhibitors of 11beta-hydroxysteroid dehydrogenase 1. W.O. Patent.

[15] Dhar T G M, Yang G, Davies P, Malley M F, Gougoutas J Z, Wu D-R, Barrish J C, Carter P H (2009). Bioorg Med Chem Lett.

[16] Vintonyak V V, Calà M, Lay F, Kunze B, Sasse F, Maier M E (2008). Chem–Eur J.

[17] Condon J S, Joseph-McCarthy D, Levin J I, Lombart H-G, Lovering F E, Sun L, Wang W, Xu W, Zhang Y (2007). Bioorg Med Chem Lett.

[18] Fries K M, Joswig C, Borch R F (1995). J Med Chem.

[19] Marron B E, Fritch P C, Mark-Worth C J, Maynard A T, Swain N A (2008). Inhibitors of ion channels. W.O. Patent.

[20] Glatthar R, Orain D, Spanka C (2007). Nicotinic acid derivatives as modulators of metabotropic glutamate receptors. W.O. Patemt.

[21] Ross J A, Seiders R P, Lemal D M (1976). J Am Chem Soc.

[22] von Ragué Schleyer P, Wu J I, Cossio F P, Fernández I (2014). Chem Soc Rev.

[23] Ovalles S R, Hansen J H, Davies H M L (2011). Org Lett.

[24] Uehara F, Sato M, Kaneko C, Kurihara H (1999). J Org Chem.

[25] Monbaliu J-C M, Masschelein K G R, Stevens C V (2011). Chem Soc Rev.

[26] Jayakumar S, Ishar M P S, Mahajan M P (2002). Tetrahedron.

[27] Sheldrick G M (2008). Acta Crystallogr, Sect A.

[28] Dolomanov O V, Bourhis L J, Gildea R J, Howard J A K, Puschmann H (2009). J Appl Crystallogr.

[29] Bobranski B, Wojtowski R (1964). Rocz Chem.

[30] Takikawa H, Takada A, Hikita K, Suzuki K (2008). Angew Chem, Int Ed.

[31] Day R A, Blake J A, Stephens C E (2003). Synthesis.

[32] Micetich R G, Chin C G (1970). Can J Chem.

[33] Singh B, Ulman E F (1967). J Am Chem Soc.

[34] Becke A D (1993). J Chem Phys.

[35] Becke A D (1998). Phys Rev A.

[36] Lee C, Yang W, Parr R G (1988). Phys Rev B.

[37] (2013). Gaussian 09.

