# Supplementary material for: Isoxazolium N-ylides and 1-oxa-5-azahexa-1,3,5-trienes on the way from isoxazoles to 2H-1,3-oxazines
Source: Beilstein J Org Chem. 2014 Aug 14;10:1896–905. doi: 10.3762/bjoc.10.197 (PMC4168923; doi:10.3762/bjoc.10.197)
Supplement: File 1 — Detailed experimental procedures including characterization data for all synthesized compounds, 1H and 13C NMR spectra for all new compounds, and computational details (energies of molecules, transition states, and the Cartesian coordinates of atoms). [file Beilstein_J_Org_Chem-10-1896-s001.pdf]

Supporting Information

for

Isoxazolium N-ylides and 1-oxa-5-azahexa-1,3,5-trienes on the way from isoxazoles to 2*H*-1,3-oxazines

Alexander F. Khlebnikov,* Mikhail S. Novikov, Yelizaveta G. Gorbunova, Ekaterina E. Galenko, Kirill I. Mikhailov, Viktoriia V. Pakalnis and Margarita S. Avdontceva

Address: Institute of Chemistry, Saint-Petersburg State University, Universitetskii pr. 26, 198504 St. Petersburg, Russia

Email: Alexander F. Khlebnikov - alexander.khlebnikov@pobox.spbu.ru

*Corresponding author

Detailed experimental procedures and computational details.

Table of contents:

Pages S2–S7: Detailed experimental procedures including characterization data for all sythesized compounds.

Pages S8–S43: ^{1}H and ^{13}C NMR spectra of new compounds.

Pages S44–S71: Computational details.

General procedure of reaction isoxazoles with diazo compounds. A long tube with a mixture of isoxazole (0.3-1.2 mmol) and diazo compound (1 equiv) in $PhCF_3$ (1-2 mL) was put into an oil bath preheated to 110 °C. To the vigorously stirred mixture, $Rh_2(OAc)_4$ (1-5 mol%) was added in one portion and after stopping N_2 evolution (10-15 min), an additional amount of diazo compound was added drop-wise, and then the mixture was heated additionally 15 min. The reaction mixture was cooled, concentrated *in vacuo* and the residue was separated by column chromatography on silica, eluent a mixture of hexane – ethyl acetate.

Dimethyl 4,6-dimethyl-5-phenyl-2*H*-1,3-oxazine-2,2-dicarboxylate (3c) [1]

Compound **3c** (117 mg, 67%) was obtained from isoxazole **1a** (100 mg, 0.577 mmol), diazo ester **2c** (91+18 mg, 0.692 mmol) and $Rh_2(OAc)_4$ (2.5 mg, 1 mol.%) in $PhCF_3$ (1 mL). Colorless solid, mp 103–104 °C (hexane-ether). ^{1}H NMR (300 MHz, $CDCl_3$): δ 1.88 s (3H, Me), 1.89 s (3H, Me), 3.89 s (6H, MeO), 7.06–7.08 m (2H, Ar-H), 7.32–7.36 m (3H, Ar-H). ^{13}C NMR (75 MHz, $CDCl_3$): δ 17.1, 23.6, 53.5, 90.2, 115.9, 127.8, 128.6, 130.2, 134.3, 158.3, 166.7, 166.9. ESI/MS (m/z): 304.1179 calcd for $C_{16}H_{18}NO_5^+$, found 304.1184. IR (KBr, cm^{-1}): ν 1750 (C=O).

Ethyl 4,6-diphenyl-2*H*-1,3-oxazine-2-carboxylate (3e)

Compound **3e** (97 mg, 34%) was obtained from isoxazole **1b** (205 mg, 0.927 mmol), diazo ester **2b** (105+225 mg, 2.90 mmol) and $Rh_2(OAc)_4$ (6 mg, 1.5 mol.%) in $PhCF_3$ (mL). Yellowish oil. ^{1}H NMR (400 MHz, $CDCl_3$): δ 1.35 t (3H, *J* 7.1, Me), 4.31–4.39 m (2H, CH_2O), 6.10 s (1H, 2-H), 6.62 s (1H, 5-H), 7.44–7.50 m (6H, Ar-H), 7.85–7.87 m (2H, Ar-H), 7.91–7.93 m (2H). ^{13}C NMR (100 MHz, $CDCl_3$): δ 14.2, 61.9, 86.8, 95.7, 126.5, 127.0, 128.5, 128.6, 130.9, 131.3, 131.8, 136.5, 161.6, 163.8, 168.3. ESI/MS (m/z): 308.1281 calcd for $C_{19}H_{18}NO_3^+$, found 308.1284. IR (KBr, cm^{-1}): ν 1745 (C=O).

Dimethyl 4,6-diphenyl-2*H*-1,3-oxazine-2,2-dicarboxylate (3f)

Compound **3f** (111 mg, 81%) was obtained from isoxazole **1b** (86 mg, 0.389 mmol), diazo ester **2c** (62+147 mg, 1.32 mmol) and $Rh_2(OAc)_4$ (4 mg, 2.5 mol.%) in $PhCF_3$ (1 mL). Colorless solid, mp 82–84 °C (hexane-ether). ^{1}H NMR (300 MHz, $CDCl_3$): δ 3.89 s (6H, Me), 6.63 s (1H, 5-H),

7.46–7.51 m (6H, Ar-H), 7.90–7.92 m (2H, Ar-H), 7.96–7.99 m (2H, Ar-H). ^{13}C NMR (75 MHz, $CDCl_3$): δ 53.6, 92.3, 95.8, 127.0, 127.3, 128.6, 128.7, 131.3, 131.4, 131.7, 136.0, 160.8, 163.9, 167.0. ESI/MS (m/z): 352.1179 calcd for $C_{20}H_{18}NO_5^+$, found 352.1182. Anal. calcd for $C_{20}H_{17}NO_5$: C 68.30; H 4.67; N 4.15. Found: C 68.37, H 4.88, N 3.99. IR (KBr, cm^{-1}): ν 1763, 1740 (C=O).

Ethyl 5-chloro-4,6-diphenyl-2*H*-1,3-oxazine-2-carboxylate (3g)

Compound **3g** (66 mg, 27%; 73 % on consumed isoxazole) was obtained from isoxazole **1c** (213 mg, 0.835 mmol), diazo ester **2b** (95+257 mg, 3.09 mmol) and $Rh_2(OAc)_4$ (11 mg, 3.0 mol.%) in $PhCF_3$ (2 mL). Yellowish oil. ^{1}H NMR (400 MHz, $CDCl_3$): δ 1.36 t (3H, *J* 7.1 Hz, Me), 4.34–4.40 m (2H, CH_2O), 5.97s (1H, 2-H), 7.45–7.50 m (6H, Ar-H), 7.70–7.72 m (2H, Ar-H), 8.01–8.03 m (2H, Ar-H). ^{13}C NMR (100 MHz, $CDCl_3$): δ 14.1, 62.3, 85.1, 107.0, 128.0, 128.2, 129.2, 130.1, 130.4, 130.5, 131.5, 135.5, 158.1, 165.4, 167.6. ESI/MS (m/z): 342.0891 calcd for $C_{19}H_{17}ClNO_3^+$, found 342.0893. IR (KBr, cm^{-1}): ν 1746 (C=O).

Dimethyl 5-chloro-4,6-diphenyl-2*H*-1,3-oxazine-2,2-dicarboxylate (3h)

Compound **3h** (73 mg, 48%) was obtained from isoxazole **1c** (100 mg, 0.392 mmol), diazo ester **2c** (62+55 mg, 0.745 mmol) and $Rh_2(OAc)_4$ (4 mg, 3.5 mol.%) in $PhCF_3$ (1 mL). Colorless solid, mp 79–81 °C (hexane). ^{1}H NMR (300 MHz, $CDCl_3$): δ 3.92 s (6H, Me), 7.46–7.51 m (6H, Ar-H), 7.75–7.76 m (2H, Ar-H), 8.06–8.09 m (2H, Ar-H). ^{13}C NMR (75 MHz, $CDCl_3$): δ 53.9, 90.6, 107.4, 128.0, 128.3, 129.4, 130.0, 130.5, 130.7, 131.8, 135.1, 156.9, 165.7, 166.3. ESI/MS (m/z): 386.0790 calcd for $C_{20}H_{17}ClNO_5^+$, found 386.0794. IR (KBr, cm^{-1}): ν 1741 (C=O).

Ethyl 5-bromo-4,6-diphenyl-2*H*-1,3-oxazine-2-carboxylate (3i)

Compound **3i** (48 mg, 19%; 75 % on consumed isoxazole) was obtained from isoxazole **1d** (100 mg, 0.334 mmol), diazo ester **2b** (38+114 mg, 1.34 mmol) and $Rh_2(OAc)_4$ (4.6 mg, 3.1 mol.%) in $PhCF_3$ (1 mL). Yellowish solid, mp *ca*. 25 °C. ^{1}H NMR (400 MHz, $CDCl_3$): δ 1.37 t (3H, *J* 7.1, Me), 4.33–4.41 m (2H, CH_2O), 5.98 s (1H, 2-H), 7.45–7.50 m (6H, Ar-H), 7.68–7.71 m (2H, Ar-H), 7.98–8.01 m (2H, Ar-H). ^{13}C NMR (100 MHz, $CDCl_3$): δ 14.2, 62.3, 85.4, 94.7, 127.9, 128.2,

129.2, 130.3, 130.5, 131.5, 136.6, 159.9, 165.9, 167.6. ESI/MS (m/z): 386.0386 calcd for $C_{19}H_{17}BrNO_3^+$, found 386.0374. IR (KBr, cm^{-1}): ν 1746 (C=O).

Dimethyl 5-bromo-4,6-diphenyl-2*H*-1,3-oxazine-2,2-dicarboxylate (3k)

Compound **3k** (30 mg, 21%) was obtained from isoxazole **1d** (100 mg, 0.334 mmol), diazo ester **2c** (53+26 mg, 0.501mmol) and $Rh_2(OAc)_4$ (4.3 mg, 3.0 mol.%) in $PhCF_3$ (1 mL). Colorless solid, mp 118-120 °C (hexane). ^{1}H NMR (400 MHz, $CDCl_3$): δ 3.92 s (6H, MeO), 7.45–7.53 m (6H, Ar-H), 7.74–7.76 m (2H, Ar-H), 8.06–8.08 m (2H, Ar-H). ^{13}C NMR (100 MHz, $CDCl_3$): δ 53.9, 90.8, 95.0, 127.9, 128.2, 129.4, 130.6, 130.8, 130.9, 131.8, 136.3, 158.7, 166.3. ESI/MS (m/z): 430.0285 calcd for $C_{20}H_{17}BrNO_5^+$, found 430.0285. IR (KBr, cm^{-1}): ν 1774, 1739 (C=O). Crystal data for **3k**: $C_{20}H_{16}BrNO_5$, M =430.25, monoclinic, space group $P2_1/c$, a = 9.0126(1), b = 23.3425(2), c = 9.6865(1) Å, β = 116.224(1)°, V = 1828.07(3) $Å^3$, Z = 4, F(000) = 872, D_{calc} = 1.563 mg m^{-3}, μ = 3.348 mm^{-1}. 23245 reflections were collected yielding 3450 unique (R_{int} = 0.0172). The final wR_2 = 0.0587 (all data) and R_1 = 0.0252 for 3448 reflections with $I \geq 2\sigma$, GOF = 1.023.

Ethyl 5-iodo-4,6-diphenyl-2*H*-1,3-oxazine-2-carboxylate (3l)

Compound **3l** (48 mg, 22%; 63 % on consumed isoxazole) was obtained from isoxazole **1e** (207 mg, 0.609 mmol), diazo ester **2b** (69+154 mg, 1.96 mmol) and $Rh_2(OAc)_4$ (3.9 mg, 1.5 mol.%) in $PhCF_3$ (1 mL). Yellow oil. ^{1}H NMR (400 MHz, $CDCl_3$): δ 1.37 t (3H J 7.1, Me), 4.37 k (2H, J 7.1, CH_2O), 5.96 s (1H, 2-H), 7.43–7.52 m (6H, Ar-H), 7.65–7.68 m (2H, Ar-H), 7.94–7.96 m (2H, Ar-H). ^{13}C NMR (100 MHz, $CDCl_3$): δ 14.1, 62.3, 64.5, 85.8, 127.9, 128.2, 129.2, 130.2, 130.9, 131.6, 133.2, 138.3, 163.5, 167.6, 167.7. ESI/MS (m/z): 434.0248 calcd for $C_{19}H_{17}INO_3^+$, found 434.0250. IR (KBr, cm^{-1}): ν 1741 (C=O).

Dimethyl 5-iodo-4,6-diphenyl-2*H*-1,3-oxazine-2,2-dicarboxylate (3m)

Compound **3m** (30 mg, 21%; 36 % on consumed isoxazole) was obtained from isoxazole **1e** (102 mg, 0.294 mmol), diazo ester **2c** (46+37 mg, 0.529 mmol) and $Rh_2(OAc)_4$ (3.8 mg, 3.0 mol.%) in $PhCF_3$ (1 mL). Colorless solid, mp 132-136 °C (hexane-ether). ^{1}H NMR (300 MHz,

$CDCl_3$): δ 3.92 s (6H, MeO), 7.45–7.53 m (6H, Ar-H),7.69–7.72 m (2H, Ar-H), 8.02–8.04 m (2H, Ar-H). ^{13}C NMR (75 MHz, $CDCl_3$): δ 53.9, 64.8, 91.3, 127.9, 128.2, 129.4, 130.5, 131.2, 131.9, 132.7, 138.1, 162.2, 166.4, 168.2. ESI/MS (m/z): 478.0146 calcd for $C_{20}H_{17}INO_5^+$, found 478.0150. IR (KBr, cm^{-1}): ν 1770, 1737 (C=O).

Diethyl (*Z*)-2-[(3-methoxy-3-oxo-1-phenylprop-1-en-1-yl)imino]malonate (4c)

Compound **4c** (537 mg, 80%; 89 % on consumed isoxazole) was obtained from isoxazole **1f** (350 mg, 2.00 mmol), diazo ester **2d** (376+94 mg, 2.50 mmol) and $Rh_2(OAc)_4$ (10 mg, 1.5 mol.%) in $PhCF_3$ (1 mL). Yellow oil. ^1H NMR (400 MHz, $CDCl_3$): δ 1.33 br s (6H, Me); 3.72 s (3H, MeO); 4.37 br s (4H, CH_2O); 5.62 s (1H, 2-H); 7.41-7.45 m (3H, Ar-H), 7.53-7.54 m (2H, Ar-H). ^{13}C NMR (100 MHz, $CDCl_3$): δ 13.9, 62.7, 51.4, 97.0, 126.6 128.8, 130.6, 134.4, 151.1, 158.8, 159.9, 165.6. ESI/MS (m/z): 334.1285 calcd for $C_{17}H_{20}NO_6^+$, found 334.1290. IR (KBr, cm^{-1}): ν 1745, 1710 (C=O).

Methyl 3-[(*Z*)-(3-ethoxy-1,1,1-trifluoro-3-oxopropan-2-ylidene)amino]-3-phenylacrylate (4d)

Compound **4d** (375 mg, 57%) was obtained from isoxazole **1f** (350 mg, 2.00 mmol), diazo ester **2e** (365+155 mg, 2.85 mmol) and $Rh_2(OAc)_4$ (10 mg, 1.5 mol.%) in $PhCF_3$ (1 mL). Yellow oil. ^1H NMR (400 MHz, $CDCl_3$): δ 1.28 t (3H, *J* 7.1, Me); 3.71 s (3H, MeO); 4.30 q (2H, *J* 7.1, CH_2O); 5.57 s (1H, 2-H); 7.44-7.48 m (3H, Ar-H), 7.53-7.55 m (2H, Ar-H). ^{13}C NMR (100 MHz, $CDCl_3$): δ 13.7, 51.4, 63.1, 95.7, 117.9 ($J_{C\text{-}F}$ 279.0), 126.4, 128.9, 130.8, 134.3, 146.6 ($J_{C\text{-}F}$ 36.7), 155.8, 158.0, 165.6. ESI/MS (m/z): 330.0948 calcd for $C_{15}H_{15}F_3NO_4^+$, found 330.0953. IR (KBr, cm^{-1}): ν 1747, 1714 (C=O).

Dimethyl 2-[(*Z*)-(3-(*tert*-butoxy)-3-oxo-1-phenylprop-1-en-1-yl)imino]malonate (4e)

Compound **4e** (199 mg, 28%; 44% on consumed isoxazole) was obtained from isoxazole **1g** (444 mg, 2.04 mmol), diazo ester **2c** (411+384 mg, 4.96 mmol) and $Rh_2(OAc)_4$ (10 mg, 1.5 mol.%) in $PhCF_3$ (1 mL). Yellow oil. ^1H NMR (400 MHz, $CDCl_3$): δ 1.48 s (9H, Me); 3.92 br s (6H, MeO); 5.52 s (1H, 2-H), 7.39-7.42 m (3H, Ar-H), 7.49-7.59 m (2H, Ar-H). ^{13}C NMR (100 MHz,

$CDCl_3$): δ 28.0, 53.1 br, 80.6, 100.1, 126.4, 128.7, 130.3, 134.6, 150.3, 157.1, 164.4. ESI/MS (m/z): 348.1442 calcd for $C_{18}H_{22}NO_6^+$, found 348.1447. IR (KBr, cm^{-1}): ν 1750, 1705 (C=O).

Diethyl 2-[(*Z*)-(3-(*tert*-butoxy)-3-oxo-1-phenylprop-1-en-1-yl)imino]malonate (4f)

Compound **4f** (185 mg, 25%; 38 % on consumed isoxazole) was obtained from isoxazole **1g** (423 mg, 1.95 mmol), diazo ester **2d** (313+297 mg, 3.80 mmol) and $Rh_2(OAc)_4$ (10 mg, 1.5 mol.%) in $PhCF_3$ (1 mL). Yellow oil. ^{1}H NMR (400 MHz, $CDCl_3$): δ 1.33 br s (6H, Me);1.49 s (9H, Me), 4.36 br s (4H, CH_2O), 5.52 s (1H, 2-H), 7.38-7.41 m (3H, Ar-H), 7.49-7.52 m (2H, Ar-H). ^{13}C NMR (100 MHz, $CDCl_3$): δ 13.9, 28.0, 62.6, 80.6, 100.4, 126.5, 128.6, 130.2, 134.7, 150.9, 156.9, 164.4. ESI/MS (m/z): 362.1234 calcd for $C_{20}H_{26}NO_6^+$, found 362.1240. IR (KBr, cm^{-1}): ν 1746 (C=O).

General procedure of reaction azirine 5 with diazo esters 2a-c. A long tube with a mixture of isoxazole and $Rh_2(OAc)_4$ (1.5-3 mol%) in $PhCF_3$ (1 mL) was put into an oil bath preheated to 110 °C. To the vigorously stirred mixture, the diazo compound was added drop-wise for 10 min and the mixture was stirred for an additional 15 min at the same temperature. The reaction mixture was cooled concentrated *in vacuo* and the residue was separated by column chromatography on silica.

Oxazine **3a** (92 mg, 50%) and methyl 2-(((*E*)-1-methyl-2-phenyl-3-oxobut-1-enyl)imino)-2-phenylacetate (*E*)-**4i** (29 mg, 16%) were isolated from the reaction of azirine **5** (100 mg, 0.577 mmol) and diazo ester **2a** (162 mg, 0.867 mmol).

Compound (*E*)-**4i**. Yellow oil. ^{1}H NMR (400 MHz, $CDCl_3$): δ 2.01 s (3H, Me), 2.39 s (1H, Me), 3.83 s (1H, MeO), 7.08–7.18 m (3H, Ar-H), 7.23–7.27 m (2H, Ar-H), 7.30–7.34 m (2H, Ar-H), 7.40–7.43 m (3H). ^{13}C NMR (100 MHz, $CDCl_3$): δ 20.0, 31.2, 52.3, 121.3, 127.0, 128.0, 128.2, 128.4, 130.0, 131.5, 133.3, 137.5, 155.3, 156.1, 163.2, 200.3. ESI/MS (m/z): 322.1438 calcd for $C_{20}H_{20}NO_3^+$, found 322.1438. IR (KBr, cm^{-1}): ν 1737, 1670 (C=O).

Oxazine **3b** (21 mg, 14%) was isolated from the reaction of azirine **5** (100 mg, 0.577 mmol) and diazo ester **2b** (73 mg, 0.647 mmol).

Oxazine **3c** (51 mg, 29%) and compound (*E*)-**4k** [1] (37 mg, 21%) were isolated from the reaction of azirine **5** (100 mg, 0.577 mmol) and diazo ester **2c** (92 mg, 0.582 mmol).

References

[1] Zavyalov, K. V.; Novikov, M. S.; Khlebnikov, A. F.; Yufit, D. S. *Tetrahedron* **2013**, *69*, 4546.

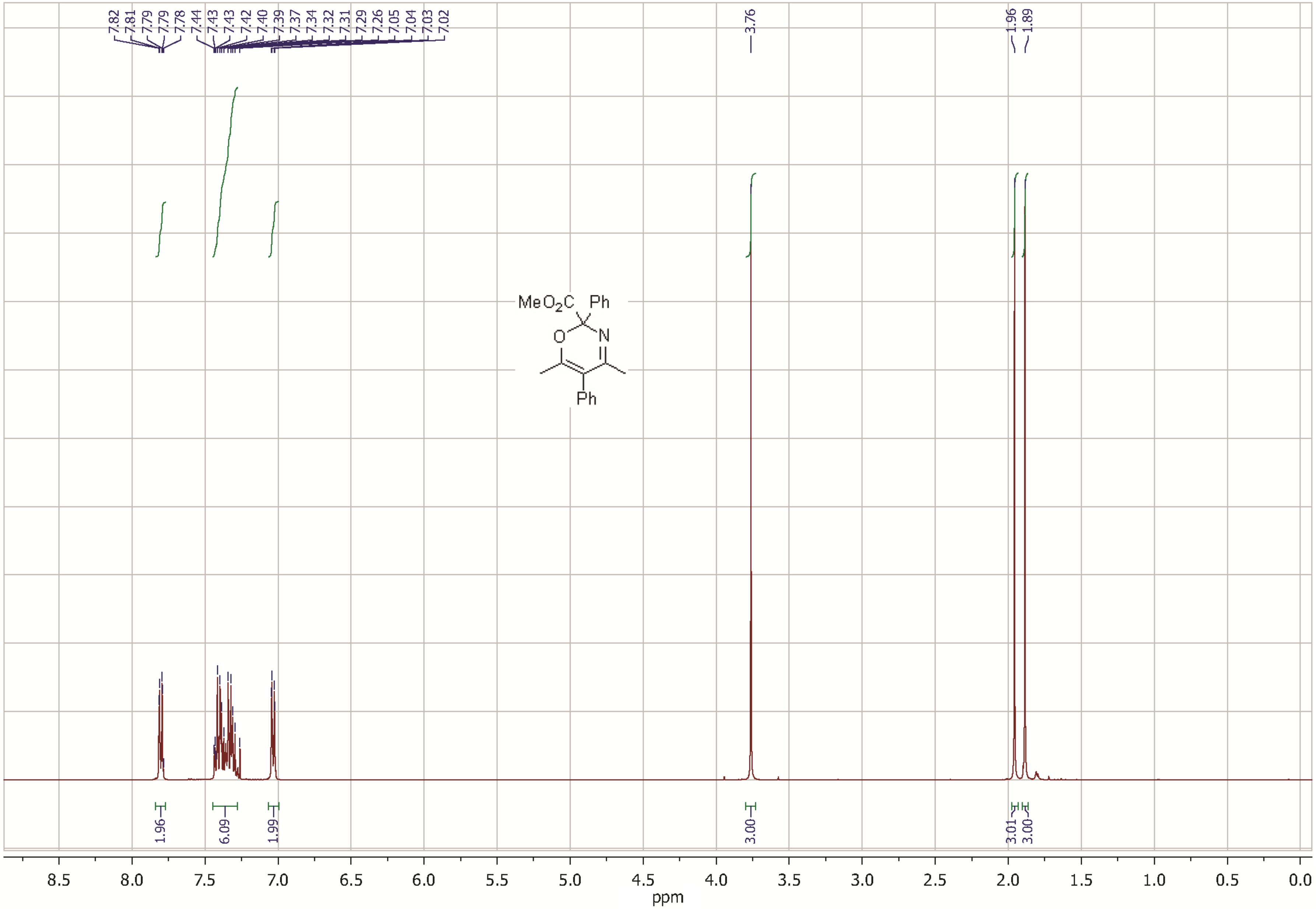

^{1}H NMR (400 MHz, $CDCl_3$) spectrum of compound **3a**.

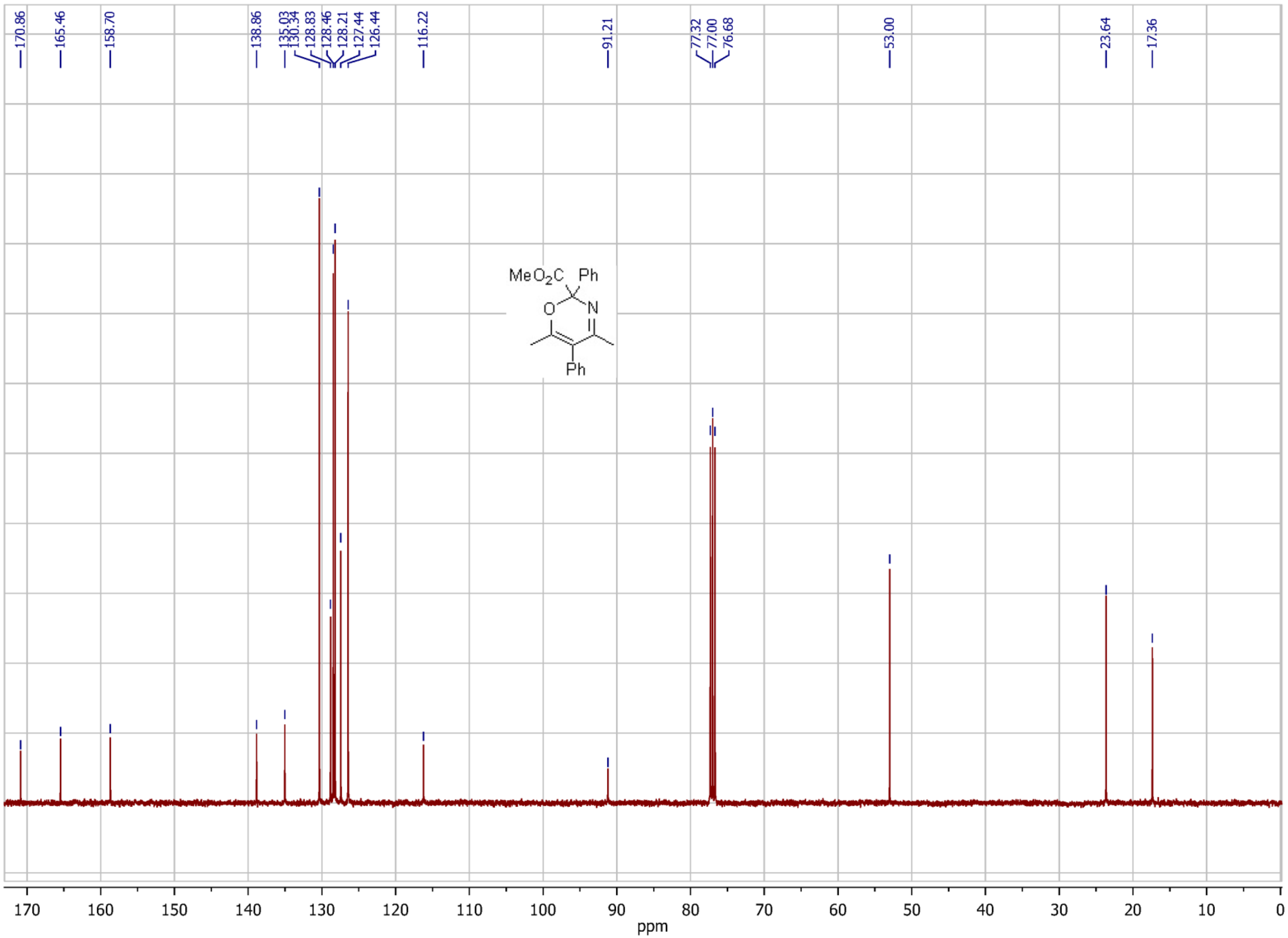

^{13}C NMR (100 MHz, $CDCl_3$)spectrum of compound **3a**.

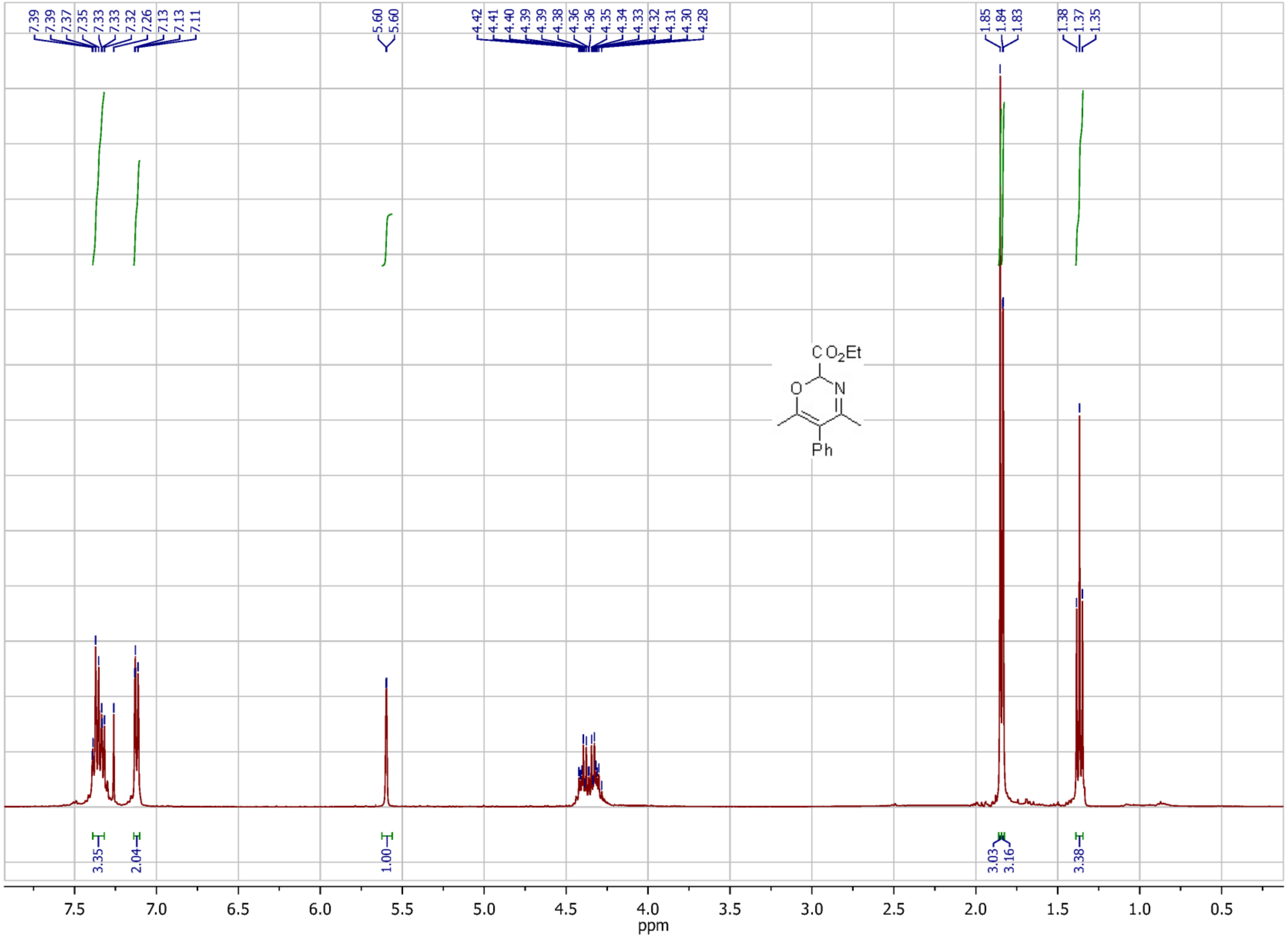

^{1}H NMR (400 MHz, $CDCl_3$)spectrum of compound **3b**.

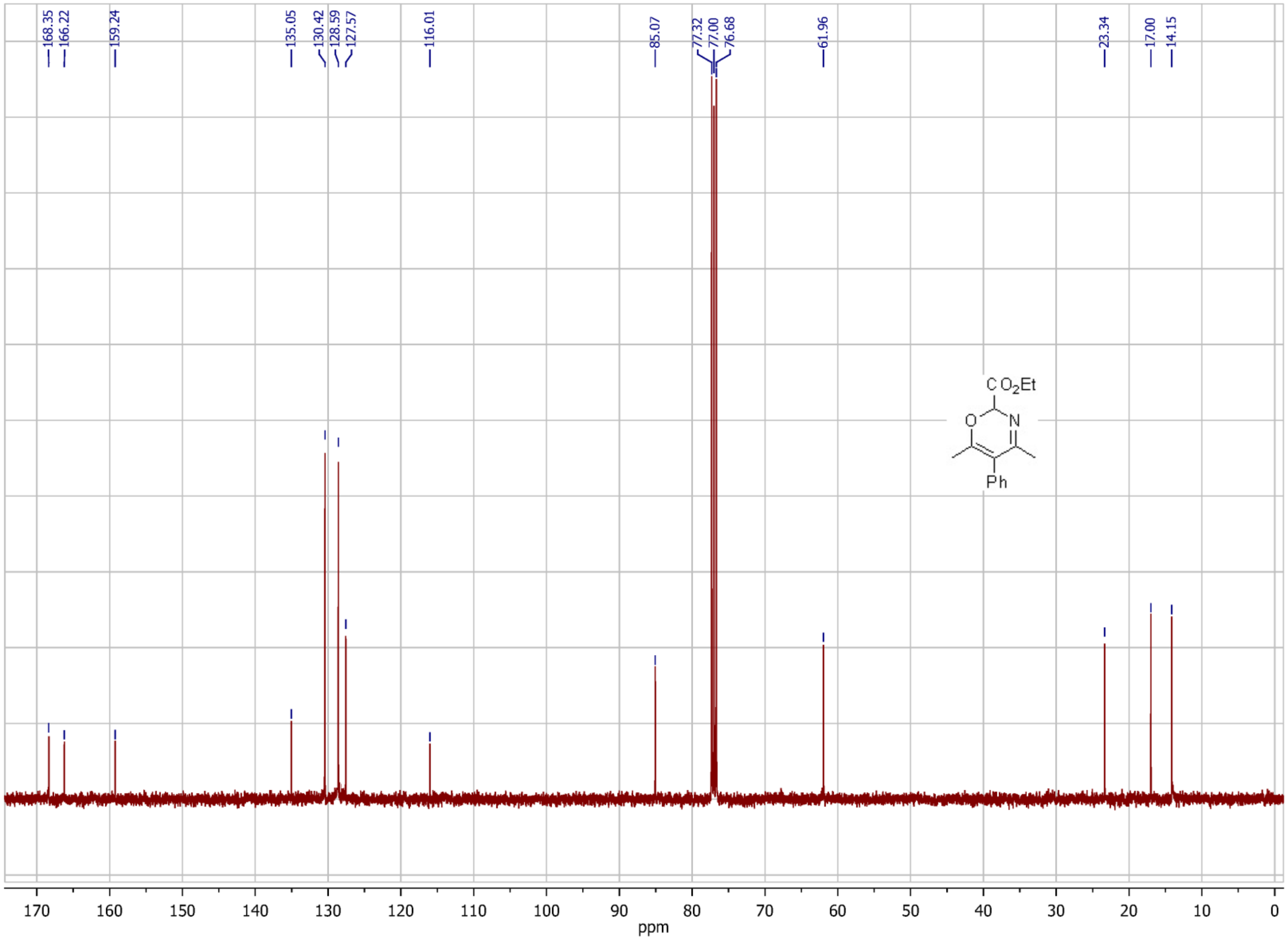

^{13}C NMR (100 MHz, $CDCl_3$)spectrum of compound **3b**.

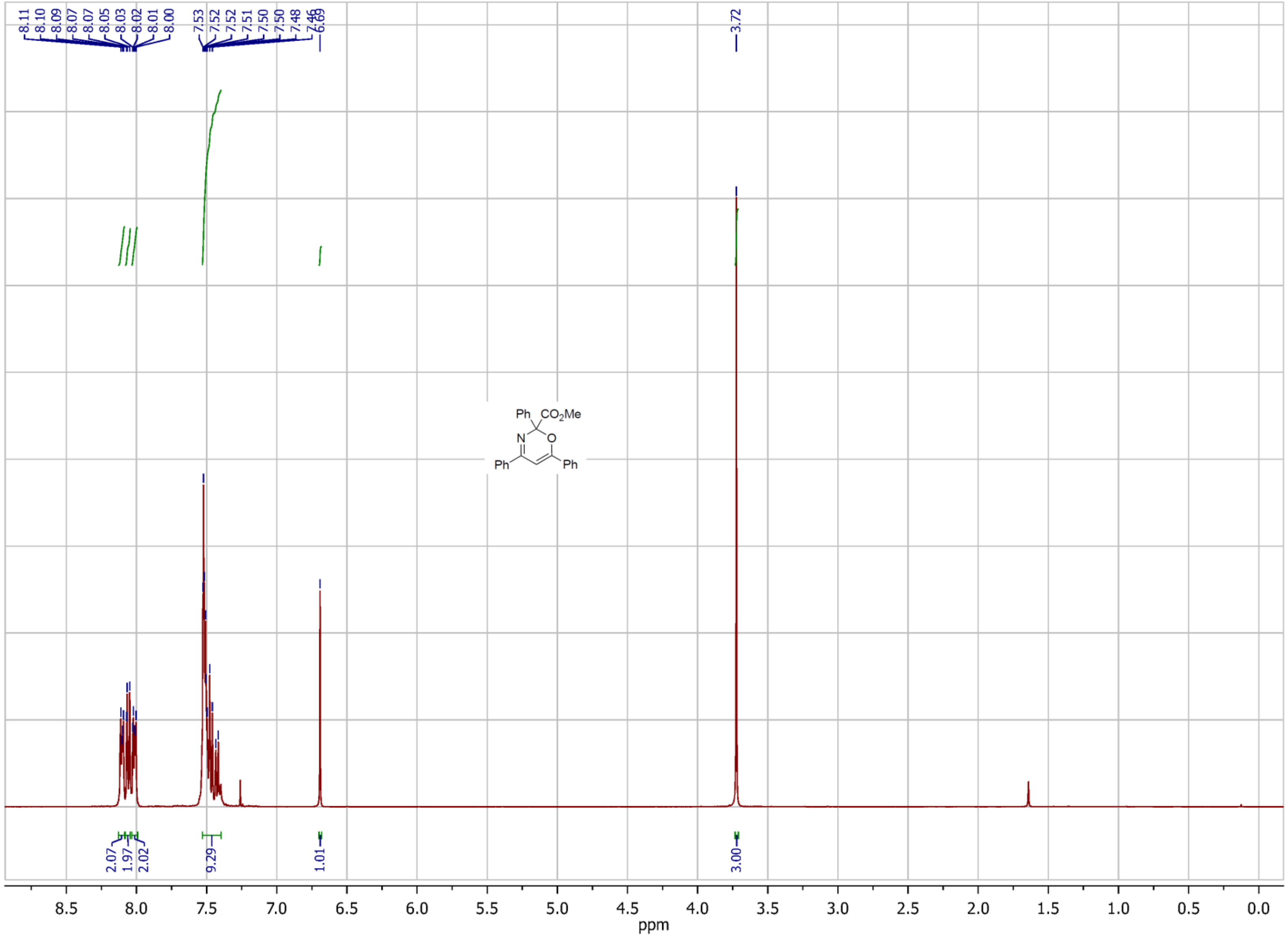

^{1}H NMR (400 MHz, $CDCl_3$) spectrum of compound **3d**.

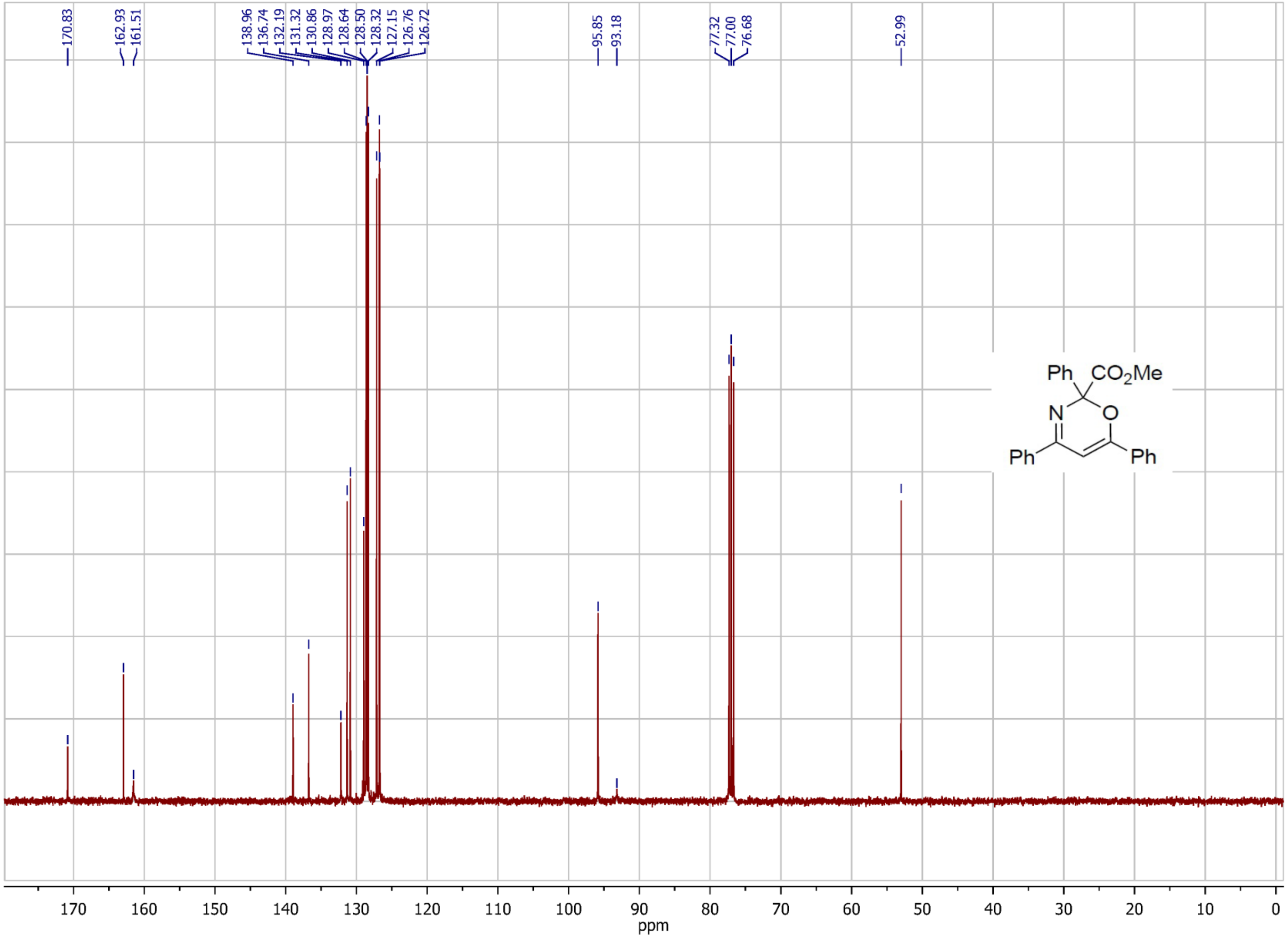

^{13}C NMR (100 MHz, $CDCl_3$, 323K) spectrum of compound **3d**.

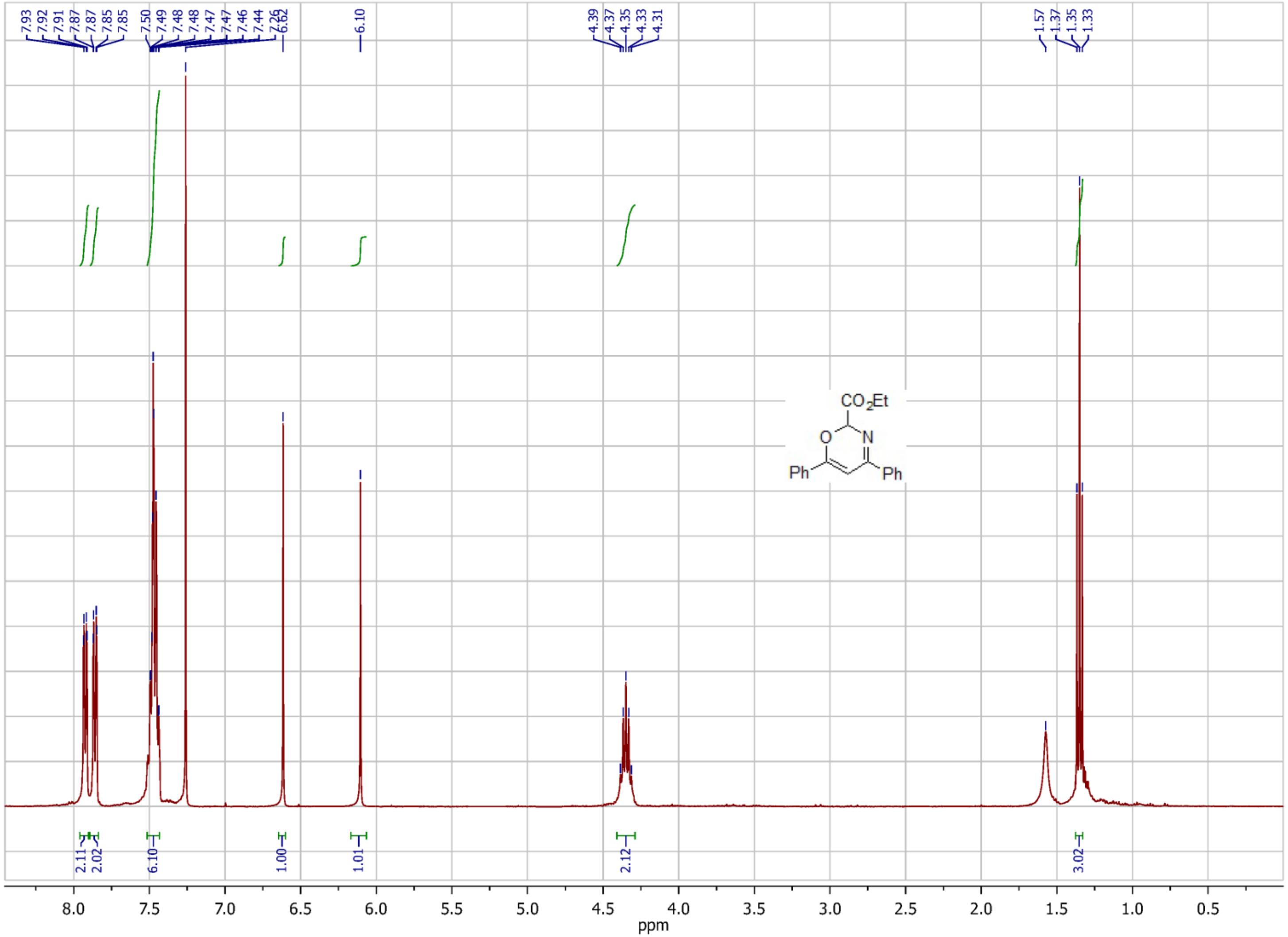

^{1}H NMR (400 MHz, $CDCl_3$) spectrum of compound **3e**.

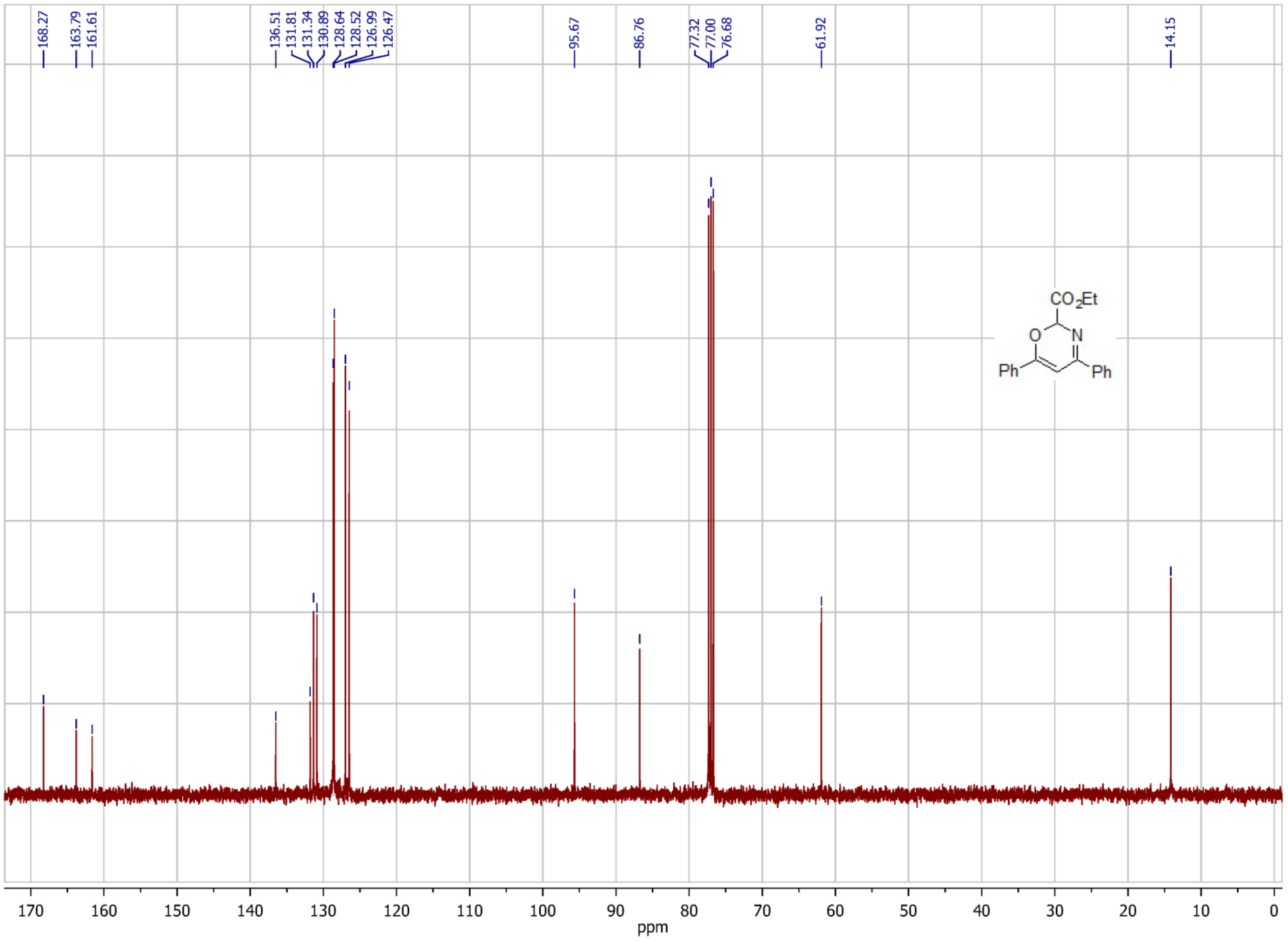

^{13}C NMR (100 MHz, $CDCl_3$) spectrum of compound **3e**.

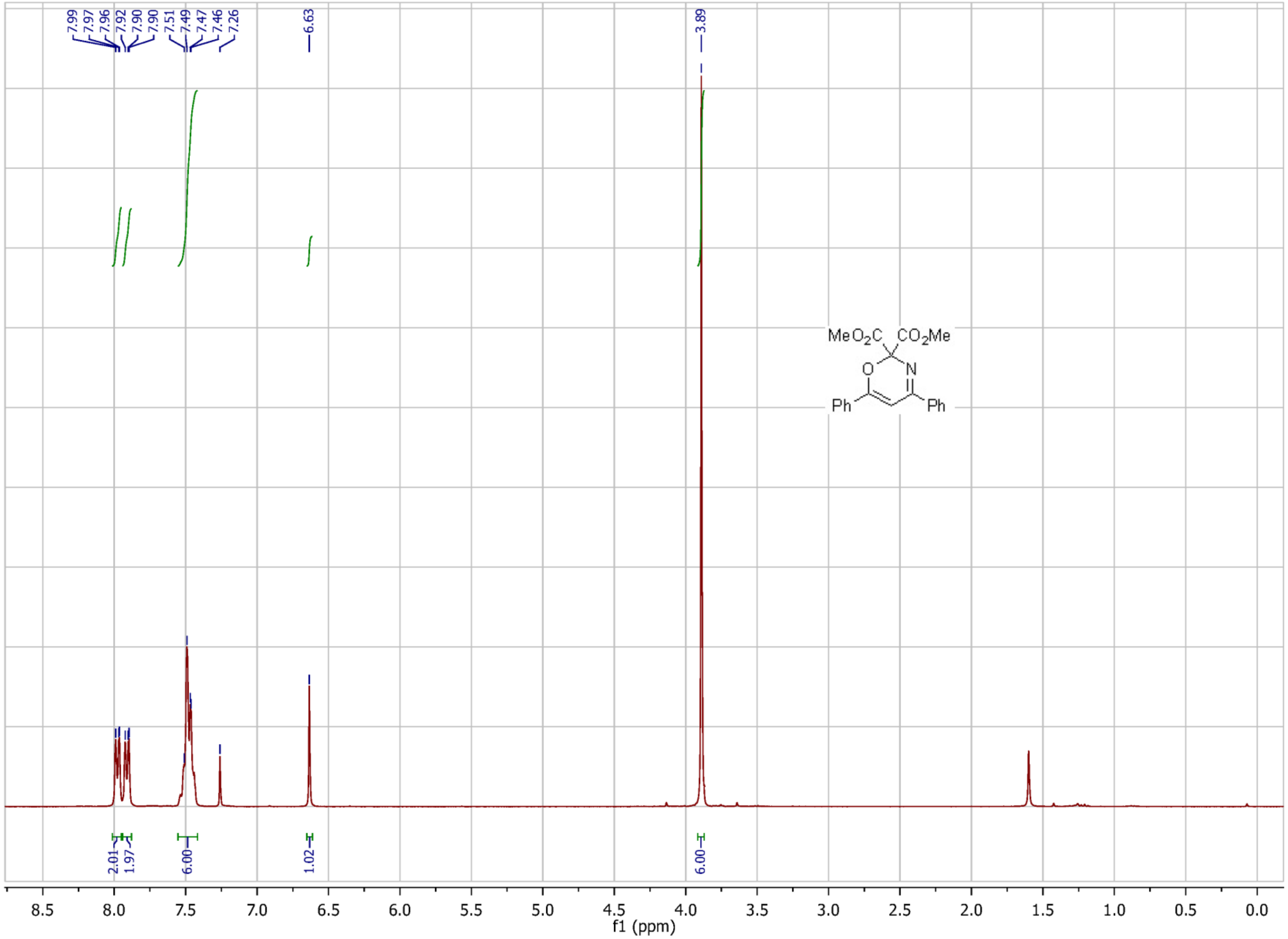

^{1}H NMR (300 MHz, $CDCl_3$) spectrum of compound **3f**.

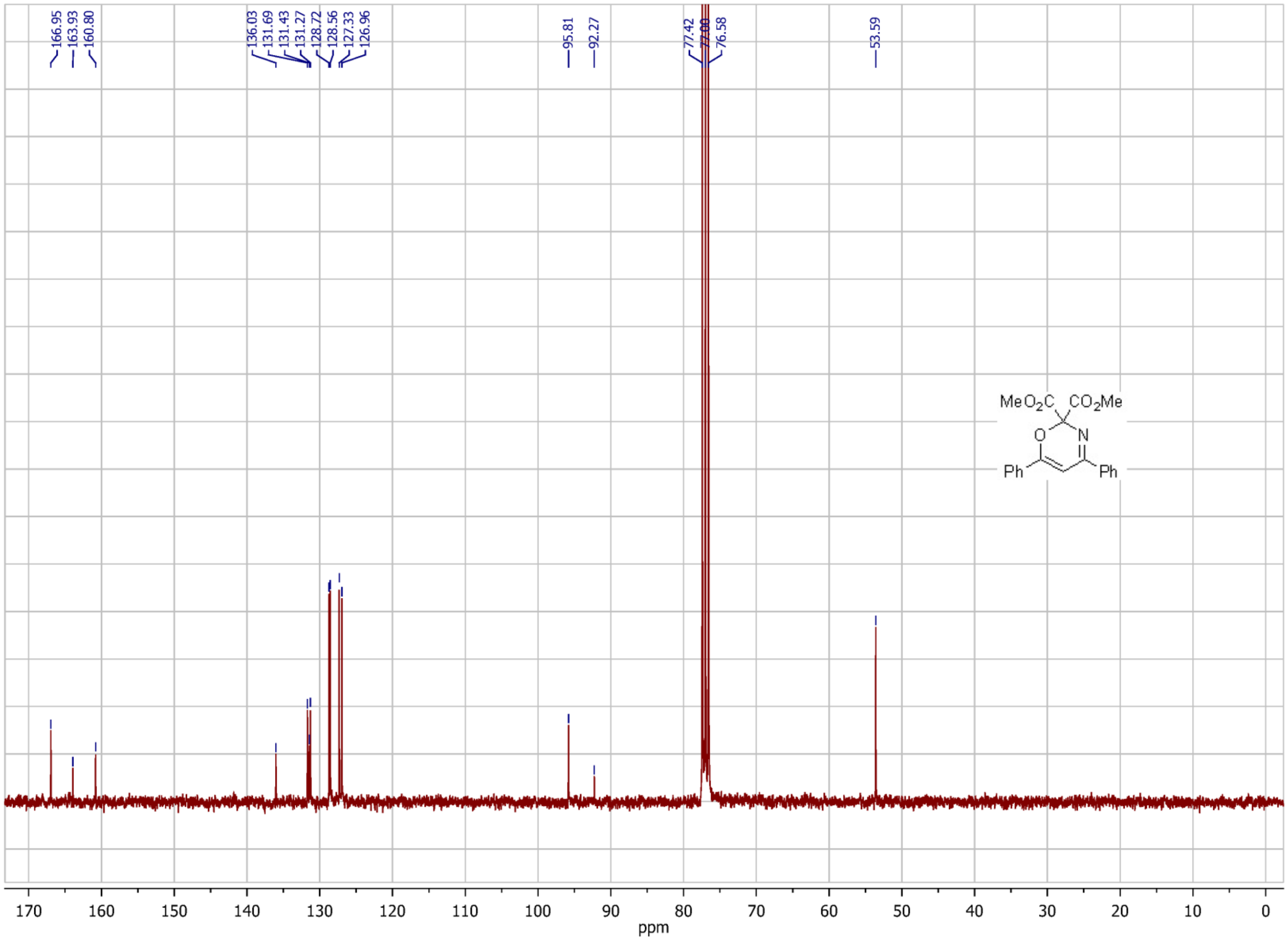

^{13}C NMR (75 MHz, $CDCl_3$) spectrum of compound **3f**.

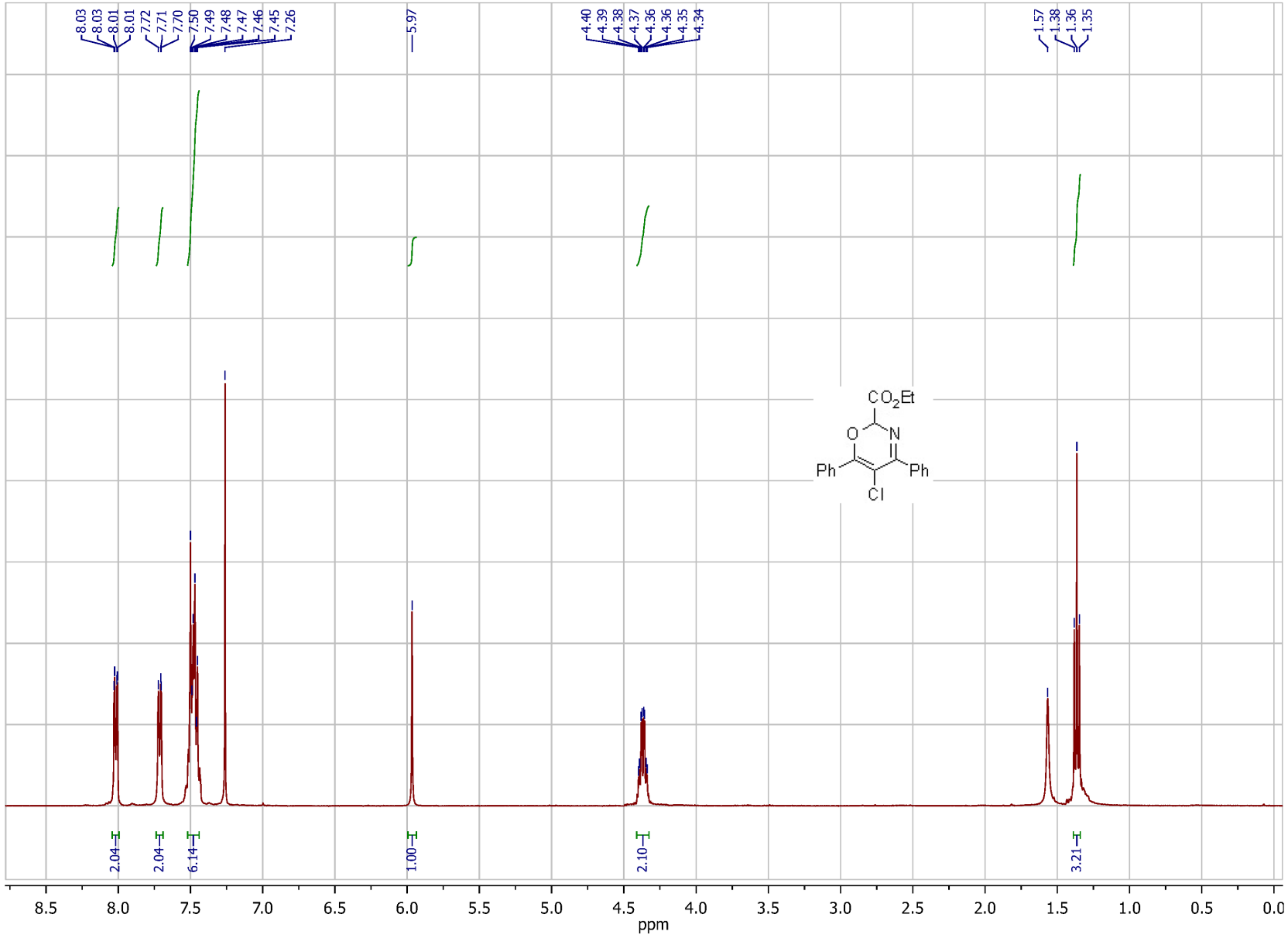

^{1}H NMR (400 MHz, $CDCl_3$) spectrum of compound **3g**.

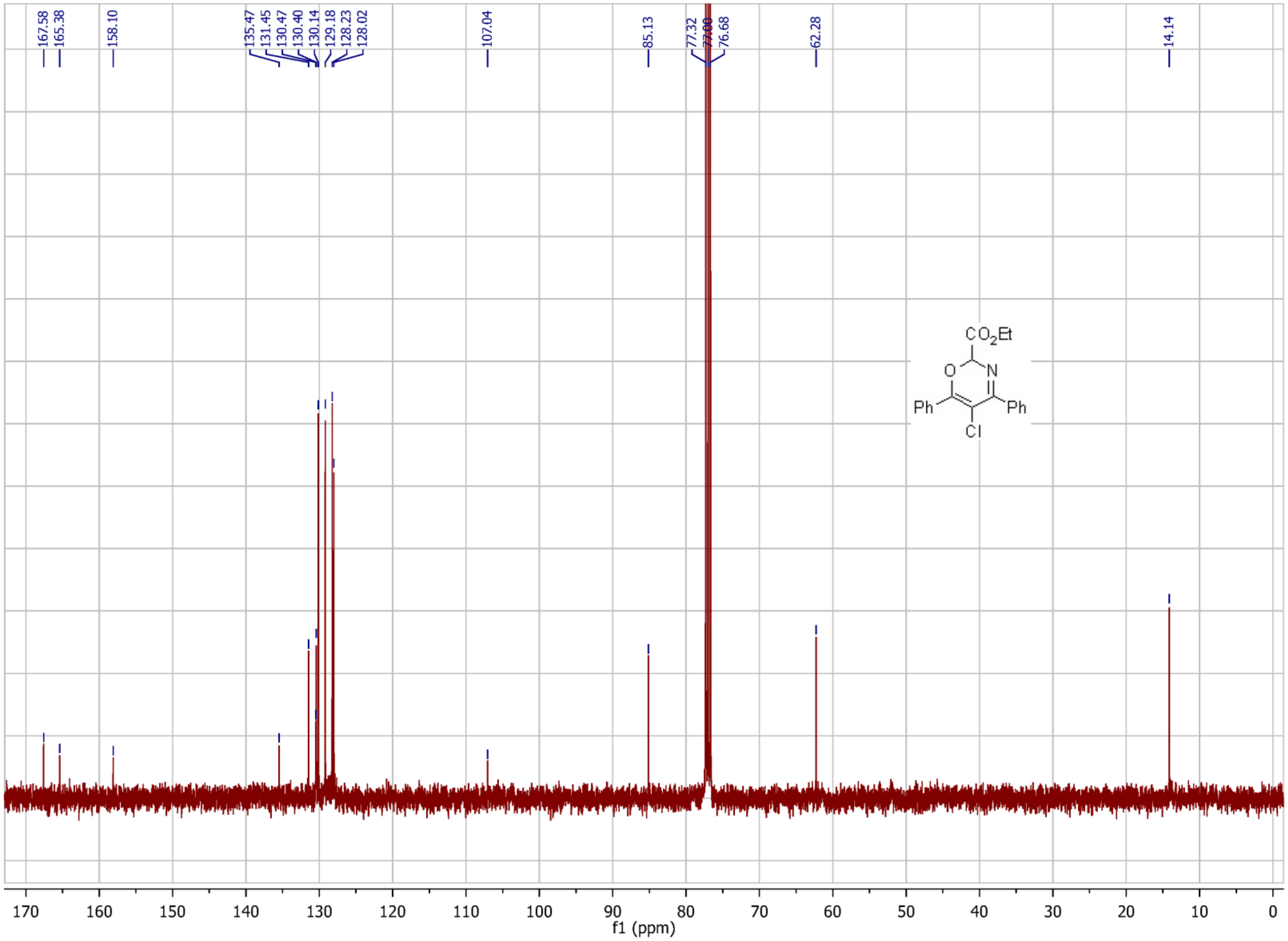

^{13}C NMR (100 MHz, $CDCl_3$) spectrum of compound **3g**.

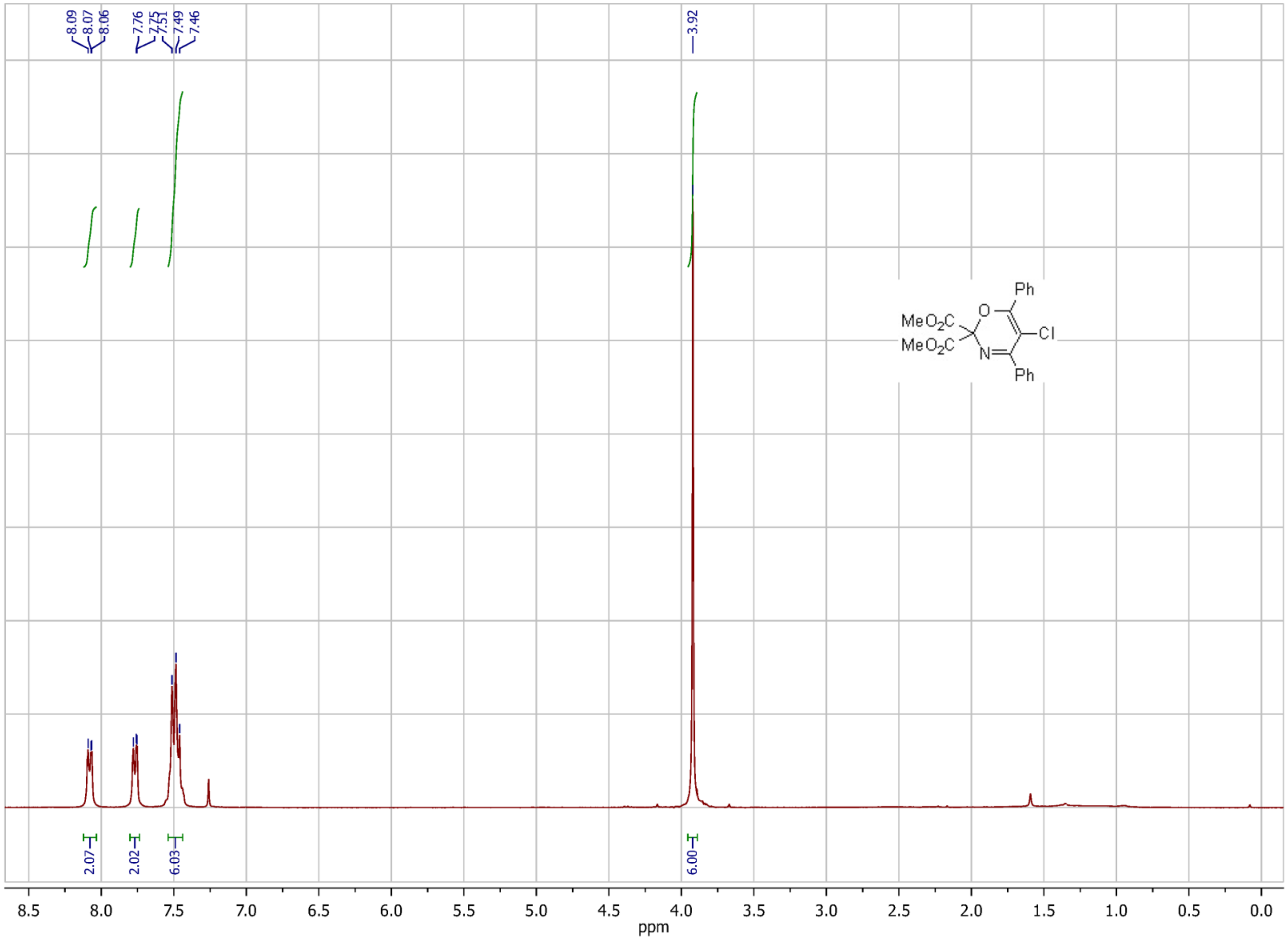

^{1}H NMR (300 MHz, $CDCl_3$) spectrum of compound **3h**.

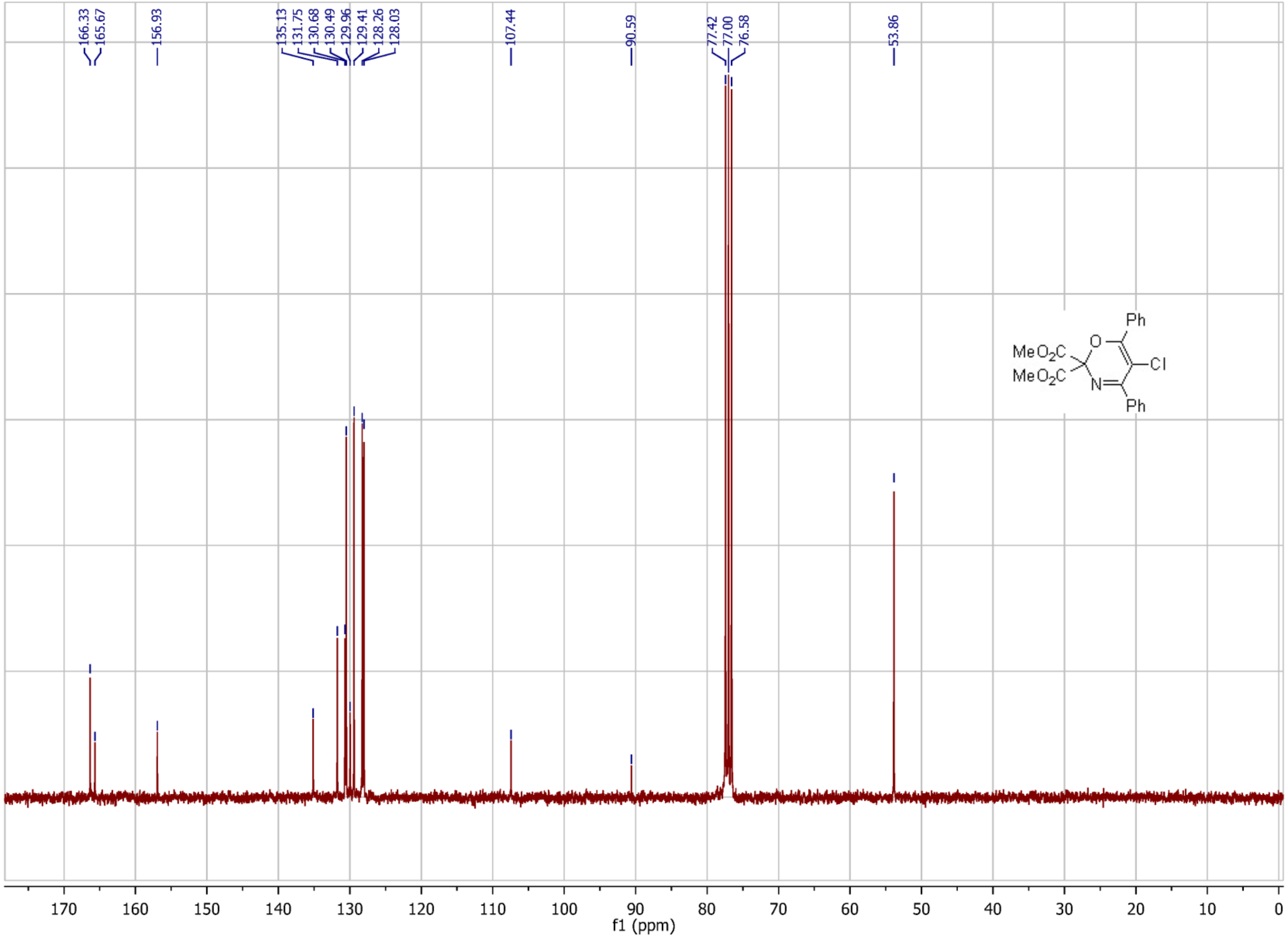

^{13}C NMR (75 MHz, $CDCl_3$) spectrum of compound **3h**.

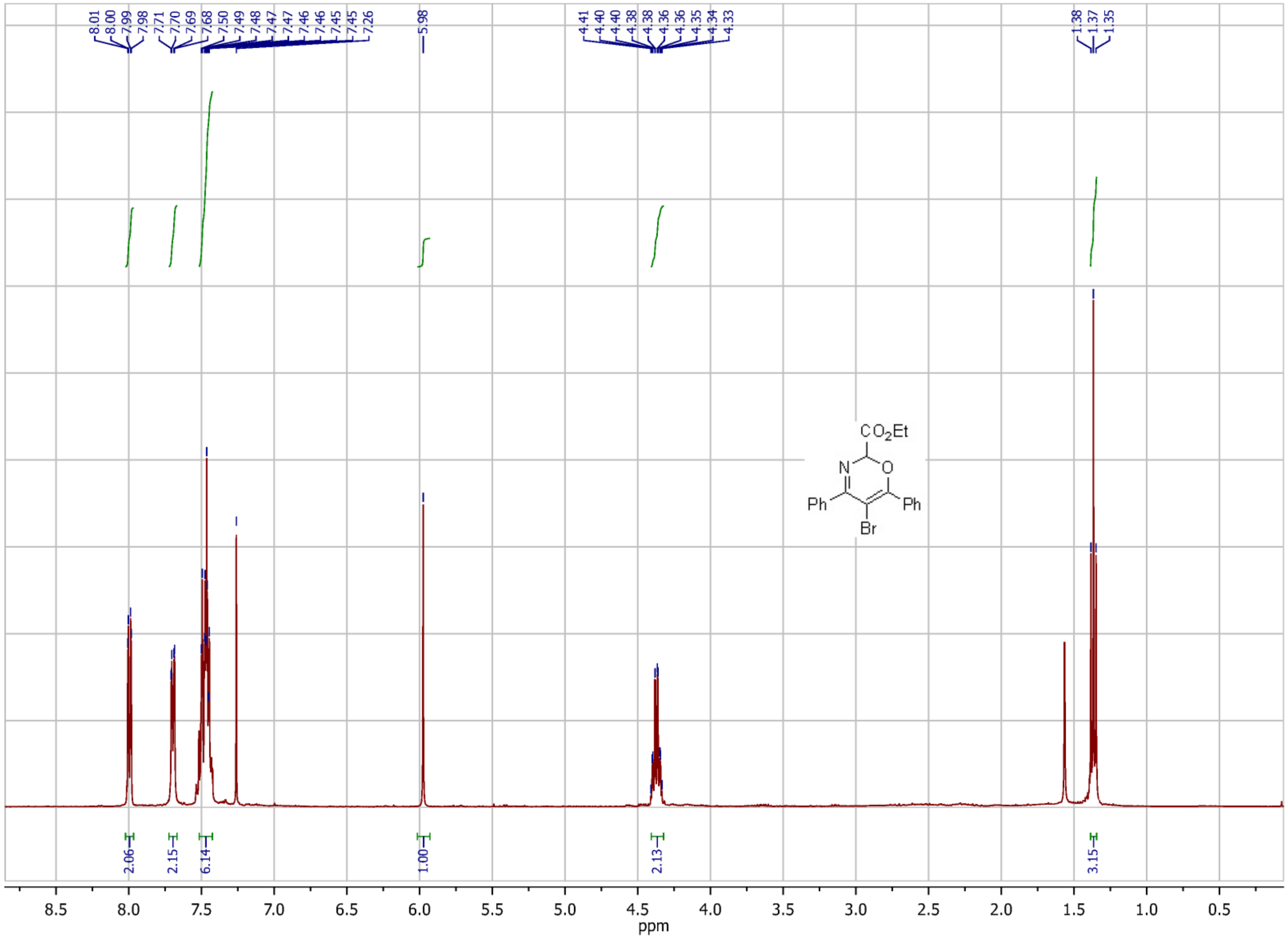

^{1}H NMR (300 MHz, $CDCl_3$) spectrum of compound **3i**.

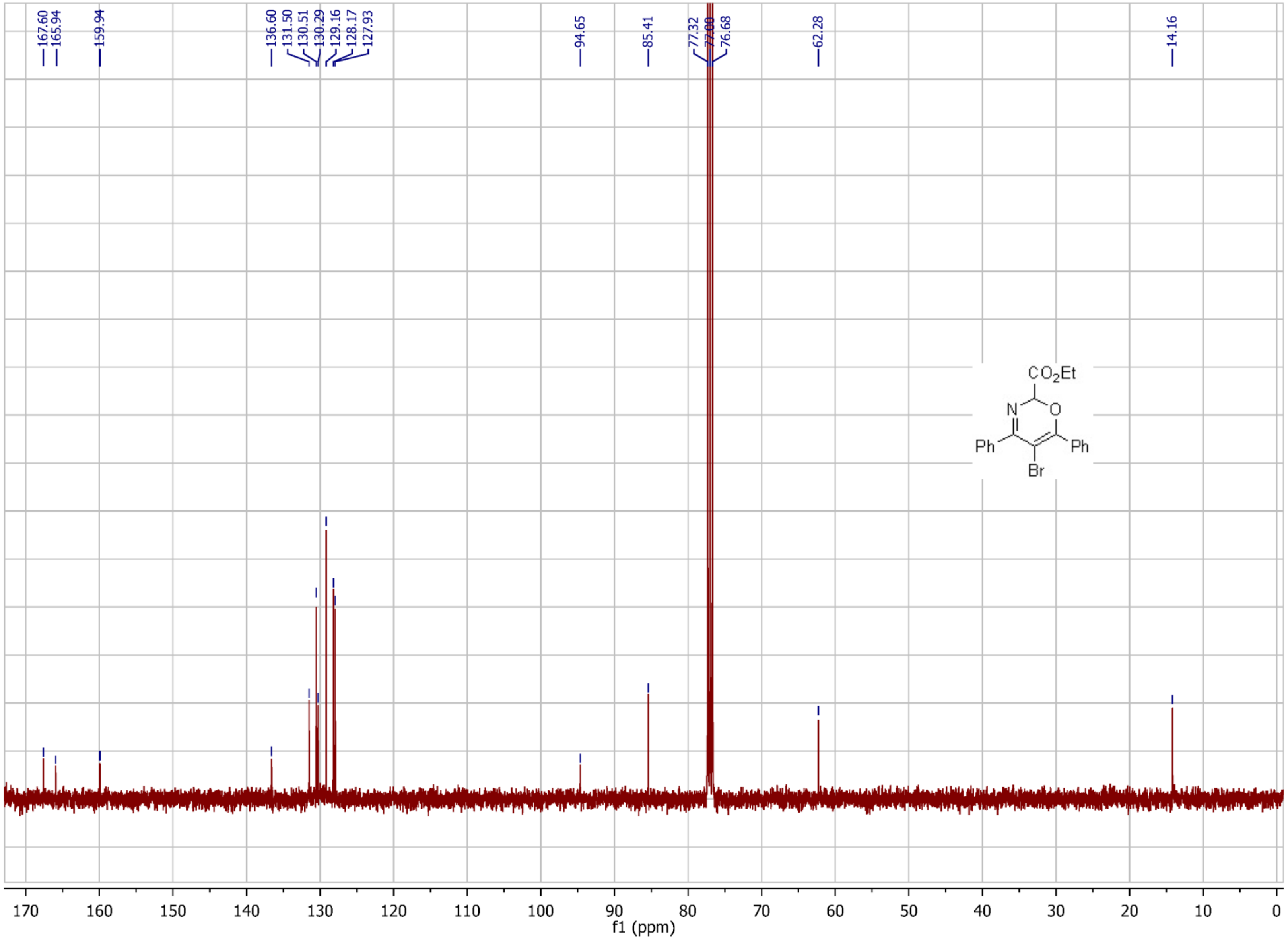

^{13}C NMR (100 MHz, $CDCl_3$) spectrum of compound **3i**.

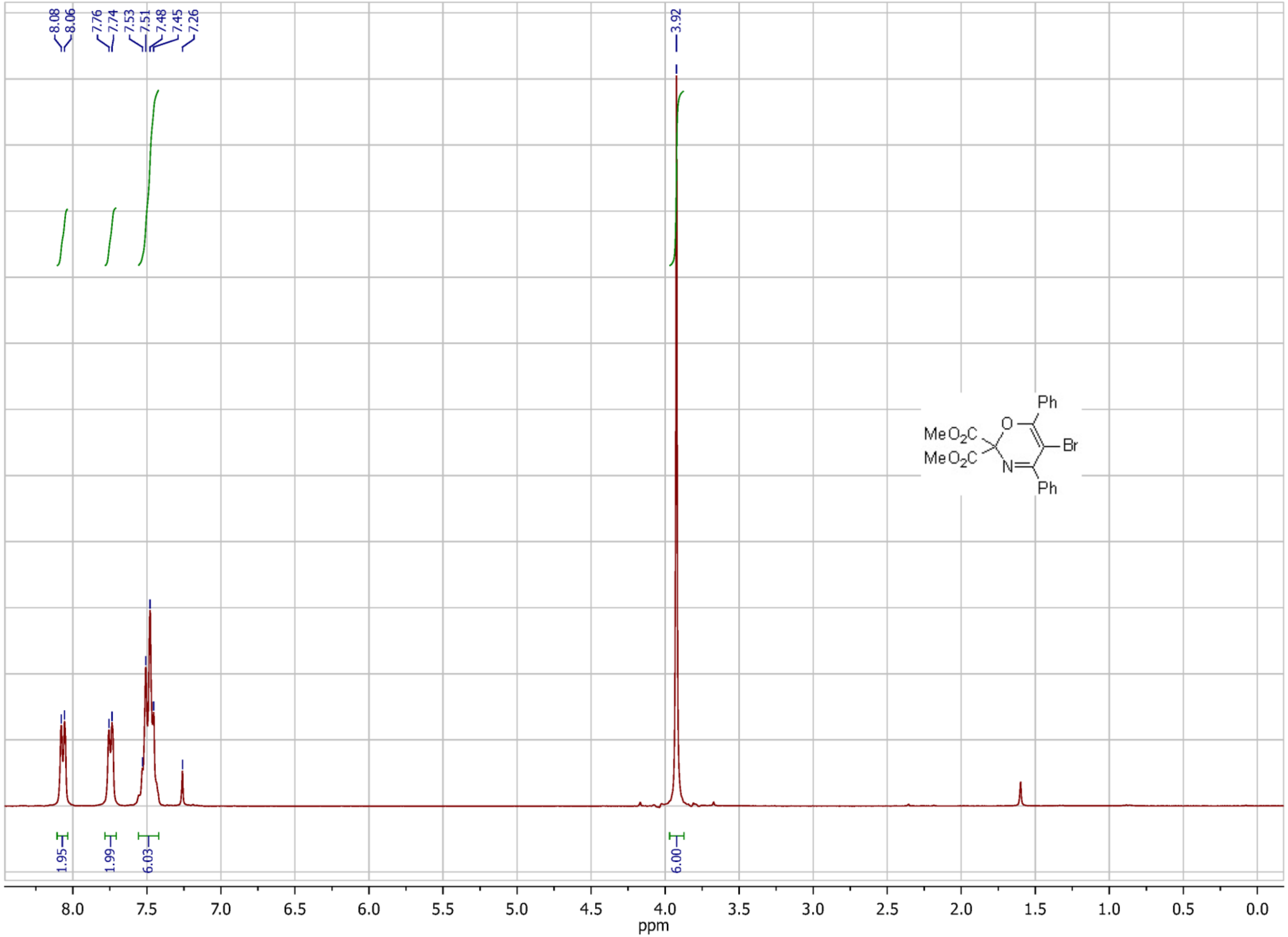

^{1}H NMR (400 MHz, $CDCl_3$) spectrum of compound **3k**.

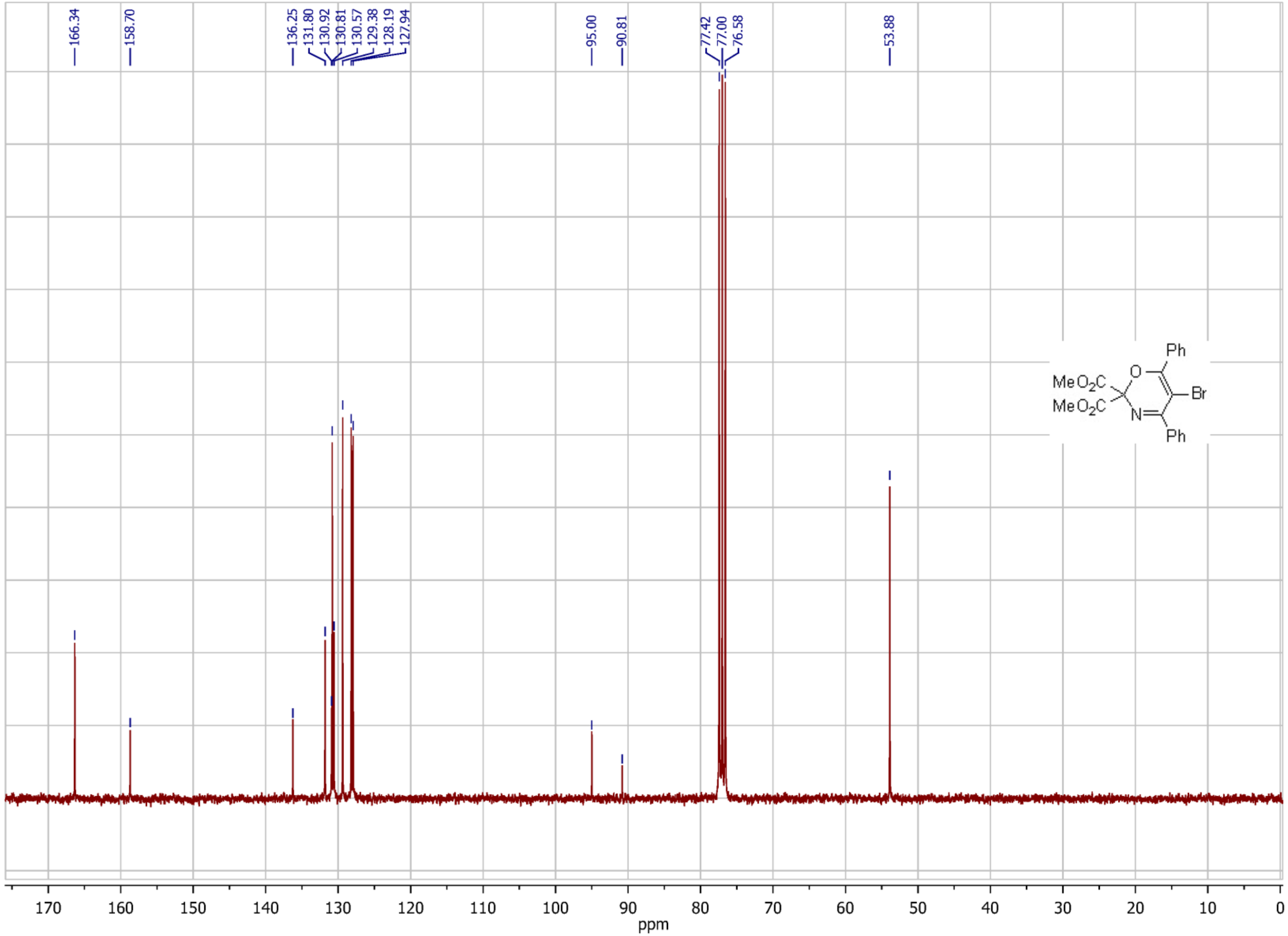

^{13}C NMR (400 MHz, $CDCl_3$) spectrum of compound **3k**.

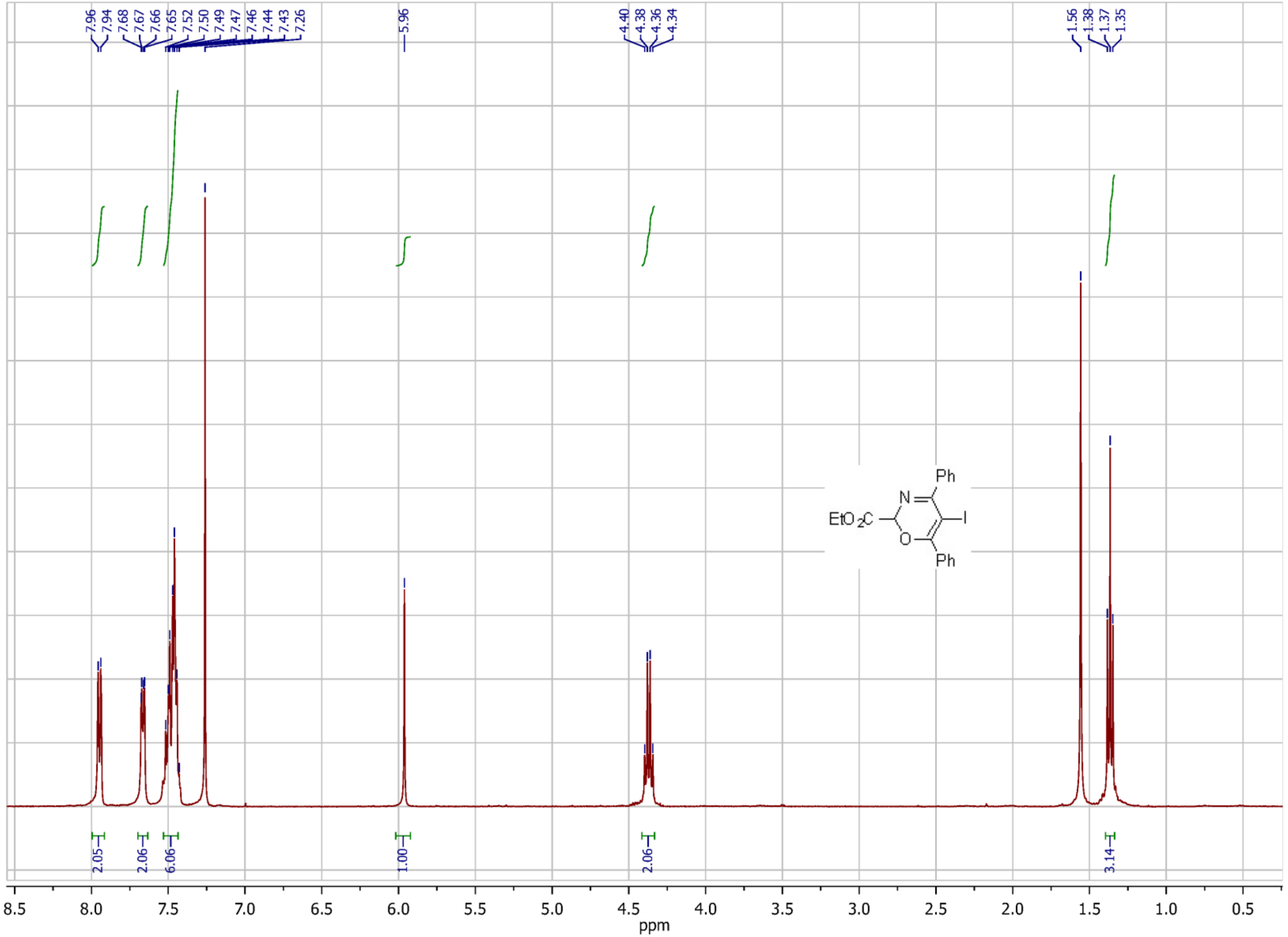

^{1}H NMR (300 MHz, $CDCl_3$) spectrum of compound **3l**.

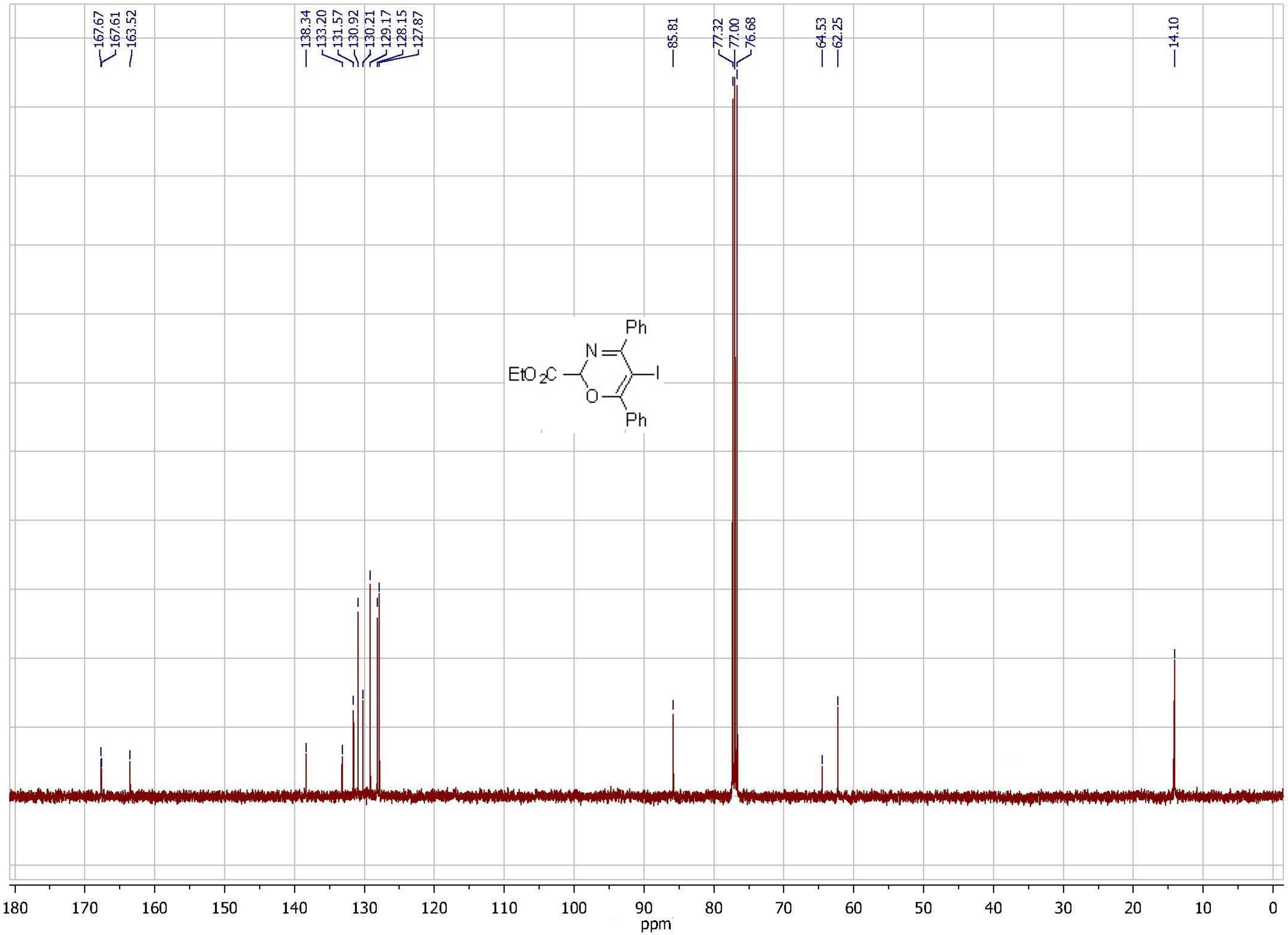

^{13}C NMR (100 MHz, $CDCl_3$) spectrum of compound **3l**.

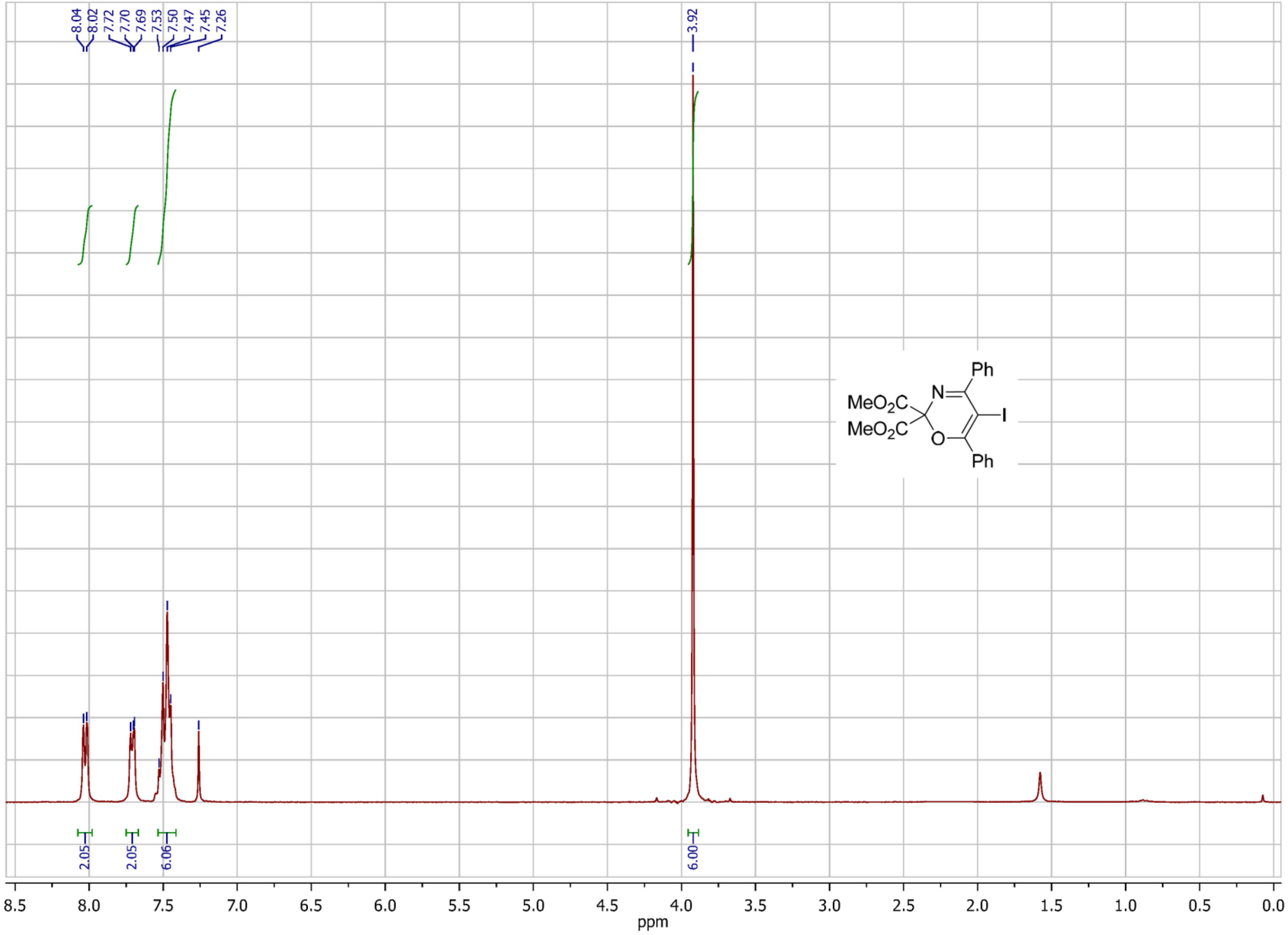

^{1}H NMR (300 MHz, $CDCl_3$) spectrum of compound **3m**.

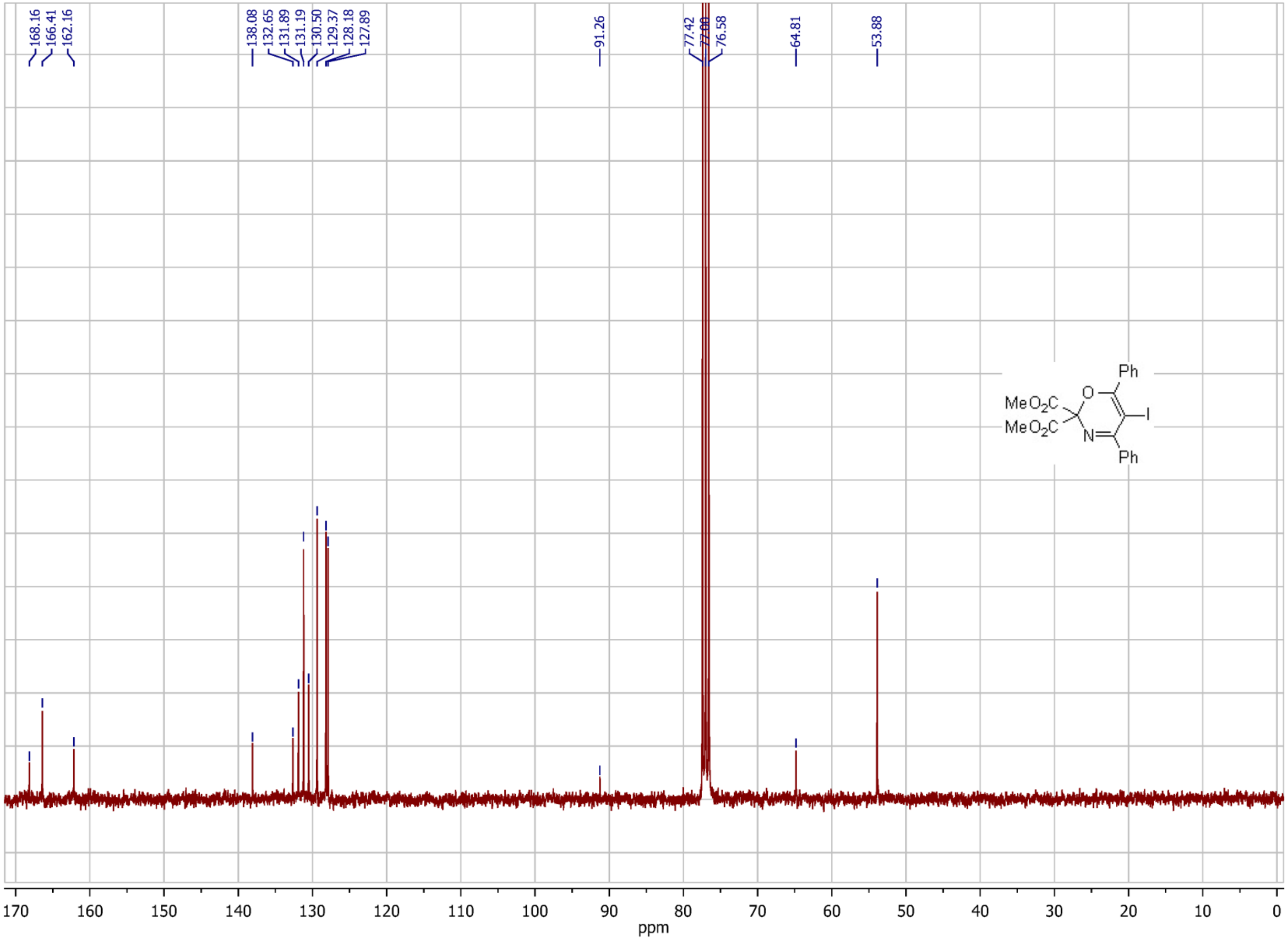

^{13}C NMR (75 MHz, $CDCl_3$) spectrum of compound **3m**.

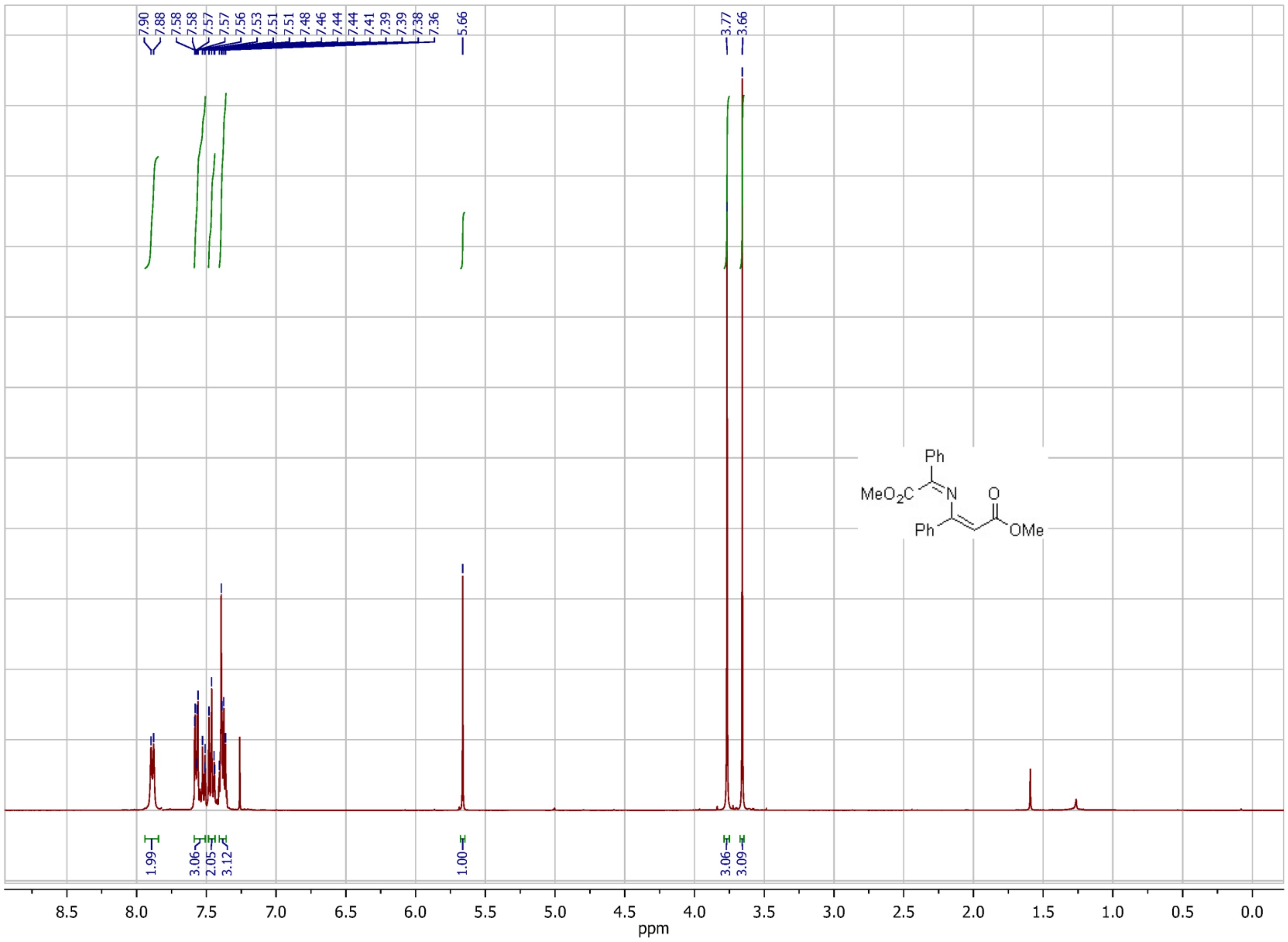

^{1}H NMR (400 MHz, $CDCl_3$) spectrum of compound **4a**.

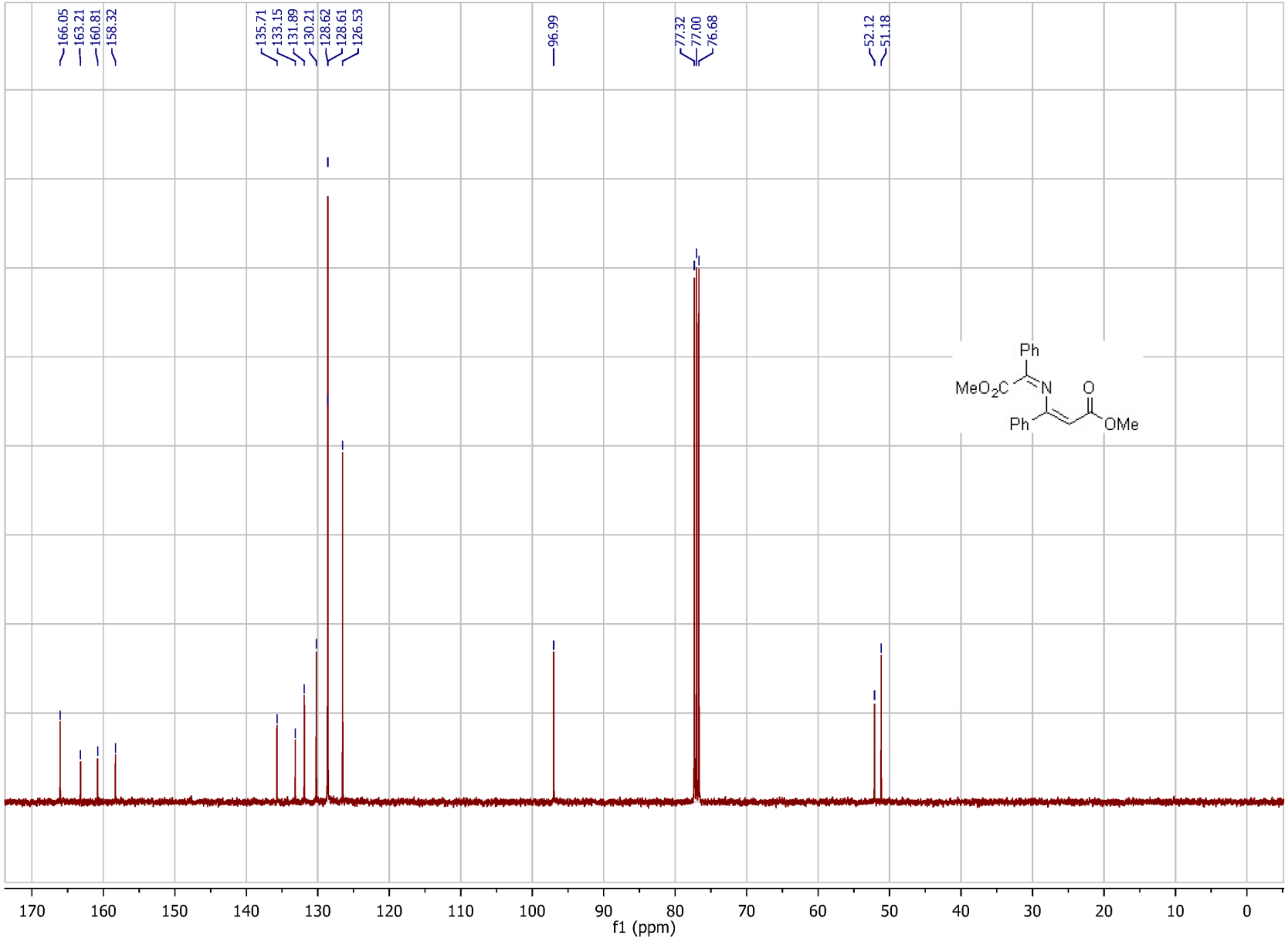

^{13}C NMR (100 MHz, $CDCl_3$) spectrum of compound **4a**.

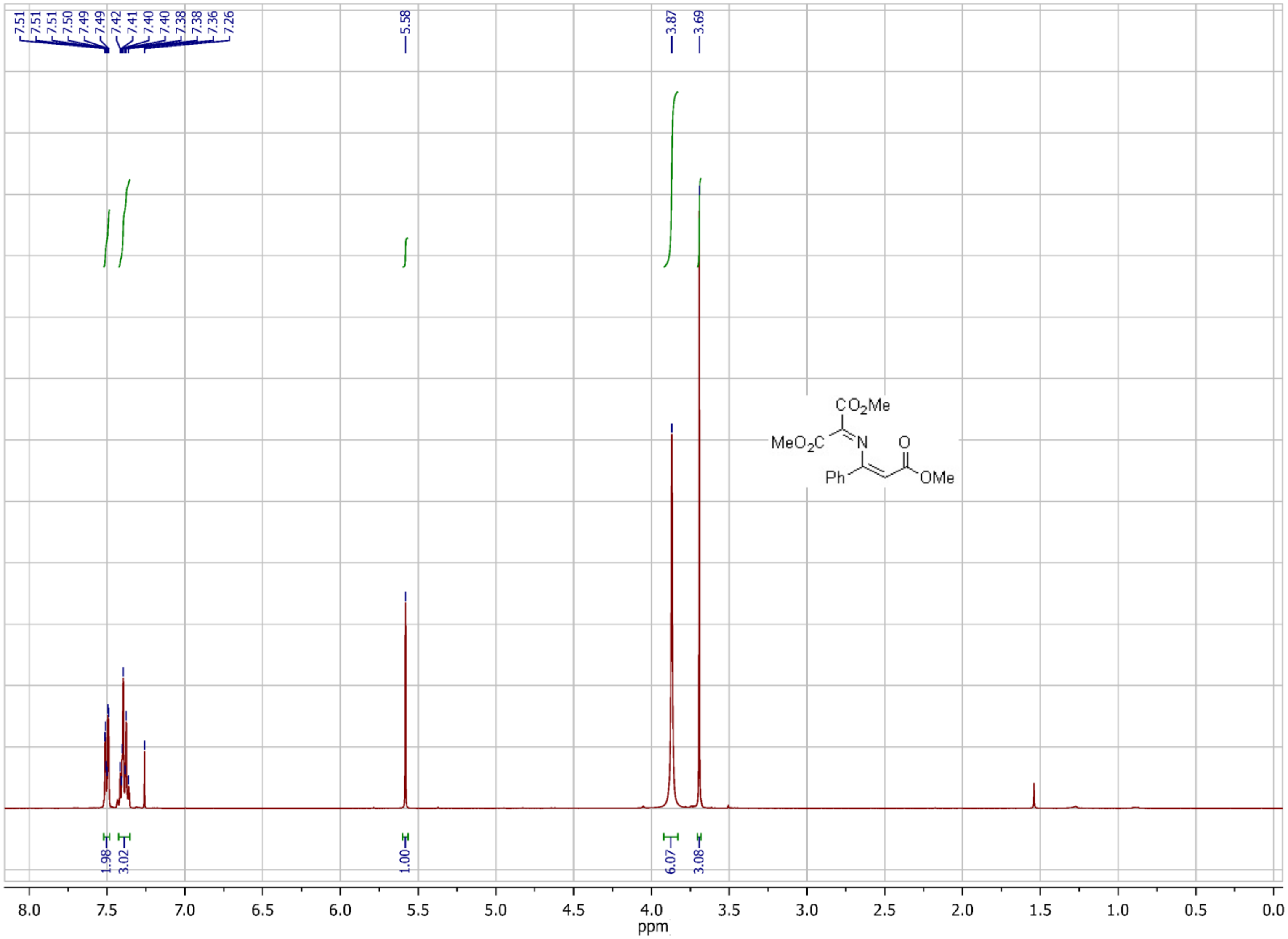

^{1}H NMR (400 MHz, $CDCl_3$) spectrum of compound **4b**.

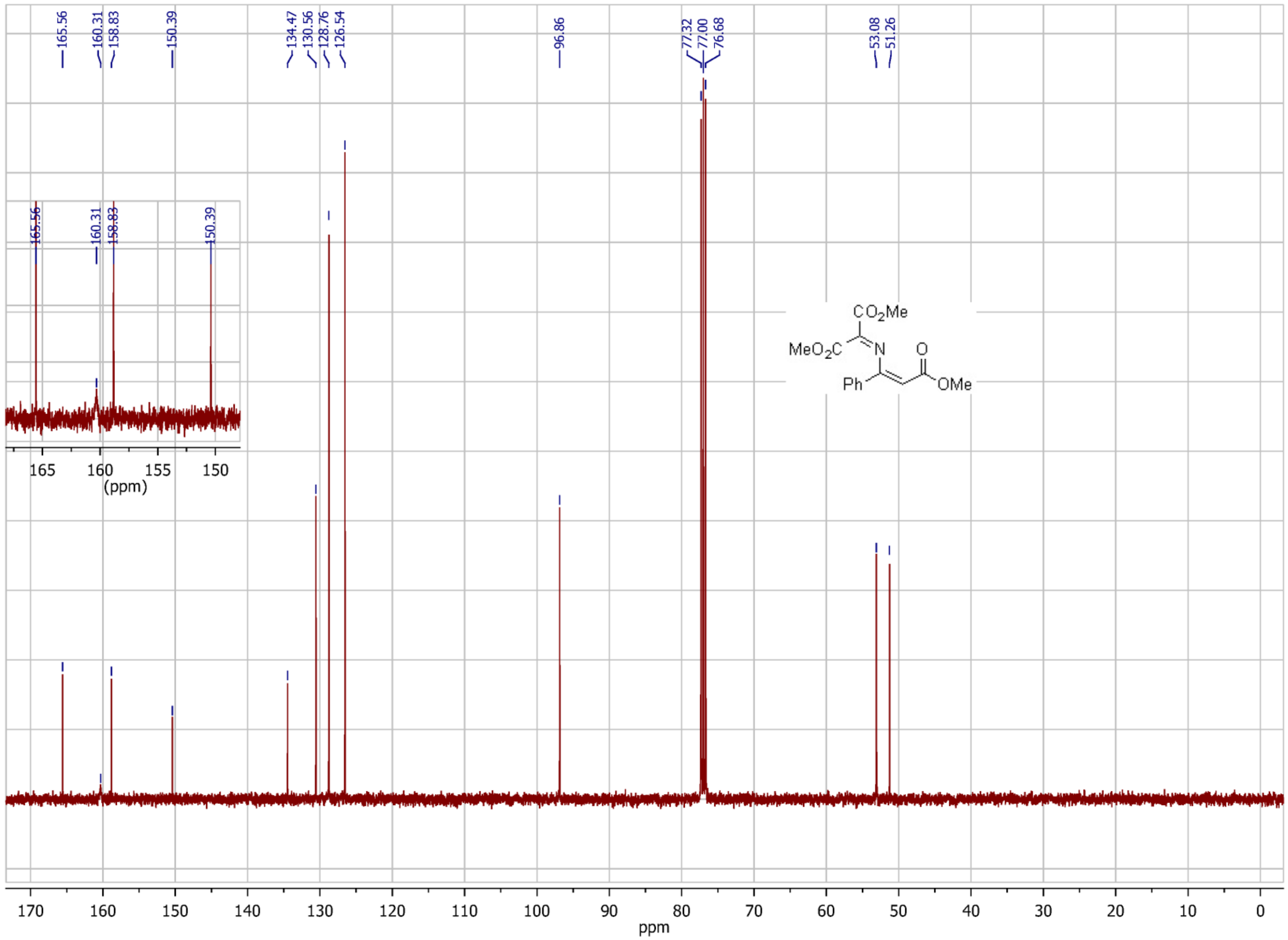

^{13}C NMR (100 MHz, 323K, $CDCl_3$) spectrum of compound **4b**.

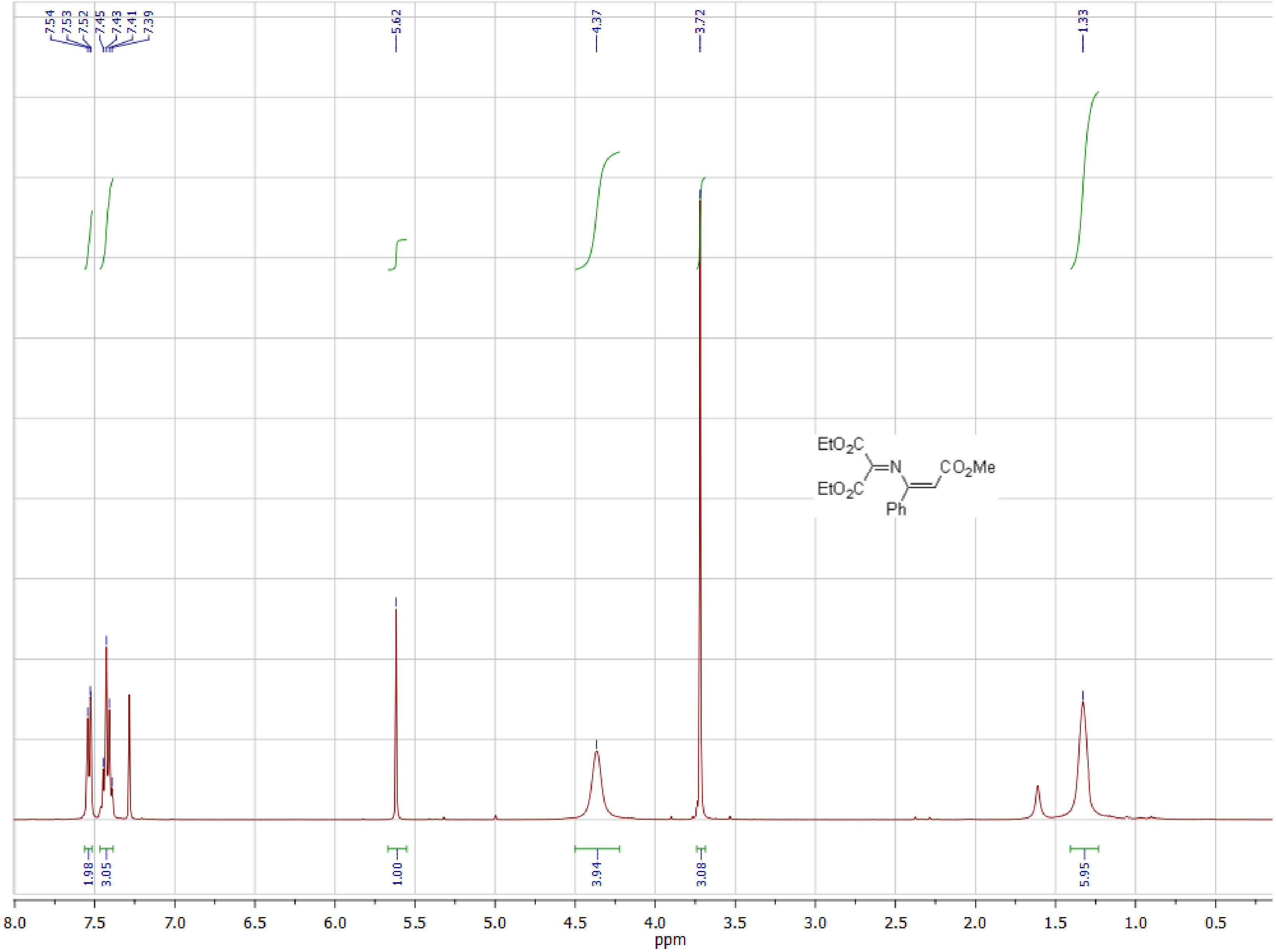

^{1}H NMR (400 MHz, $CDCl_3$) spectrum of compound **4c**.

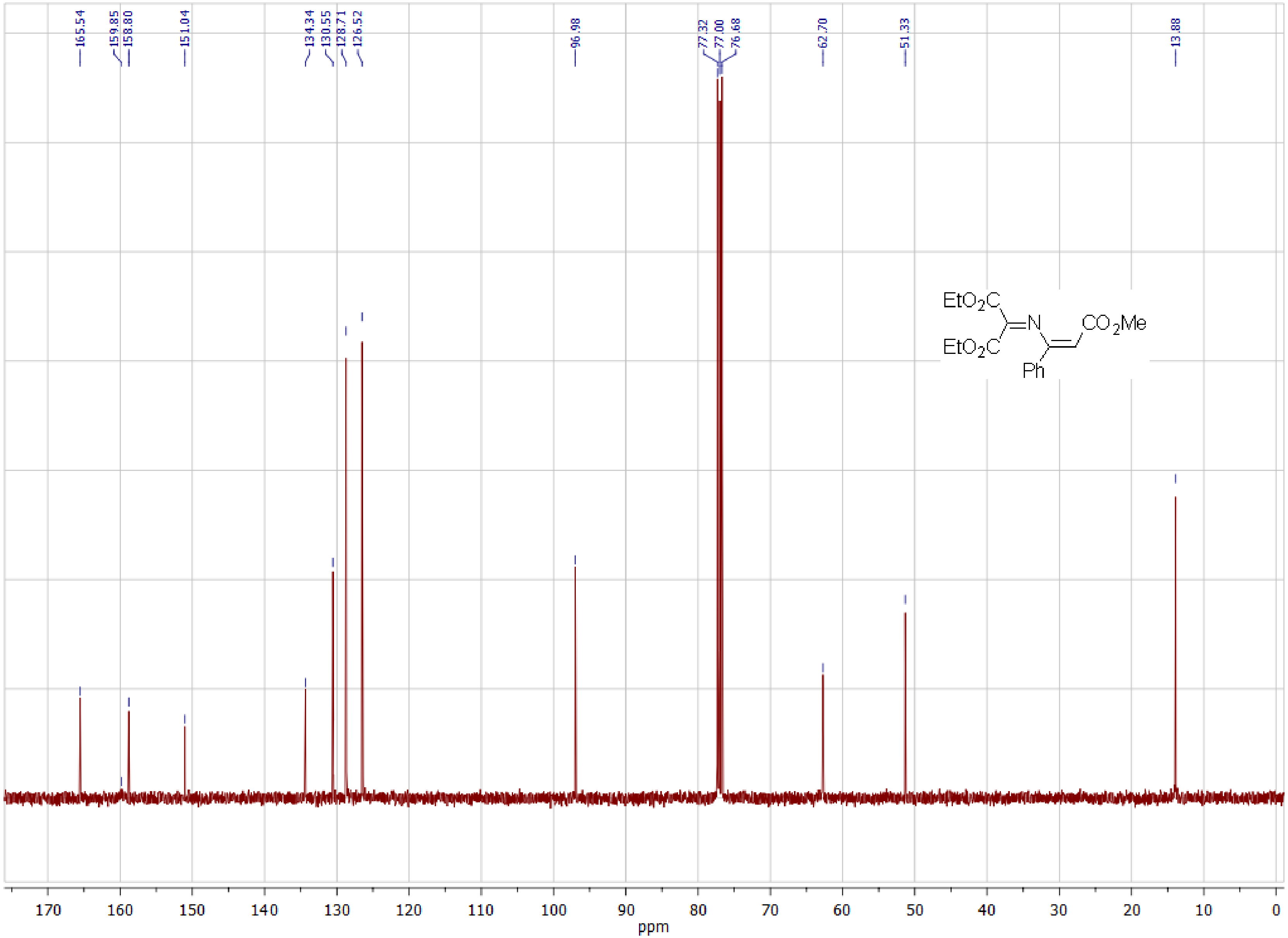

^{13}C NMR (100 MHz, $CDCl_3$) spectrum of compound **4c**.

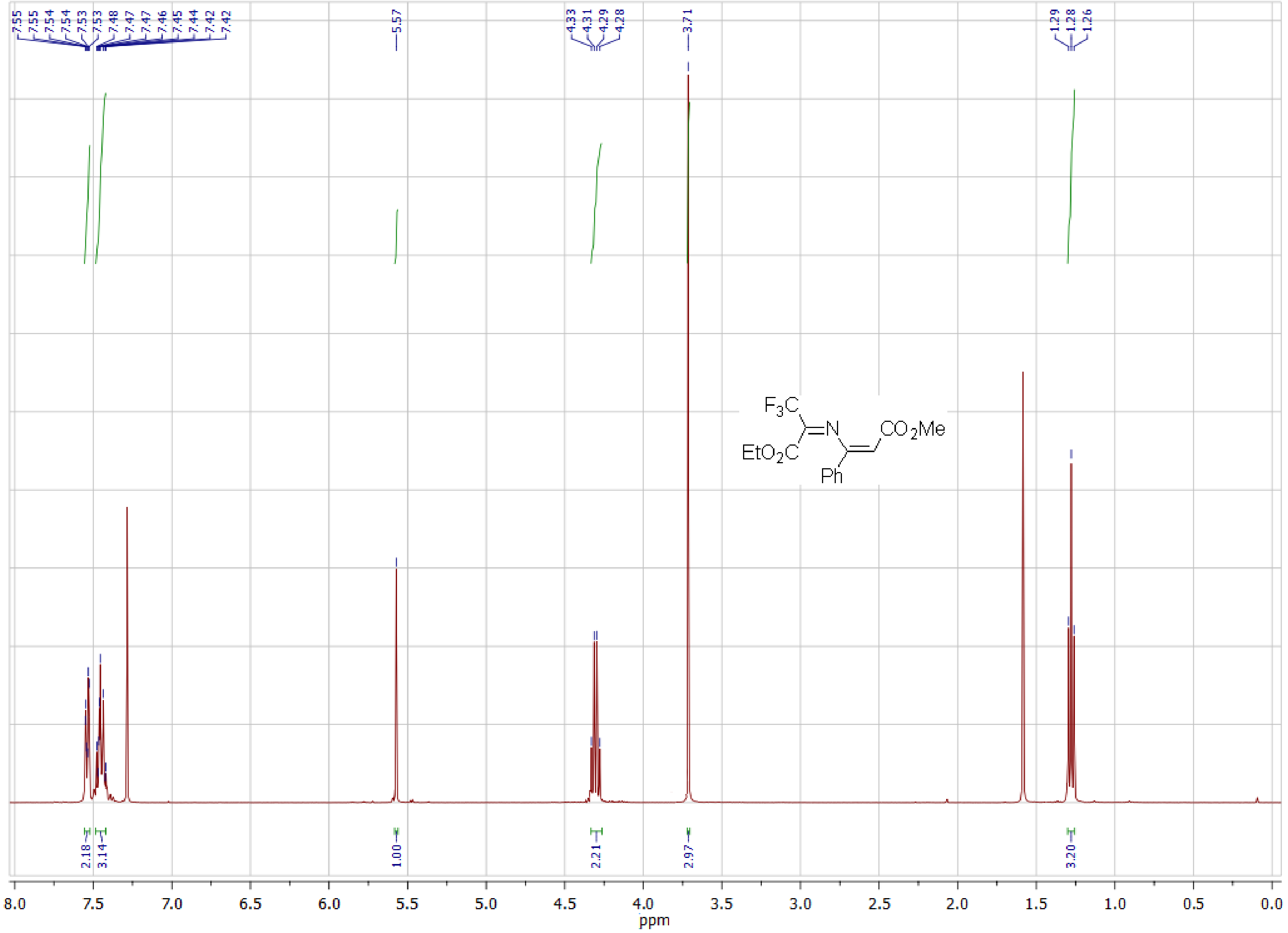

^{1}H NMR (400 MHz, $CDCl_3$) spectrum of compound **4d**.

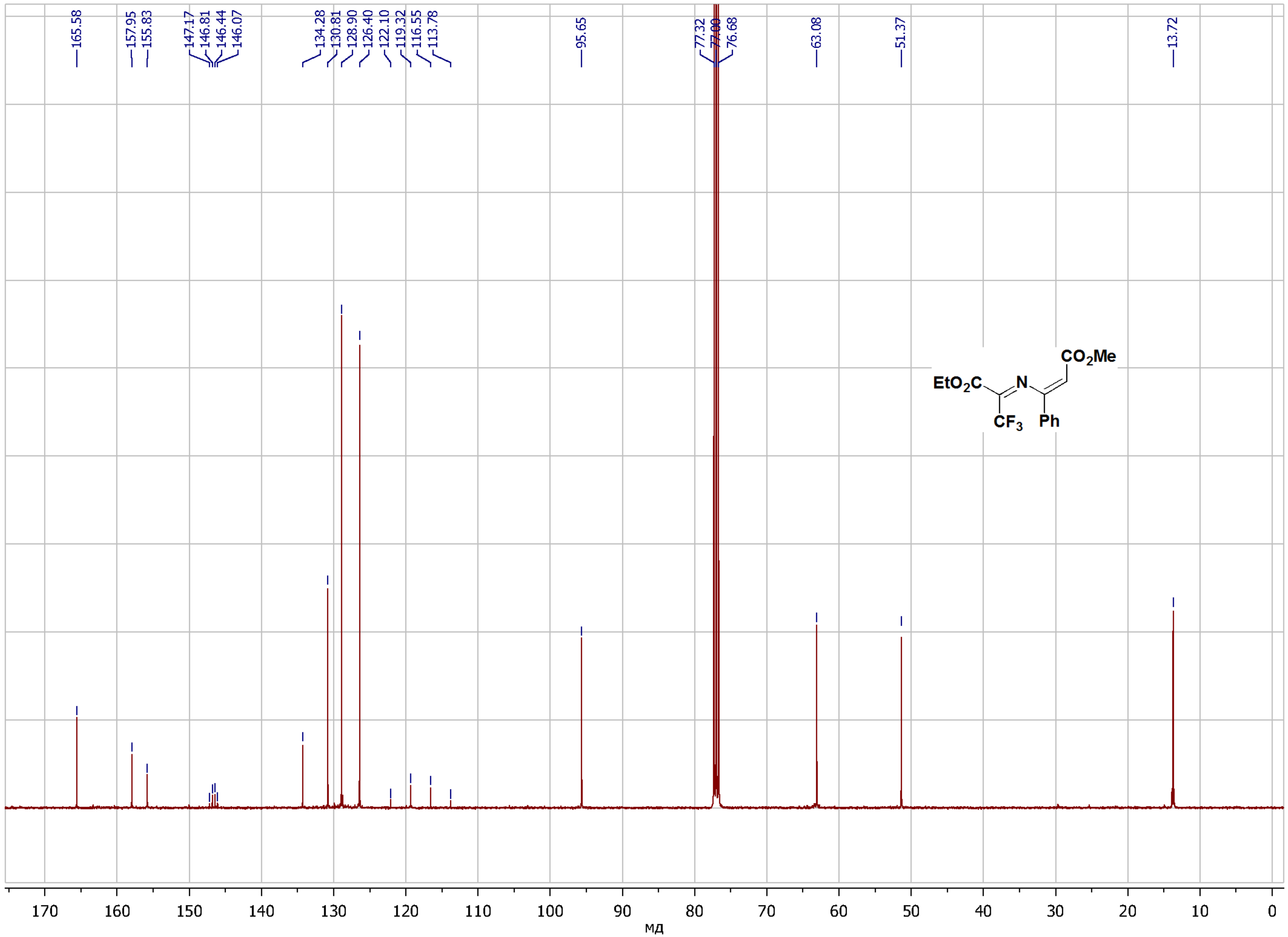

^{13}C NMR (100 MHz, $CDCl_3$) spectrum of compound **4d**.

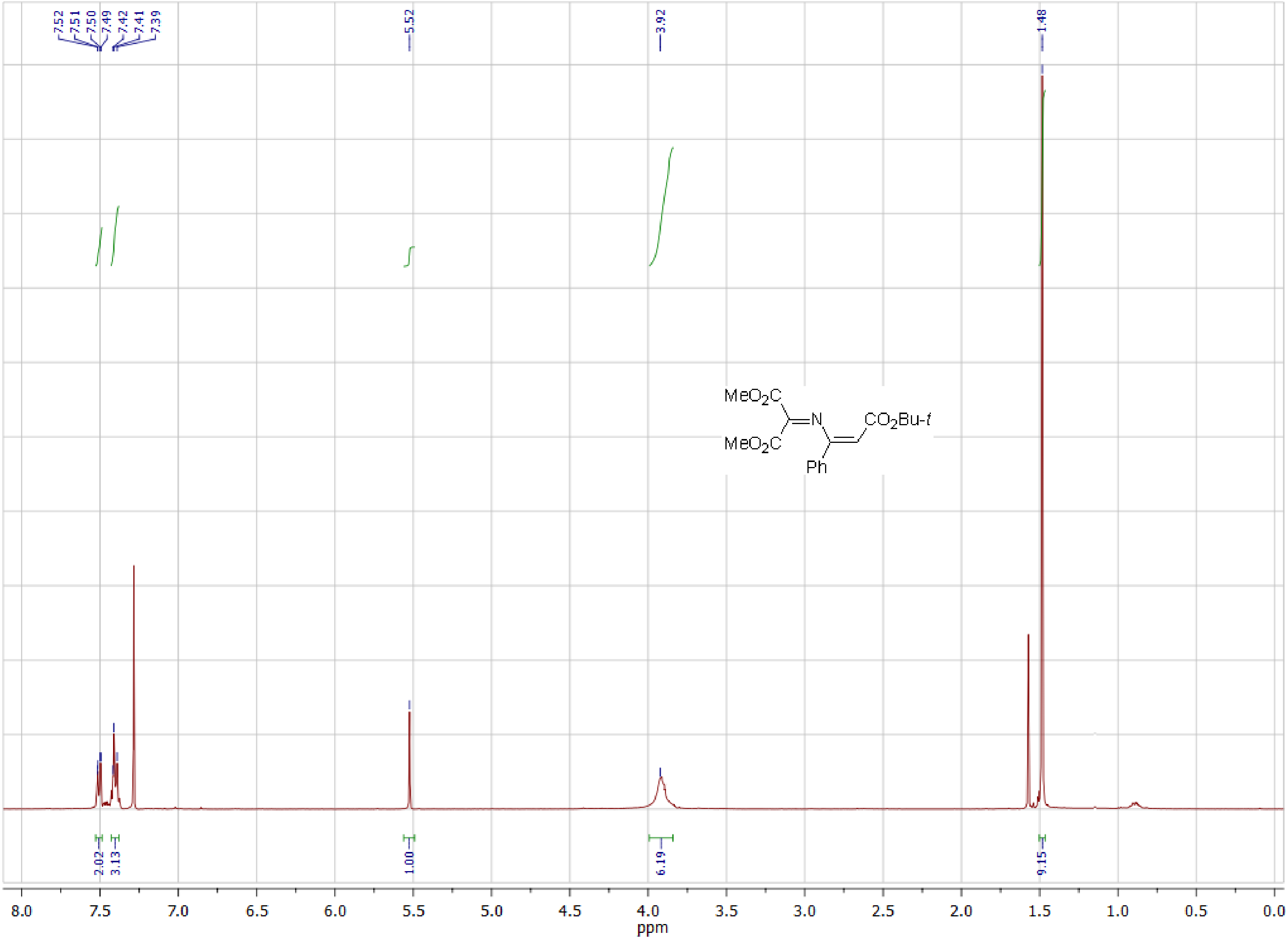

^{1}H NMR (400 MHz, $CDCl_3$) spectrum of compound **4e**.

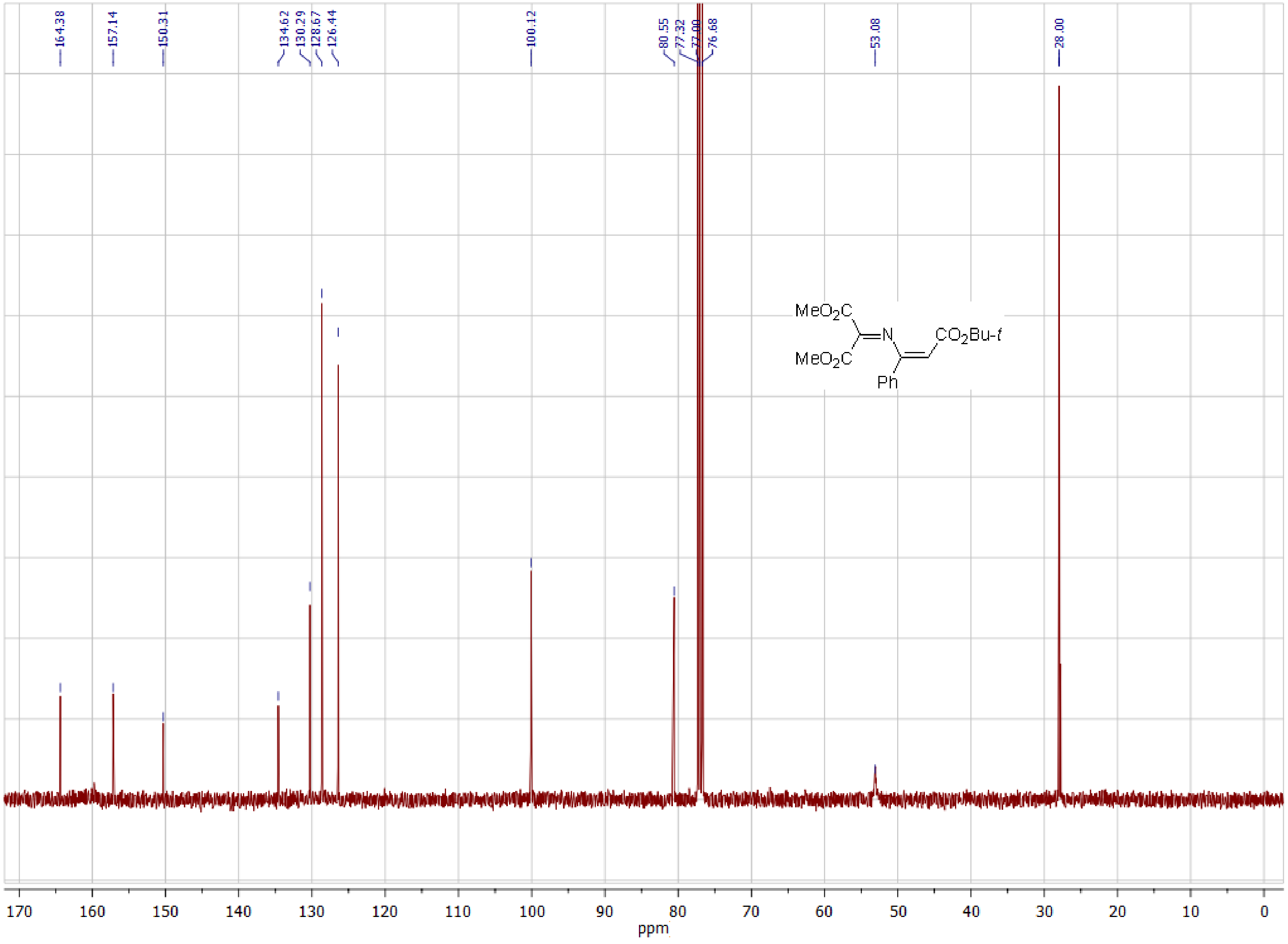

^{13}C NMR (100 MHz, $CDCl_3$) spectrum of compound **4e**.

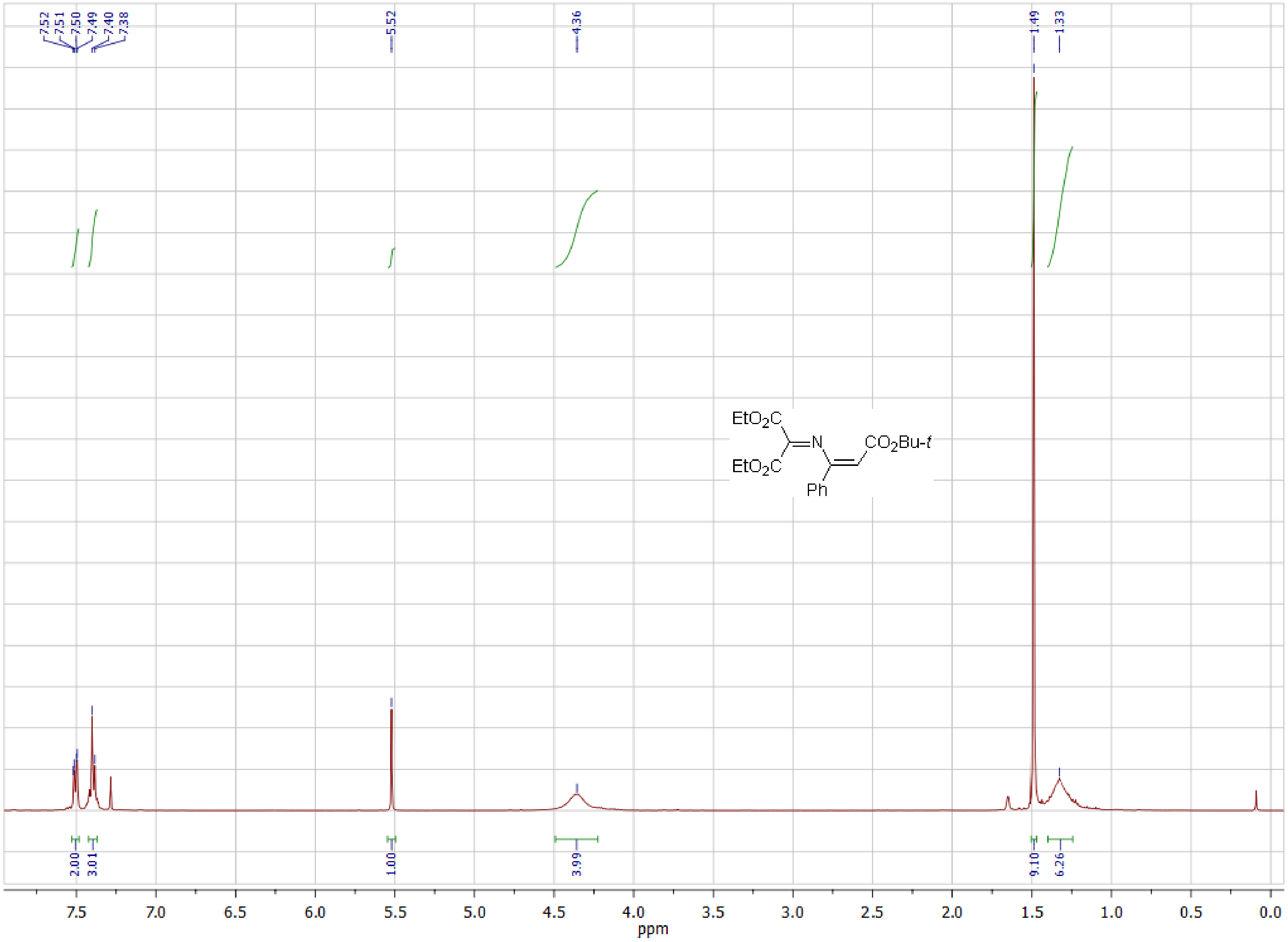

^{1}H NMR (400 MHz, $CDCl_3$) spectrum of compound **4f**.

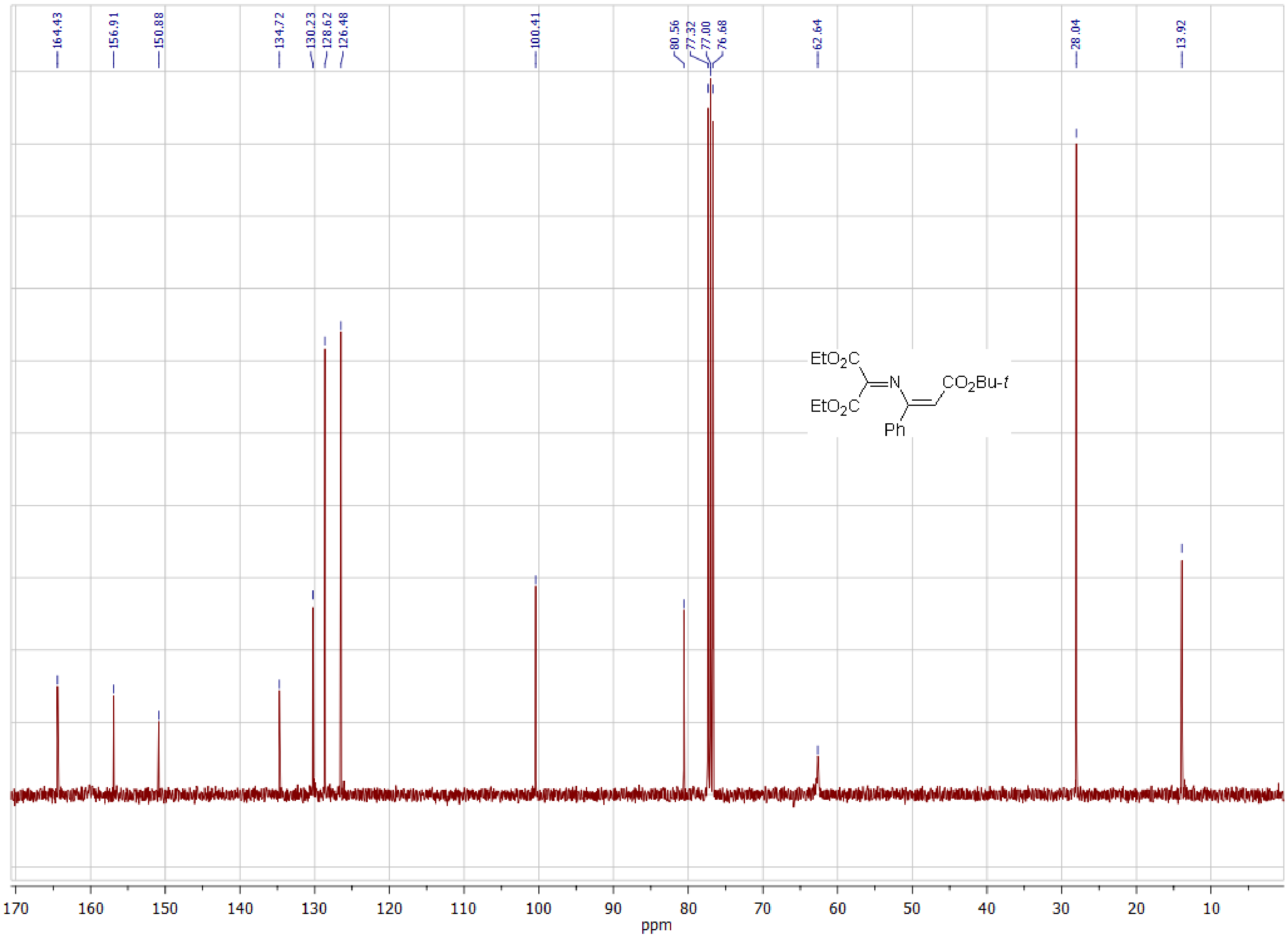

^{13}C NMR (400 MHz, $CDCl_3$) spectrum of compound **4f**.

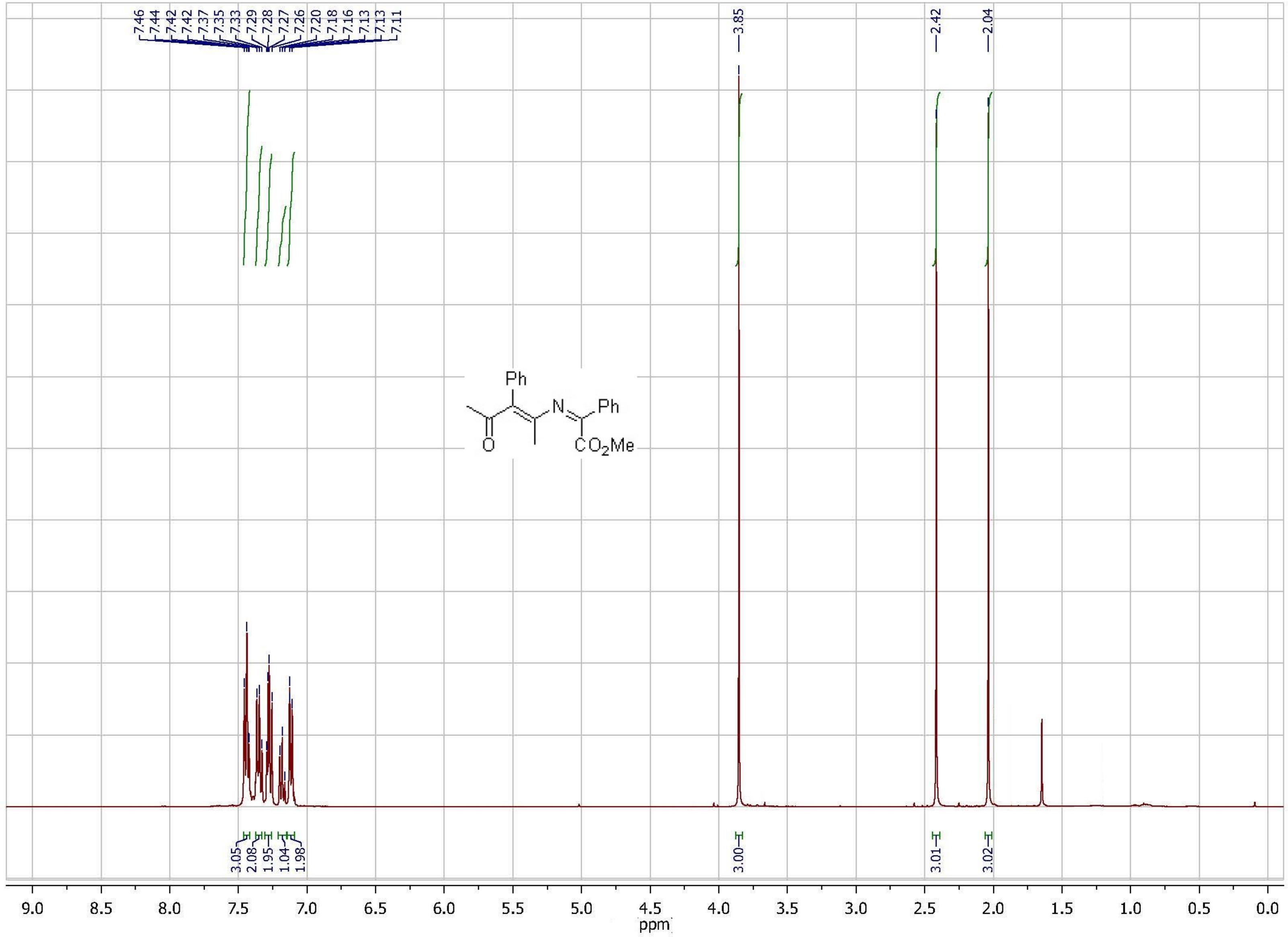

^{1}H NMR (400 MHz, $CDCl_3$) spectrum of compound (*E*)-**4i**.

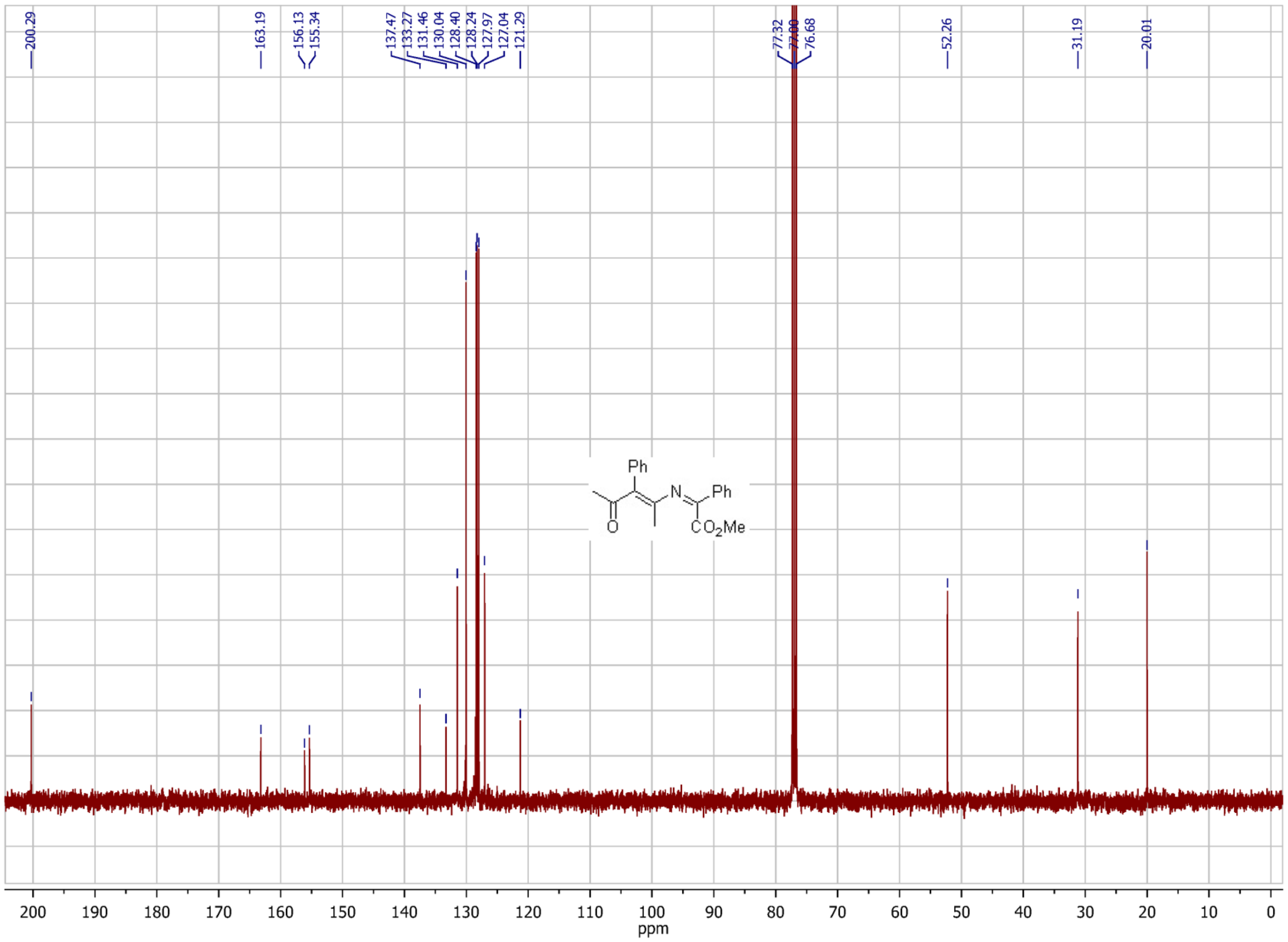

^{13}C NMR (00 MHz, $CDCl_3$) spectrum of compound (*E*)-**4i**.

Computational Details

All calculations were performed with the B3LYP density functional method[1] by using the Gaussian 09 suite of quantum chemical programs[2] at Resource center "Computer center of Saint Petersburg State University". Geometry optimizations of molecules were performed at the B3LYP/6-31G(d) level in *vacuuo* or with pcm solvent model for CH_2Cl_2.

Table. B3LYP/6-31G(d) Absolute Energies (au), Cartesian Coordinates of stationary points

Fig.1. Molecule **C** (R^1=R^2=R^3=H)
(PCM for CH_2Cl_2)

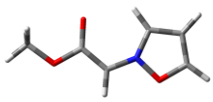

E = -513.17520209, **H (0K)** = -513.046961,
H (298K) = -513.036616, **G (298K)** = -513.082177 au.
Imaginary frequency = 0.

`C	1.4646260	1.0067430	-0.0014110
N	0.9865150	-0.2438090	-0.0007710
O	2.0413460	-1.1351350	0.0007000
C	3.1908570	-0.3811120	0.0008170
C	2.8811760	0.9372870	-0.0001440
C	-0.2628720	-0.8139340	-0.0009500
C	-1.3999980	0.0230980	0.0000500
O	-1.4102770	1.2635230	0.0006710
O	-2.5603770	-0.7139420	0.0001850
C	-3.7680310	0.0519670	0.0006410
H	4.1009720	-0.9586510	0.0012890
H	3.5629620	1.7737710	-0.0002630
H	-0.2886330	-1.8909660	-0.0016840
H	-4.5788820	-0.6783810	0.0004260
H	-3.8355830	0.6856630	0.8903890
H	-3.8357660	0.6864100	-0.8885630
H	0.7692390	1.8289650	-0.0026630

Fig.1. TS **C→D** (R^1=R^2=R^3=H)
(PCM for CH_2Cl_2)

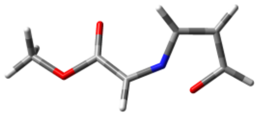

E =-513.17245266, **H (0K)** = -513.045432,
H (298K) = -513.035528, **G (298K)** = -513.080006 au
Imaginary frequency = 1.

C	-1.5099780	0.9222570	-0.4657410
N	-0.9783730	-0.2867940	-0.3281080
O	-2.0058590	-1.1280820	0.2912540
C	-3.0972870	-0.3461340	0.3994200
C	-2.8365810	0.9358340	0.0177760
C	0.2809180	-0.7882330	-0.4145780
C	1.3760420	0.0306100	-0.0186560
O	1.3364510	1.2088550	0.3494680
O	2.5596690	-0.6599800	-0.1235530
C	3.7343080	0.0883850	0.2022480
H	-4.0095200	-0.8551070	0.6777520
H	-3.5236450	1.7671800	-0.0113590
H	0.3846520	-1.8164830	-0.7260740
H	4.5714440	-0.5931310	0.0415830
H	3.8363480	0.9662520	-0.4431900
H	3.7171450	0.4189540	1.2452650
H	-0.9344260	1.6972370	-0.9473880

Fig.1. Molecule **D** (R^1=R^2=R^3=H)
(PCM for CH_2Cl_2)

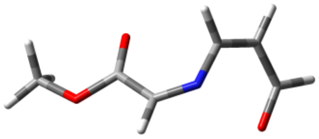

E = -513.23042708, **H (0K)** = -513.103354,
H (298K) = -513.091950, **G (298K)** = -513.140778 au.
Imaginary frequency = 0.

C	1.3649030	0.9321040	0.6742780
N	0.7892510	-0.2001760	1.2069580
O	2.5725890	-1.3880420	-0.6160670
C	2.9985800	-0.2463420	-0.7673170
C	2.4111590	0.9525670	-0.1899370
C	-0.4078510	-0.5923820	1.0581870
C	-1.4005830	0.0742620	0.1421420

Fig.1. TS **D→E** (R^1=R^2=R^3=H)
(PCM for CH_2Cl_2)

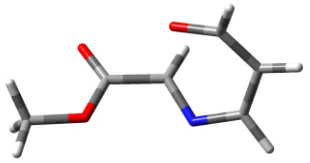

E = -513.21537192, **H (0K)** = -513.087642,
H (298K) = -513.077426, **G (298K)** = -513.123238 au
Imaginary frequency = 1.

C	-2.0885940	-1.2507930	0.1780530
N	-0.7590610	-1.2476450	0.3041200
O	-0.9388620	1.3447390	-0.3761540
C	-2.1487700	1.0438750	-0.5973560
C	-2.8285160	-0.1097340	-0.1390030
C	-0.2046350	-0.1152190	0.7061060
C	1.2639520	0.1439940	0.4736150

[1] (a) Becke, A. D. *J. Chem. Phys.* **1993**, *98*, 5648. (b) Becke, A. D. *Phys. Rev. A* **1998**, *38*, 3098. (c) Lee, C.; Yang, W.; Parr, R. G. *Phys. Rev. B* **1998**, *37*, 785.

[2] Gaussian 09, Revision D.01, M. J. Frisch, G. W. Trucks, H. B. Schlegel, G. E. Scuseria, M. A. Robb, J. R. Cheeseman, G. Scalmani, V. Barone, B. Mennucci, G. A. Petersson, H. Nakatsuji, M. Caricato, X. Li, H. P. Hratchian, A. F. Izmaylov, J. Bloino, G. Zheng, J. L. Sonnenberg, M. Hada, M. Ehara, K. Toyota, R. Fukuda, J. Hasegawa, M. Ishida, T. Nakajima, Y. Honda, O. Kitao, H. Nakai, T. Vreven, J. A. Montgomery, Jr., J. E. Peralta, F. Ogliaro, M. Bearpark, J. J. Heyd, E. Brothers, K. N. Kudin, V. N. Staroverov, T. Keith, R. Kobayashi, J. Normand, K. Raghavachari, A. Rendell, J. C. Burant, S. S. Iyengar, J. Tomasi, M. Cossi, N. Rega, J. M. Millam, M. Klene, J. E. Knox, J. B. Cross, V. Bakken, C. Adamo, J. Jaramillo, R. Gomperts, R. E. Stratmann, O. Yazyev, A. J. Austin, R. Cammi, C. Pomelli, J. W. Ochterski, R. L. Martin, K. Morokuma, V. G. Zakrzewski, G. A. Voth, P. Salvador, J. J. Dannenberg, S. Dapprich, A. D. Daniels, O. Farkas, J. B. Foresman, J. V. Ortiz, J. Cioslowski, and D. J. Fox, Gaussian, Inc., Wallingford CT, **2013**.

O	-1.1819630	1.1005600	-0.4707230
O	-2.5454900	-0.6154730	0.1172550
C	-3.5909630	-0.0775480	-0.7224440
H	3.9159670	-0.0712250	-1.3672970
H	2.8483970	1.9115810	-0.4480530
H	-0.7553850	-1.4626060	1.6136750
H	-4.4298750	-0.7633130	-0.6157980
H	-3.8643580	0.9246420	-0.3848970
H	-3.2537720	-0.0365210	-1.7605360
H	1.0017030	1.8663570	1.1010310

O	1.8919520	0.9330050	1.1511010
O	1.7567790	-0.5583590	-0.5483000
C	3.1547210	-0.3436930	-0.8361940
H	-2.6865350	1.7080710	-1.2872880
H	-3.8799020	-0.2057440	-0.3839400
H	-0.5861600	0.3941230	1.5921480
H	3.3767620	-0.9868920	-1.6865040
H	3.3312180	0.7045610	-1.0879970
H	3.7652750	-0.6201960	0.0264820
H	-2.5751370	-2.2260780	0.1737670

Fig.1. Molecule **E** ($R^1=R^2=R^3=H$)
(PCM for CH_2Cl_2)

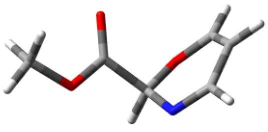

E = -513.23663388, **H (0K)** = -513.106356,
H (298K) = -513.096207, **G (298K)** = -513.141810 au.
Imaginary frequency = 0.

C	1.6450620	1.3663950	-0.4231960
N	0.7061000	1.3914070	0.4602710
O	1.2121430	-0.9834070	0.8770250
C	2.0984560	-0.9477050	-0.1385930
C	2.3192660	0.1623460	-0.8703110
C	0.2441540	0.0909150	0.9140650
C	-0.9897870	-0.3438470	0.0900810
O	-1.0257390	-1.3039860	-0.6476010
O	-2.0072960	0.4958840	0.3128940
C	-3.2237300	0.2220960	-0.4157760
H	2.6380590	-1.8839550	-0.2407080
H	3.0615710	0.1820290	-1.6574270
H	-0.0494140	0.1609570	1.9643310
H	-3.9260530	0.9955670	-0.1079950
H	-3.0399500	0.2750470	-1.4912490
H	-3.6042900	-0.7688080	-0.1576980
H	1.9839840	2.3301860	-0.8073150

Fig.1. Molecule **D** ($R^1=Me$, $R^2=R^3=H$)
(PCM for CH_2Cl_2)

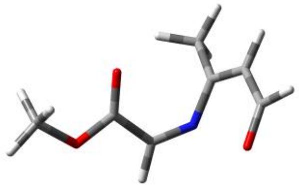

E = -552.55247688, **H (0K)** = -552.397327,
H (298K) = -552.384349, **G (298K)** = -552.436481 au.
Imaginary frequency = 0.

C	-1.5189242	0.5987187	0.3260934
N	-0.8068852	0.0906907	1.3985724
O	-1.8870142	-2.3294503	0.4661794
C	-2.4957892	-1.6024263	-0.3166816
C	-2.3445352	-0.1652303	-0.4433496
C	0.4251508	-0.1926753	1.4672904
C	1.3884418	-0.1147473	0.3086634
O	1.1195088	0.3373547	-0.7860986
O	2.5775128	-0.6078973	0.6719974
C	3.6059578	-0.5952673	-0.3422276
H	-3.2540232	-2.0410353	-1.0001746
H	-2.9512542	0.3353667	-1.1913196
H	0.8365938	-0.5352353	2.4164774
H	4.4818888	-1.0395543	0.1277134
H	3.2887708	-1.1842753	-1.2055226
H	3.8121898	0.4313287	-0.6534686
C	-1.4622962	2.1012107	0.2046004
H	-0.4391312	2.4175177	-0.0238386
H	-2.1295572	2.4498597	-0.5862806
H	-1.7542452	2.5716557	1.1501434

Fig.1. TS **D→E** ($R^1=Me$, $R^2=R^3=H$)
(PCM for CH_2Cl_2)

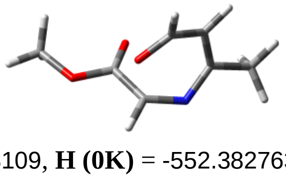

E = -552.53788109, **H (0K)** = -552.382763,
H (298K) = -552.370811, **G (298K)** = -552.420287 au.
Imaginary frequency = 1.

C	2.2551435	-0.3565176	-0.1864388
N	1.4676605	-0.4987666	0.8876302
O	0.2889625	1.7928874	0.1000692
C	1.3087205	1.8497604	-0.6581458
C	2.1766345	0.7936474	-0.9873228
C	0.2434695	-0.0308966	0.9146532
C	-0.7797045	-0.4667956	-0.1228528
O	-0.5315875	-1.1325156	-1.1051308
O	-1.9957475	-0.0317656	0.2282482
C	-3.0673275	-0.3557466	-0.6819578
H	1.5559785	2.8489194	-1.0441128

Fig.1. Molecule **E** ($R^1=Me$, $R^2=R^3=H$)
(PCM for CH_2Cl_2)

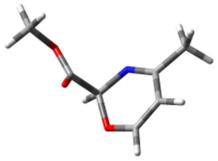

E = -552.56023310, **H (0K)** = 552.402351,
H (298K) = --552.390549, **G (298K)** = -552.439953 au.
Imaginary frequency = 0.

C	1.6948770	0.7325900	0.0317150
N	0.7059030	0.7987240	-0.7980580
O	0.5568240	-1.6606030	-0.6946040
C	1.5191540	-1.6444460	0.2505040
C	2.0786240	-0.5069970	0.7016080
C	-0.1015000	-0.3965420	-0.9413390
C	-1.3315070	-0.3075550	-0.0089530
O	-1.5257710	-1.0164760	0.9541750
O	-2.1462170	0.6727950	-0.4186670
C	-3.3244610	0.8918270	0.3863720

H	2.9712355	0.9994984	-1.6949318
H	-0.1825325	0.2386424	1.8795192
H	-3.9679395	0.0562784	-0.2288078
H	-2.8837275	0.1007514	-1.6574038
H	-3.1527115	-1.4389376	-0.7951148
C	3.3499585	-1.3686586	-0.3730568
H	2.9288545	-2.2687016	-0.8394608
H	4.1510835	-0.9940576	-1.0146508
H	3.7620915	-1.6666316	0.5956082

H	1.8123540	-2.6459800	0.5494970
H	2.8734300	-0.5302680	1.4364960
H	-0.4565530	-0.4674200	-1.9727040
H	-3.8549720	1.7133660	-0.0932220
H	-3.9436380	-0.0080900	0.4040900
H	-3.0410170	1.1603920	1.4066280
C	2.5255630	1.9651430	0.2666830
H	2.5201240	2.2247500	1.3327860
H	3.5710450	1.7870560	-0.0130840
H	2.1347110	2.8052790	-0.3108650

Fig.1. Molecule **C** (R^1=R^3=H, R^2=Me)
(PCM for CH_2Cl_2)

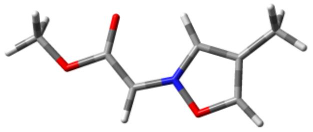

E = -552.49567264, **H (0K)** = --552.339706,
H (298K) = -552.327537, **G (298K)** = -552.377399 au.
Imaginary frequency = 0.

C	-1.1822690	0.6171370	0.0006260
N	-0.5274100	-0.5484510	0.0003390
O	-1.4388140	-1.5833630	-0.0004330
C	-2.6854570	-0.9985740	-0.0005810
C	-2.5826230	0.3545390	0.0001930
C	0.7922000	-0.9296990	0.0004090
C	1.7958250	0.0627470	-0.0000450
O	1.6273350	1.2920080	-0.0003650
O	3.0510050	-0.4994310	-0.0001140
C	4.1368870	0.4308340	-0.0004230
H	-3.5042510	-1.7002870	-0.0009400
H	0.9741400	-1.9915900	0.0008200
H	5.0435200	-0.1766450	-0.0001930
H	4.1145620	1.0676740	-0.8903000
H	4.1145220	1.0683120	0.8889950
H	-0.6127660	1.5318360	0.0012360
C	-3.6827370	1.3710480	0.0004510
H	-3.6215250	2.0160950	0.8840210
H	-3.6213730	2.0166690	-0.8826880
H	-4.6621250	0.8851800	0.0002030

Fig.1. TS **C→D** (R^1=R^3=H, R^2=Me)
(PCM for CH_2Cl_2)

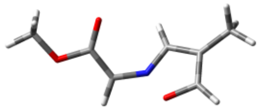

E = -552.49261312, **H (0K)** = -552.337870,
H (298K) = -552.326120, **G (298K)** = -552.375024 au.
Imaginary frequency = 1.

C	-1.2254720	0.5170400	0.4869320
N	-0.5154250	-0.5938310	0.3437710
O	-1.3988820	-1.5681920	-0.3110780
C	-2.5897550	-0.9488270	-0.4225220
C	-2.5402720	0.3551130	-0.0180020
C	0.8045500	-0.8957910	0.4404960
C	1.7648770	0.0683560	0.0213330
O	1.5515700	1.2193780	-0.3718610
O	3.0387980	-0.4361570	0.1354750
C	4.0877450	0.4713880	-0.2128260
H	-3.4179110	-1.5782650	-0.7197070
H	1.0636310	-1.8929540	0.7632230
H	5.0180080	-0.0688140	-0.0285090
H	4.0277440	0.7615530	-1.2664340
H	4.0501680	1.3753130	0.4029380
H	-0.7823540	1.3605500	0.9945250
C	-3.6472670	1.3644330	0.0045930
H	-3.7913430	1.7742120	1.0101100
H	-3.4291900	2.2031150	-0.6658890
H	-4.5891090	0.9116090	-0.3169550

Fig.1. Molecule **D** (R^1=R^3=H, R^2=Me)
(PCM for CH_2Cl_2)

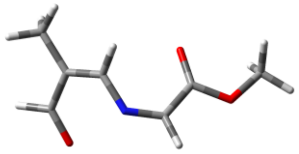

E = -552.54763602, **H (0K)** = -552.392844,
H (298K) = -552.379772, **G (298K)** = -552.432354 au.
Imaginary frequency = 0.

C	-1.0918020	0.3615190	0.7874030
N	-0.3727400	-0.7931040	1.0310190
O	-2.1398280	-1.8050310	-0.7996030
C	-2.7330360	-0.7405780	-0.6643500
C	-2.2293090	0.4364420	0.0476680
C	0.8706810	-0.9551340	0.8220990
C	1.7523800	0.0419990	0.1214460
O	1.3841690	1.1308960	-0.2749630
O	2.9961840	-0.4403150	0.0070820
C	3.9521240	0.4224750	-0.6460670

Fig.1. TS **D→E** (R^1=R^3=H, R^2=Me)
(PCM for CH_2Cl_2)

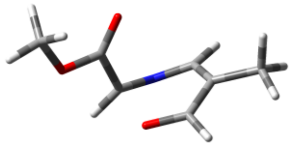

E = -552.53022380, **H (0K)** = -552.375025,
H (298K) = --552.363073, **G (298K)** = -552.412513 au.
Imaginary frequency = 1.

C	1.6187890	1.1081660	0.2317830
N	0.6590450	1.6547430	-0.5189460
O	0.2274670	-0.8691610	-1.3437920
C	1.3429100	-1.0684090	-0.7705770
C	1.9807680	-0.2429870	0.1857960
C	-0.4113320	0.9709550	-0.8451900
C	-1.3107190	0.3587110	0.2178400
O	-1.0694620	0.3313410	1.4051170
O	-2.4239420	-0.1140790	-0.3555580
C	-3.3699430	-0.7458690	0.5319770
H	1.9021790	-1.9502800	-1.1183670

H	-3.7553220	-0.6201670	-1.0835390
H	1.3467270	-1.8764630	1.1561650
H	4.8889610	-0.1325110	-0.6495840
H	3.6270970	0.6392040	-1.6661880
H	4.0578520	1.3561480	-0.0889240
H	-0.7521030	1.2401250	1.3349440
C	-3.0237070	1.7165300	-0.0456320
H	-2.6107820	2.4892520	0.6094080
H	-3.0311340	2.1162160	-1.0677810
H	-4.0703010	1.5560080	0.2428370

H	-0.9234680	1.2279760	-1.7709070
H	-4.2019370	-1.0518300	-0.1007540
H	-2.9130210	-1.6135440	1.0135740
H	-3.7042060	-0.0378760	1.2938490
H	2.2749430	1.8097600	0.7485840
C	3.2214180	-0.7364600	0.8934100
H	4.0089290	0.0251120	0.9221050
H	3.0047610	-1.0204730	1.9315050
H	3.6346560	-1.6215030	0.3966660

Fig.1. Molecule **E** (R^1=R^3=H, R^2=Me)
(PCM for CH_2Cl_2)

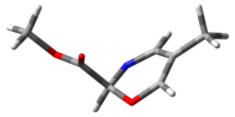

E = -552.55492279, **H (0K)** = -552.396789,
H (298K) = -552.396789, **G (298K)** = -552.434394 au.
Imaginary frequency = 0.

C	-1.3550140	-1.2697210	-0.3309710
N	-0.2686900	-1.1634930	-1.0148220
O	-0.6400650	1.2615240	-0.9765550
C	-1.6953940	1.0621720	-0.1508970
C	-2.0873670	-0.1588740	0.2658870
C	0.2970720	0.1732300	-1.0783570
C	1.3709120	0.3419430	0.0223190
O	1.3000790	1.1242200	0.9444500
O	2.3883910	-0.4995920	-0.1972660
C	3.4590610	-0.4621010	0.7708910
H	-2.2144420	1.9893930	0.0740010
H	0.7796820	0.3103830	-2.0495140
H	4.1837490	-1.2005580	0.4305650
H	3.9082050	0.5333000	0.8000270
H	3.0814050	-0.7220210	1.7624000
H	-1.7823150	-2.2718030	-0.2433840
C	-3.2728250	-0.3904260	1.1620900
H	-4.0067810	-1.0557280	0.6899630
H	-2.9771590	-0.8568460	2.1104050
H	-3.7774170	0.5517710	1.3984910

Fig.1. Molecule **C** (R^1=R^2=H, R^3=Me)
(PCM for CH_2Cl_2)

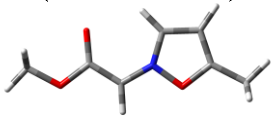

E = -552.50061174, **H (0K)** = -552.344517,
H (298K) = -552.332431, **G (298K)** = -552.381991 au.
Imaginary frequency = 0.

C	0.9151050	1.2101700	-0.0000100
N	0.5416180	-0.0728810	0.0000010
O	1.6674480	-0.8734560	0.0000140
C	2.7645730	-0.0318060	0.0000110
C	2.3319010	1.2561300	0.0000000
C	-0.6579880	-0.7475160	0.0000220
C	-1.8588420	-0.0091000	0.0000310
O	-1.9732080	1.2272200	0.0000220
O	-2.9568550	-0.8394130	-0.0000230
C	-4.2222950	-0.1746560	-0.0000490
H	2.9423480	2.1462870	-0.0000050
H	-0.5920050	-1.8227870	-0.0000390
H	-4.9711900	-0.9686150	-0.0000790
H	-4.3426870	0.4518230	0.8893440
H	-4.3426400	0.4518440	-0.8894330
H	0.1541820	1.9723580	-0.0000260
C	4.0875750	-0.7047310	0.0000110
H	4.2125340	-1.3386440	0.8857160
H	4.2125380	-1.3386320	-0.8857020
H	4.8763530	0.0507740	0.0000180

Fig.1. TS **C→D** (R^1=R^2=H, R^3=Me)
(PCM for CH_2Cl_2)

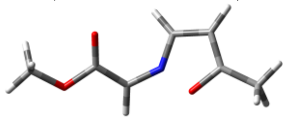

E = -552.49828375, **H (0K)** = -552.343246,
H (298K) = -552.331698, **G (298K)** = -552.379914 au.
Imaginary frequency = 1.

C	0.9599060	1.0048672	-0.4841840
N	0.5284040	-0.2497938	-0.4513510
O	1.6386920	-1.0624098	0.0184610
C	2.6921570	-0.2218618	0.1675350
C	2.3046890	1.0688632	-0.0652710
C	-0.6971860	-0.8407868	-0.5345720
C	-1.8254620	-0.1594978	-0.0061430
O	-1.8581710	0.9732372	0.4885050
O	-2.9617370	-0.9268588	-0.1350210
C	-4.1670320	-0.3119758	0.3260010
H	2.9261280	1.9504152	-0.0310790
H	-0.7397420	-1.8322808	-0.9590370

Fig.1. Molecule **D** (R^1=R^2=H, R^3=Me)
(PCM for CH_2Cl_2)

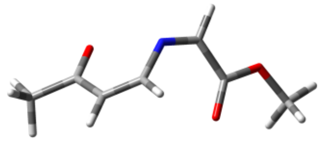

E = -552.55508841, **H (0K)** = -552.400151,
H (298K) = -552.387118, **G (298K)** = -552.440146 au.
Imaginary frequency = 0.

C	-0.8238969	0.8418341	1.0518935
N	-0.1950930	1.2868474	-0.0949075
O	-2.1627504	-0.3173379	-1.2177439
C	-2.6419835	-0.4149612	-0.0880415
C	-1.9671060	0.1185194	1.1011534
C	0.9735373	0.9820752	-0.4842322
C	1.8492226	-0.0403064	0.1900100
O	1.5697304	-0.6062263	1.2285937
O	2.9749874	-0.2211312	-0.5106517
C	3.9110573	-1.1778412	0.0317179
H	-2.4110567	-0.0604761	2.0752721
H	1.3852309	1.4762612	-1.3636840

H	-4.9629640	-1.0285888	0.1151360
H	-4.1242710	-0.1090388	1.4008190
H	-4.3600500	0.6280272	-0.2002960
H	0.2983420	1.7789782	-0.8403370
C	4.0100480	-0.8505738	0.4519090
H	3.9733450	-1.4520918	1.3666990
H	4.3079740	-1.5108898	-0.3708540
H	4.7694130	-0.0746488	0.5711990

H	4.7448534	-1.1969718	-0.6683023
H	4.2441982	-0.8574324	1.0214970
H	3.4428090	-2.1621193	0.1030144
H	-0.3955321	1.2288368	1.9758308
C	-3.9879577	-1.0813940	0.1425045
H	-4.6927897	-0.3779047	0.6026324
H	-4.3954161	-1.4371813	-0.8059020
H	-3.8812957	-1.9254311	0.8353170

Fig.1. TS **D→E** (R^1=R^2=H, R^3=Me)
(PCM for CH_2Cl_2)

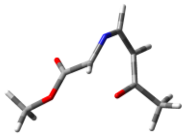

E = -552.53895755, **H (0K)** = -552.383646,
H (298K) = -552.371749, **G (298K)** = -552.421101 au.
Imaginary frequency = 1.

C	-1.3781920	1.7039060	0.2794250
N	-0.5240550	1.7852040	-0.7442220
O	-0.6912510	-0.9119020	-0.7280290
C	-1.6858600	-0.7136280	0.0418410
C	-1.9414890	0.5088310	0.7189130
C	0.3207490	0.8174550	-0.9987070
C	1.3324700	0.3612330	0.0405670
O	1.3682880	0.7381750	1.1917450
O	2.1980440	-0.4930840	-0.5196880
C	3.2110670	-1.0275500	0.3573450
H	-2.7898660	0.5539690	1.3915490
H	0.6371900	0.6653020	-2.0294750
H	3.8221630	-1.6779700	-0.2669800
H	3.8130010	-0.2183490	0.7772010
H	2.7469760	-1.5960480	1.1667390
H	-1.7747660	2.6488170	0.6502610
C	-2.6866460	-1.8428940	0.1449750
H	-3.3197040	-1.8496530	-0.7511440
H	-2.1685100	-2.8048460	0.1928310
H	-3.3313340	-1.7272760	1.0201900

Fig.1. Molecule **E** (R^1=R^2=H, R^3=Me)
(PCM for CH_2Cl_2)

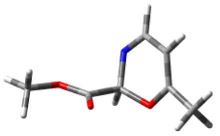

E = -552.56300227, **H (0K)** = -552.404878,
H (298K) = -552.393180, **G (298K)** = -552.442155 au.
Imaginary frequency = 0.

C	-0.9507653	1.4633982	-0.2414197
N	-0.0160433	1.3529682	0.6418663
O	-1.0407033	-0.7981978	1.2395213
C	-1.9078393	-0.6802838	0.2012523
C	-1.8666023	0.4046522	-0.6072807
C	0.1482447	0.0160362	1.1841203
C	1.2243237	-0.7518928	0.3816953
O	1.0189227	-1.7552638	-0.2662257
O	2.4124637	-0.1491278	0.5085253
C	3.5069507	-0.7530068	-0.2141247
H	-2.5785183	0.5304712	-1.4127997
H	0.4805237	0.0874672	2.2229623
H	4.3773467	-0.1380928	0.0108863
H	3.6629207	-1.7800678	0.1237663
H	3.2995737	-0.7484088	-1.2866397
H	-1.0791893	2.4457912	-0.6999207
C	-2.8850493	-1.8053828	0.1446563
H	-3.4071163	-1.8997978	1.1043383
H	-3.6196013	-1.6470848	-0.6476837
H	-2.3581413	-2.7502738	-0.0333247

Fig.1. Molecule **D** (R^1=R^3=Me, R^2=H)
(PCM for CH_2Cl_2)

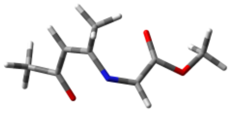

E = -591.87664355, **H (0K)** = -591.693591,
H (298K) = -591.678970, **G (298K)** = -591.735303 au.
Imaginary frequency = 0.

C	-0.9285680	1.2208580	0.0961400
N	-0.1979390	0.8203970	1.2067800
O	-1.7540530	-1.4575110	0.9007620
C	-2.3529330	-0.8347980	0.0218710
C	-1.9573850	0.5018010	-0.4248680
C	-0.6130400	2.6237430	-0.3634430
H	-1.2955600	2.9327300	-1.1579580
H	-0.6972560	3.3283420	0.4715270
H	0.4151280	2.6707730	-0.7375590
C	-3.5803940	-1.4267830	-0.6550130
H	-3.4304040	-1.4856370	-1.7401500
H	-3.7777690	-2.4253570	-0.2597170
H	-4.4573340	-0.7890380	-0.4878670
C	0.9674200	0.3257110	1.2408170

Fig.1. TS **D→E** (R^1=R^3=Me, R^2=H)
(PCM for CH_2Cl_2)

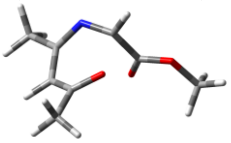

E = -591.86235335, **H (0K)** = -591.679486,
H (298K) = -591.665833, **G (298K)** = -591.719310 au.
Imaginary frequency = 1.

C	2.3052433	-1.0327410	-0.0975161
N	1.4565793	-1.1955120	0.9302569
O	0.8266263	1.3911820	0.5501139
C	1.8758773	1.3918240	-0.1745971
C	2.5108663	0.2263090	-0.6719581
C	3.1682603	-2.2086770	-0.4609281
H	4.0036613	-1.9207640	-1.1032841
H	3.5519373	-2.6914930	0.4436209
H	2.5613743	-2.9526560	-0.9921841
C	2.5001963	2.7451620	-0.4324141
H	3.2349703	2.7027410	-1.2405661
H	1.7261013	3.4783800	-0.6776951
H	3.0054753	3.0900650	0.4782879
C	0.3544873	-0.4962200	1.0218999

C	1.7862510	-0.0371310	0.0274070
O	1.4769590	0.2124390	-1.1204120
O	2.9058930	-0.6661500	0.4055280
C	3.7910300	-1.0774220	-0.6587400
H	4.6337510	-1.5592730	-0.1655260
H	3.2794150	-1.7778800	-1.3226180
H	4.1223310	-0.2068050	-1.2293070
H	1.4251610	0.1202770	2.2084370
H	-2.5365650	0.9629850	-1.2187900

C	-0.6797437	-0.5075810	-0.0925041
O	-0.5299007	-1.0305810	-1.1760951
O	-1.7934037	0.1156610	0.3142379
C	-2.8585687	0.1961130	-0.6548241
H	-3.6755667	0.7074500	-0.1474471
H	-2.5317417	0.7658220	-1.5279841
H	-3.1640977	-0.8056010	-0.9658681
H	-0.0594497	-0.3141800	2.0125209
H	3.3631673	0.3597000	-1.3278891

Fig.1. Molecule **E** (R^1=R^3=Me, R^2=H)
(PCM for CH_2Cl_2)

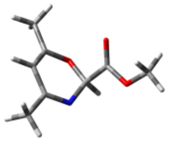

E = -591.88635254, **H (0K)** = -591.700742,
H (298K) = -591.687305, **G (298K)** = -591.740323 au.
Imaginary frequency = 0.

C	-1.1647720	1.4043840	-0.0474760
N	-0.1729260	1.2829480	0.7745040
O	-0.7316870	-1.1048880	0.9403640
C	-1.6658660	-0.9493990	-0.0331930
C	-1.8788410	0.2600290	-0.5970270
C	-1.6379760	2.7877780	-0.4065420
H	-2.6837820	2.9297670	-0.1077910
H	-1.0175970	3.5420960	0.0817890
H	-1.5939160	2.9318620	-1.4933830
C	-2.4106540	-2.2113190	-0.3136110
H	-3.2060890	-2.0432790	-1.0427740
H	-1.7233120	-2.9744470	-0.6973230
H	-2.8497990	-2.6041560	0.6112340
C	0.2709060	-0.0727640	1.0292740
C	1.4255840	-0.4394970	0.0681710
O	1.3723400	-1.2992260	-0.7846710
O	2.4925110	0.3315010	0.3134780
C	3.6380290	0.1133380	-0.5373050
H	4.3955330	0.8146010	-0.1896050
H	3.9923490	-0.9155070	-0.4401190
H	3.3788490	0.3133510	-1.5796010
H	0.6450360	-0.1410110	2.0542950
H	-2.6505630	0.3916990	-1.3453650

Fig.1. Molecule **D** (R^1=R^3=Me, R^2=Ph)
(PCM for CH_2Cl_2)

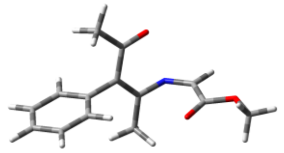

E = -822.92593224, **H (0K)** = -822.662053,
H (298K) = -822.642799, **G (298K)** = -822.710614 au.
Imaginary frequency = 0.

C	0.2172149	-0.0011628	1.1928884
N	-0.9502401	0.6854622	1.4989554
O	-0.1180101	2.3614122	-0.3279876
C	0.9296549	1.7212762	-0.4296706
C	1.1665059	0.4717122	0.3284354
C	0.4029869	-1.2222788	2.0642204
H	1.3972839	-1.6511008	1.9434614
H	0.2531289	-0.9569588	3.1165264
H	-0.3464581	-1.9764748	1.8013064
C	2.0342909	2.2212032	-1.3479826
H	2.9591119	2.4075582	-0.7907566
H	2.2729729	1.4735422	-2.1130336
H	1.7047079	3.1445802	-1.8284056
C	2.4519189	-0.2739248	0.1303514
C	3.6084629	0.0687632	0.8493094
C	2.5285599	-1.3243228	-0.7982556
C	4.8073139	-0.6174578	0.6452984
H	3.5637599	0.8764922	1.5756884
C	3.7262489	-2.0124168	-1.0035306
H	1.6381509	-1.6022878	-1.3563006
C	4.8696019	-1.6595778	-0.2829666
H	5.6913669	-0.3393998	1.2130644
H	3.7651549	-2.8244218	-1.7248786
H	5.8022039	-2.1942038	-0.4419156
C	-2.1317761	0.4206992	1.1236764
C	-2.4965801	-0.6270248	0.1053834
O	-1.7145391	-1.3991518	-0.4141266
O	-3.8127901	-0.5845268	-0.1409436
C	-4.3068161	-1.5369408	-1.1067296
H	-5.3776831	-1.3518408	-1.1756306
H	-3.8239861	-1.3770688	-2.0734476
H	-4.1116581	-2.5556478	-0.7638416
H	-2.9575321	1.0046422	1.5296244

Fig.1. TS **D→E** (R^1=R^3=Me, R^2=Ph)
(PCM for CH_2Cl_2)

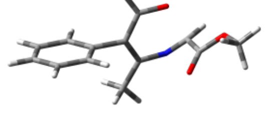

E = -822.91101542, **H (0K)** = -822.647647,

Fig.1. Molecule **E** (R^1=R^3=Me, R^2=Ph)
(PCM for CH_2Cl_2)

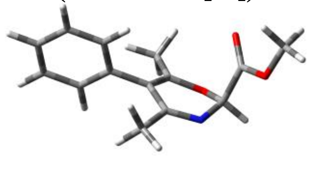

E = -822.93794010, **H (0K)** = -822.671342,

H (298K) = -822.629151, **G (298K)** = -822.695099 au.
Imaginary frequency = 1.

C	0.2889548	1.5474251	-0.1362978
N	-0.8979202	1.8589061	-0.6839928
O	-1.0639622	-0.7277089	-1.3301478
C	0.1765418	-0.7394329	-1.0449158
C	0.8526318	0.2661771	-0.2800788
C	-1.8815042	1.0038111	-0.7299078
C	-2.3905672	0.3099361	0.5240722
O	-1.8773522	0.3822031	1.6202122
O	-3.5123002	-0.3655569	0.2422352
C	-4.1025222	-1.0923079	1.3394482
H	-2.6308252	1.1181481	-1.5119148
H	-5.0081532	-1.5433509	0.9360022
H	-3.4145662	-1.8634899	1.6942722
H	-4.3423272	-0.4113449	2.1593032
C	1.0700078	2.6964831	0.4483662
H	1.4794818	2.4194931	1.4248372
H	1.9191928	2.9703811	-0.1879618
H	0.4210528	3.5677171	0.5598842
C	0.9595888	-1.9084219	-1.6101188
H	1.8198588	-1.5728299	-2.1973778
H	1.3511728	-2.5227159	-0.7912878
H	0.3012718	-2.5190999	-2.2306868
C	2.2744228	0.0279251	0.1330322
C	3.3543288	0.4535731	-0.6581528
C	2.5540208	-0.6359999	1.3382982
C	4.6719828	0.2232161	-0.2581268
H	3.1567018	0.9656081	-1.5968998
C	3.8709468	-0.8684229	1.7413802
H	1.7281438	-0.9680229	1.9623222
C	4.9339388	-0.4391239	0.9436712
H	5.4932188	0.5597381	-0.8855308
H	4.0655178	-1.3832849	2.6785712
H	5.9590958	-0.6190849	1.2560192

H (298K) = -822.653197, **G (298K)** = -822.719111 au.
Imaginary frequency = 0.

C	0.4552678	-0.5601773	-0.9739027
N	1.7057958	-0.3894393	-1.2529977
O	1.5543098	1.7932147	-0.1782017
C	0.2932528	1.5069907	0.2464313
C	-0.2979812	0.3246887	-0.0669737
C	2.3744798	0.6710317	-0.5310857
C	3.0289378	0.1137947	0.7552813
O	2.7206398	0.4303687	1.8841303
O	3.9867928	-0.7675083	0.4412823
C	4.6697018	-1.3813423	1.5551383
H	3.1642748	1.0910697	-1.1597577
H	5.4033738	-2.0529973	1.1112413
H	5.1632348	-0.6196333	2.1631593
H	3.9617498	-1.9391613	2.1724233
C	-0.2670992	-1.6871583	-1.6666597
H	-0.6490852	-2.4080403	-0.9346747
H	-1.1343352	-1.3146843	-2.2238957
H	0.4146658	-2.1962653	-2.3505707
C	-0.2882912	2.6487997	1.0140493
H	-0.2259712	3.5677127	0.4189693
H	-1.3280392	2.4673707	1.2853583
H	0.3014078	2.8119727	1.9240893
C	-1.6827982	-0.0232113	0.3718613
C	-2.8070442	0.5770707	-0.2179327
C	-1.8873462	-0.9801563	1.3794163
C	-4.0978582	0.2365237	0.1921213
H	-2.6644412	1.3139867	-1.0040787
C	-3.1768512	-1.3251463	1.7875693
H	-1.0264332	-1.4488723	1.8492993
C	-4.2861792	-0.7166723	1.1952503
H	-4.9552712	0.7140177	-0.2747017
H	-3.3145882	-2.0652843	2.5713273
H	-5.2902882	-0.9833753	1.5136713

Fig.1. Molecule **D** (R^1=Me, R^2=R^3=H)
(PCM for CH_2Cl_2)

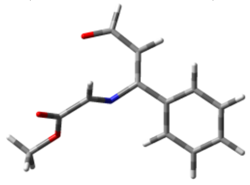

E = -744.29056615, **H (0K)** = -744.082377,
H (298K) = -744.066140, **G (298K)** = -744.127470 au.
Imaginary frequency = 0.

C	3.1102110	0.2182499	0.4485180
C	1.9232700	-0.3340821	-0.0664330
C	1.9218870	-1.6816001	-0.4709600
C	3.0831360	-2.4469591	-0.3831420
C	4.2591970	-1.8857481	0.1196790
C	4.2668820	-0.5520261	0.5385660
C	0.6860060	0.4760309	-0.1624630
N	-0.4851660	-0.2776091	-0.2073470
O	-1.6197380	2.4853059	-0.3287230
C	-0.4297490	2.7343139	-0.4995660
C	0.7076790	1.8327359	-0.3364800
C	-1.4031020	-0.1733861	0.6613040
C	-2.6828310	-0.9537281	0.5611240
O	-3.5526940	-0.8491291	1.4049680
O	-2.7453960	-1.7385941	-0.5170130
C	-3.9600830	-2.5044871	-0.6610860

Fig.1. TS **D→E** (R^1=Me, R^2=R^3=H)
(PCM for CH_2Cl_2)

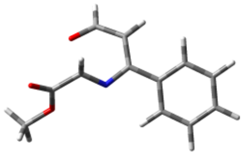

E = -744.27932804, **H (0K)** = -744.070372,
H (298K) = -744.055543, **G (298K)** = -744.113011 au.
Imaginary frequency = 1.

C	2.7114183	0.8419230	0.1660520
C	1.5483393	0.0652230	0.0222700
C	1.6774783	-1.3286470	-0.1125820
C	2.9352453	-1.9262700	-0.1194370
C	4.0851493	-1.1441410	0.0212700
C	3.9683983	0.2403260	0.1697360
C	0.2000363	0.6781790	0.0415920
N	-0.8516507	-0.1503750	0.1802000
O	-2.4057617	1.9462090	-0.3777850
C	-1.2893357	2.4963310	-0.6256070
C	-0.0097567	2.0411720	-0.2440990
C	-1.9681277	0.3639440	0.6584010
C	-3.2741727	-0.3773370	0.5034920
O	-4.2193607	-0.1759810	1.2398180
O	-3.2657317	-1.2324820	-0.5213730
C	-4.4915677	-1.9636920	-0.7331200
H	2.6386243	1.9162600	0.3008250

H	3.1206270	1.2431279	0.8056270
H	1.0102340	-2.1155811	-0.8667050
H	3.0692630	-3.4828961	-0.7097190
H	5.1627770	-2.4844891	0.1914260
H	5.1734340	-0.1133221	0.9454880
H	-0.1285570	3.7527739	-0.8226950
H	1.6764650	2.3003639	-0.4805490
H	-1.3448940	0.4677909	1.5434270
H	-3.8340890	-3.0755851	-1.5797960
H	-4.0898700	-3.1722391	0.1938070
H	-4.8210870	-1.8364591	-0.7371120

H	0.7805343	-1.9287420	-0.2191200
H	3.0194523	-3.0032960	-0.2340710
H	5.0662283	-1.6108590	0.0190660
H	4.8571073	0.8529610	0.2911530
H	-1.3332277	3.3850430	-1.2687770
H	0.8239393	2.6687540	-0.5319630
H	-1.9466067	0.9886240	1.5535970
H	-4.3002907	-2.6068430	-1.5910000
H	-4.7339617	-2.5600350	0.1495630
H	-5.3116127	-1.2732870	-0.9437650

Fig.1. Molecule **C** (R^1=R^3=H, R^2=Ph)
(PCM for CH_2Cl_2)

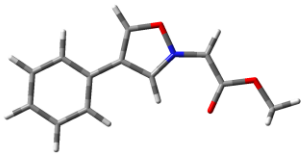

E = -744.23445112, **H (0K)** = -744.025195,
H (298K) = -744.010997, **G (298K)** = -744.065992 au.
Imaginary frequency = 0.

C	-0.0785995	0.1139331	-0.0004470
N	0.8831075	1.0406331	-0.0004500
O	0.3075345	2.2945541	-0.0001990
C	-1.0509855	2.0992601	-0.0001330
C	-1.3486155	0.7687251	-0.0001260
C	2.2550625	1.0250081	-0.0005150
C	2.9276515	-0.2173329	0.0001130
O	2.4067445	-1.3429209	0.0006510
O	4.2905285	-0.0468229	0.0000560
C	5.0556275	-1.2557459	0.0004930
H	-1.6218185	3.0132891	-0.0002330
H	2.7366415	1.9887311	-0.0011170
H	6.1005445	-0.9413049	0.0001940
H	4.8458655	-1.8570829	0.8904150
H	4.8455915	-1.8578969	-0.8888130
H	0.2135115	-0.9226029	-0.0006490
C	-2.6707985	0.1232971	-0.0000120
C	-3.8487895	0.8908741	0.0008170
C	-2.7826585	-1.2766469	-0.0007590
C	-5.0981595	0.2757441	0.0008790
H	-3.7918855	1.9757411	0.0014080
C	-4.0348975	-1.8907319	-0.0006620
H	-1.8899175	-1.8944389	-0.0014280
C	-5.1976295	-1.1185559	0.0001530
H	-5.9961115	0.8871931	0.0015160
H	-4.0988915	-2.9751769	-0.0012390
H	-6.1727465	-1.5971729	0.0002140

Fig.1. TS **C→D** (R^1=R^3=H, R^2=Ph)
(PCM for CH_2Cl_2)

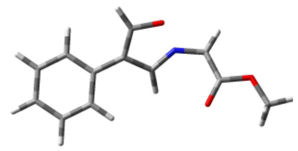

E = -744.23163805, **H (0K)** = -744.023656,
H (298K) = -744.008977, **G (298K)** = -744.065889 au.
Imaginary frequency = 1.

C	0.2262150	0.1585950	-0.4696320
N	1.2279380	1.0134220	-0.3244580
O	0.6642770	2.2079990	0.3238380
C	-0.6534220	1.9676980	0.4342560
C	-0.9882660	0.7003190	0.0321110
C	2.5767890	0.9226260	-0.4209580
C	3.2148130	-0.2905580	-0.0296690
O	2.6725640	-1.3379690	0.3343570
O	4.5789310	-0.1743470	-0.1347020
C	5.3193160	-1.3550410	0.1891390
H	-1.2531460	2.8116350	0.7459360
H	3.1142180	1.8067890	-0.7288420
H	6.3670800	-1.1006260	0.0207570
H	5.1681690	-1.6427150	1.2340470
H	5.0284470	-2.1924840	-0.4522800
H	0.4022100	-0.7692450	-0.9914150
C	-2.3123950	0.0596200	0.0095800
C	-3.4876630	0.8281570	-0.0422150
C	-2.4206520	-1.3401990	0.0461210
C	-4.7370990	0.2109640	-0.0490900
H	-3.4244500	1.9115050	-0.0947310
C	-3.6724210	-1.9550160	0.0311710
H	-1.5235940	-1.9503090	0.1050320
C	-4.8348510	-1.1826690	-0.0144100
H	-5.6356580	0.8201080	-0.0911290
H	-3.7376580	-3.0389570	0.0618790
H	-5.8095420	-1.6620890	-0.0244190

Fig.1. Molecule **D** (R^1=R^3=H, R^2=Ph)
(PCM for CH_2Cl_2)

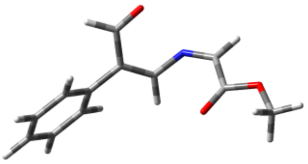

E = -744.28715505, **H (0K)** = -744.078978,
H (298K) = -744.063034, **G (298K)** = -744.123174 au.
Imaginary frequency = 0.

C	0.1620823	0.4160896	0.8022510

Fig.1. TS **D→E** (R^1=R^3=H, R^2=Ph)
(PCM for CH_2Cl_2)

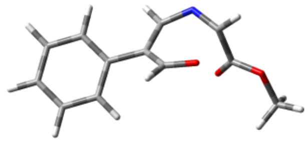

E = -744.27118766, **H (0K)** = -744.062553,
H (298K) = -744.047793, **G (298K)** = -744.104425 au.
Imaginary frequency = 1.

C	0.2036662	1.5396676	-0.4271730

N	-0.9903517	1.1172886	1.0755710
O	0.0657503	2.8087306	-0.7622080
C	1.0977063	2.1577646	-0.6457770
C	1.2104773	0.8742656	0.0579000
C	-2.1798707	0.7478696	0.8269910
C	-2.5274597	-0.4755004	0.0231490
O	-1.7117727	-1.2708754	-0.4008200
O	-3.8517477	-0.5598154	-0.1455590
C	-4.3226637	-1.6966744	-0.9020640
H	2.0510093	2.5483436	-1.0558500
H	-3.0100687	1.3468066	1.1990060
H	-5.4056327	-1.5908524	-0.9403570
H	-3.8961457	-1.6808744	-1.9074960
H	-4.0419317	-2.6245414	-0.3987010
H	0.2534753	-0.5315144	1.3302510
C	2.4826283	0.1151146	-0.0244550
C	3.1729713	0.0129176	-1.2455150
C	3.0358603	-0.5118744	1.1064330
C	4.3724853	-0.6944154	-1.3331670
H	2.7569673	0.4714926	-2.1387580
C	4.2285753	-1.2281024	1.0162070
H	2.5365623	-0.4199564	2.0673890
C	4.9037683	-1.3205304	-0.2037730
H	4.8864953	-0.7636664	-2.2880540
H	4.6381713	-1.7035974	1.9033830
H	5.8365923	-1.8732734	-0.2723080

N	1.3966522	2.0476346	-0.1341320
O	1.5331052	0.0532596	1.6626830
C	0.2958232	-0.0451064	1.4040130
C	-0.3933098	0.4826116	0.2792550
C	2.3711162	1.3083496	0.3356540
C	2.8845152	0.1109076	-0.4496060
O	2.3606282	-0.3431214	-1.4430630
O	4.0110462	-0.3421124	0.1105100
C	4.6041952	-1.5000224	-0.5154240
H	-0.3144588	-0.5302764	2.1788110
H	3.1085872	1.7763196	0.9852420
H	5.5098122	-1.7049794	0.0536080
H	3.9169982	-2.3477444	-0.4648460
H	4.8434092	-1.2833914	-1.5589370
H	-0.3937238	2.1063646	-1.1403110
C	-1.8149488	0.1146956	0.0721360
C	-2.2176128	-1.2254204	0.2203930
C	-2.7936528	1.0633156	-0.2770350
C	-3.5475738	-1.6023794	0.0335090
H	-1.4754408	-1.9818984	0.4628960
C	-4.1198668	0.6830826	-0.4816230
H	-2.5231908	2.1117336	-0.3673830
C	-4.5049538	-0.6505044	-0.3236360
H	-3.8319518	-2.6444784	0.1529830
H	-4.8571778	1.4350506	-0.7500560
H	-5.5396818	-0.9441324	-0.4768900

Fig.1. Molecule **E** (R^1=R^3=H, R^2=Ph)
(PCM for CH_2Cl_2)

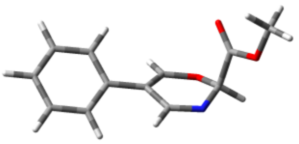

E = -744.29489979, **H (0K)** = -744.083449,
H (298K) = -744.068695, **G (298K)** = -744.126182 au.
Imaginary frequency = 0.

C	-0.1659620	-1.3916790	-0.4504300
N	-1.4157950	-1.6469660	-0.2831400
O	-1.2651330	-0.3816060	1.8140950
C	0.0047990	-0.0594120	1.5018800
C	0.6180680	-0.4652070	0.3634300
C	-2.0913640	-0.8641250	0.7337340
C	-2.7913030	0.3486480	0.0776160
O	-2.4854970	1.5053430	0.2655390
O	-3.7760710	-0.0703580	-0.7242010
C	-4.4992200	0.9624410	-1.4294780
H	0.4929280	0.4886750	2.3017360
H	-2.8509020	-1.4822640	1.2185260
H	-5.2488840	0.4386100	-2.0208500
H	-4.9746130	1.6427170	-0.7192320
H	-3.8210160	1.5232450	-2.0764820
H	0.3416510	-1.9420940	-1.2442430
C	2.0125890	-0.0993630	0.0386100
C	2.4908080	1.1953450	0.3099450
C	2.8960220	-1.0271820	-0.5423460
C	3.8118230	1.5439350	0.0301020
H	1.8134950	1.9382290	0.7224220
C	4.2128700	-0.6721910	-0.8349350
H	2.5621910	-2.0407680	-0.7460060
C	4.6785980	0.6128390	-0.5467440
H	4.1590120	2.5502250	0.2488040

Fig.1. Molecule **C** (R^1=R^2=H, R^3=Ph)
(PCM for CH_2Cl_2)

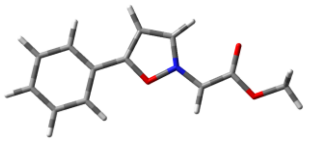

E = -744.24219765, **H (0K)** = -744.032808,
H (298K) = -744.017758, **G (298K)** = -744.075374 au.
Imaginary frequency = 0.

C	0.9260620	1.4981020	0.0008380
N	1.1059270	0.1691980	0.0000630
O	-0.1240070	-0.4543730	-0.0003440
C	-1.0898830	0.5404900	0.0001870
C	-0.4585950	1.7547930	0.0008860
C	2.1901820	-0.6697900	-0.0003480
C	3.4907180	-0.1155790	-0.0000380
O	3.7834670	1.0892700	0.0004850
O	4.4497740	-1.0985730	-0.0006100
C	5.8017620	-0.6306140	-0.0004120
H	-0.9172030	2.7314280	0.0015490
H	1.9703120	-1.7245630	-0.0008910
H	6.4226770	-1.5278920	-0.0008820
H	6.0124380	-0.0287070	-0.8897610
H	6.0124790	-0.0296090	0.8895400
H	1.7915550	2.1390700	0.0013020
C	-2.4724240	0.1014570	0.0000200
C	-2.8045670	-1.2670760	0.0009640
C	-3.5096210	1.0553310	-0.0010640
C	-4.1396680	-1.6648550	0.0008340
H	-2.0179620	-2.0142160	0.0018340
C	-4.8395100	0.6478370	-0.0011330
H	-3.2724460	2.1149830	-0.0019280
C	-5.1622740	-0.7132420	-0.0001970
H	-4.3808100	-2.7240520	0.0015750

H	4.8789480	-1.4062020	-1.2804810
H	5.7049890	0.8870590	-0.7739820

H	-5.6276980	1.3952820	-0.0019770
H	-6.2018030	-1.0278310	-0.0002820

Fig.1. TS **C→D** (R^1=R^2=H, R^3=Ph)
(PCM for CH_2Cl_2)

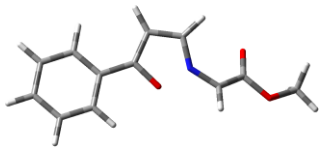

E = -744.23909604, **H (0K)** = -744.030914 ,
H (298K) = -744.016322, **G (298K)** = -744.072988 au.
Imaginary frequency = 1.

C	-0.5412104	1.6594603	-0.5333828
N	-0.7890184	0.3528563	-0.5455238
O	0.4677476	-0.3075827	-0.1839568
C	1.3994126	0.6721313	-0.0753038
C	0.8000816	1.9057293	-0.2056168
C	-1.9177144	-0.4006897	-0.5637838
C	-3.0784154	0.0786993	0.1079412
O	-3.2187764	1.1638493	0.6805632
O	-4.1003724	-0.8361817	0.0220552
C	-5.3316264	-0.4346237	0.6292072
H	1.2781526	2.8725313	-0.1849498
H	-1.8630784	-1.3668487	-1.0426528
H	-6.0295354	-1.2541457	0.4491852
H	-5.2100824	-0.2773927	1.7053862
H	-5.7125944	0.4877773	0.1800622
H	-1.3275744	2.3452233	-0.8081898
C	2.7911626	0.2637373	0.0627512
C	3.1619696	-1.0883457	-0.0573478
C	3.7819576	1.2263683	0.3329722
C	4.4966126	-1.4624107	0.0786712
H	2.4048046	-1.8378877	-0.2617428
C	5.1122286	0.8428283	0.4730182
H	3.5095936	2.2717933	0.4412612
C	5.4755066	-0.5014187	0.3444902
H	4.7722636	-2.5082367	-0.0211508
H	5.8674806	1.5944483	0.6833102
H	6.5147746	-0.7975047	0.4536722

Fig.1. Molecule **D** (R^1=R^2=H, R^3=Ph)
(PCM for CH_2Cl_2)

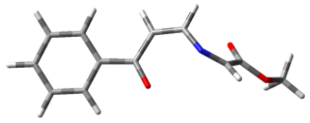

E = -744.29483267, **H (0K)** = -744.085956,
H (298K) = -744.070139, **G (298K)** = -744.130888 au.
Imaginary frequency = 0.

C	-1.0091450	1.4747180	-0.4050800
N	-1.7067900	0.7726380	-1.3669790
O	0.4692580	-0.8759740	-1.0533710
C	1.0085050	0.0333570	-0.4120030
C	0.2544970	1.2176770	0.0107660
C	-2.8159690	0.1754350	-1.2129230
C	-3.5089150	0.0118820	0.1133930
O	-3.1196210	0.5007440	1.1558960
O	-4.6124020	-0.7315150	-0.0279390
C	-5.3812420	-0.9579760	1.1735240
H	0.6989320	1.9255890	0.6989740
H	-3.3104830	-0.2577750	-2.0816730
H	-6.2221050	-1.5782260	0.8672610
H	-4.7707020	-1.4735560	1.9181670
H	-5.7304370	-0.0063210	1.5805890
H	-1.5071310	2.3794500	-0.0587780
C	2.4658860	-0.0641540	-0.0595520
C	3.0952130	-1.3111950	-0.2108480
C	3.2255980	1.0273370	0.3920580
C	4.4440160	-1.4687450	0.0949060
H	2.5035990	-2.1475250	-0.5684190
C	4.5800170	0.8718380	0.6896740
H	2.7754430	2.0092320	0.4930040
C	5.1906750	-0.3758640	0.5470940
H	4.9157160	-2.4409630	-0.0185130
H	5.1579090	1.7261970	1.0307580
H	6.2440890	-0.4964630	0.7847480

Fig.1. TS **D→E** (R^1=R^2=H, R^3=Ph)
(PCM for CH_2Cl_2)

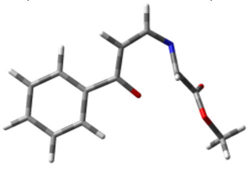

E = -744.27993650, **H (0K)** = -744.070816,
H (298K) = --744.056083, **G (298K)** = -744.113268 au.
Imaginary frequency = 1.

C	-0.7218680	2.6485310	-0.1602820
N	-1.8389750	1.9931450	0.1769180
O	-0.3530470	-0.2036620	-0.2147690
C	0.6267900	0.6096470	-0.3047490
C	0.4636690	2.0137010	-0.5171320
C	-2.0181190	0.8210110	-0.4016530
C	-2.9590140	-0.1611360	0.2560640
O	-3.3762470	-0.0708600	1.3901260
O	-3.2484480	-1.1542600	-0.5963730
C	-4.1062230	-2.1933870	-0.0820860
H	1.3470310	2.6215830	-0.6615830
H	-1.9098360	0.7200010	-1.4826040

Fig.1. Molecule **E** (R^1=R^2=H, R^3=Ph)
(PCM for CH_2Cl_2)

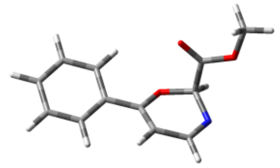

E = -744.30263354, **H (0K)** = -744.091252,
H (298K) = -744.076498, **G (298K)** = -744.134435 au.
Imaginary frequency = 0.

C	1.5407689	2.3526179	-0.1944010
N	2.3687749	1.7673079	0.6054660
O	0.5407909	0.2203139	1.1207950
C	-0.2322121	0.7820279	0.1515660
C	0.2554029	1.8183439	-0.5807340
C	1.9612219	0.4372209	1.0168710
C	2.5511419	-0.6364741	0.0714870
O	1.8949639	-1.4485581	-0.5438410
O	3.8861029	-0.5534521	0.0507930
C	4.5650549	-1.5116121	-0.7897080
H	-0.3328901	2.2924149	-1.3548220
H	2.3374849	0.2419489	2.0246750

H	-4.2312890	-2.8999730	-0.9014580
H	-3.6372780	-2.6783780	0.7772610
H	-5.0699230	-1.7742500	0.2164710
H	-0.7241490	3.7281110	-0.0087830
C	1.9821920	0.0249810	-0.1037960
C	2.1463470	-1.3607770	-0.2800790
C	3.0964510	0.8011840	0.2602020
C	3.3941930	-1.9535560	-0.1074190
H	1.2869420	-1.9589670	-0.5625420
C	4.3438310	0.2045520	0.4352750
H	2.9888690	1.8662320	0.4346480
C	4.4966350	-1.1718500	0.2495920
H	3.5091350	-3.0236150	-0.2545630
H	5.1952140	0.8136990	0.7247120
H	5.4707480	-1.6336170	0.3845070

Fig.1. Molecule **D** (R^1=R^3=Ph, R^2=H)
(PCM for CH_2Cl_2)

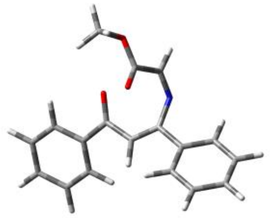

E = -975.35383800, **H (0K)** = -975.063641,
H (298K) = -975.043270, **G (298K)** = -975.114170 au.
Imaginary frequency = 0.

C	1.0315229	-0.9844170	-0.2231848
N	1.2707759	0.0121220	-1.1638028
O	-1.4814571	0.6101020	-0.3241138
C	-1.4587601	-0.5941180	-0.0299318
C	-0.2210381	-1.3439370	0.1919362
C	1.1992409	1.2639630	-0.9938138
C	0.9532359	1.9473470	0.3300982
O	1.1445469	1.4433200	1.4162992
O	0.5457279	3.2043220	0.1191192
C	0.2577869	3.9790160	1.3023922
H	-0.3034321	-2.2920470	0.7086512
H	1.3469219	1.9185320	-1.8534818
H	-0.0461191	4.9602540	0.9407482
H	-0.5494491	3.5096100	1.8692422
H	1.1494479	4.0562320	1.9286622
C	-2.7608451	-1.3263310	0.1412602
C	-3.9262651	-0.5653150	0.3309202
C	-2.8660471	-2.7256850	0.0890752
C	-5.1631821	-1.1858580	0.4828192
H	-3.8387661	0.5158450	0.3589772
C	-4.1072021	-3.3476110	0.2304192
H	-1.9856261	-3.3349680	-0.0883158
C	-5.2565201	-2.5805750	0.4333262
H	-6.0554141	-0.5856830	0.6387832
H	-4.1763001	-4.4305880	0.1786712
H	-6.2215191	-3.0664530	0.5492612
C	2.2273269	-1.7962390	0.1134962
C	3.3049209	-1.8970540	-0.7841228
C	2.3097749	-2.4610510	1.3495452
C	4.4245569	-2.6618530	-0.4610568
H	3.2508869	-1.3871750	-1.7400558
C	3.4322299	-3.2218090	1.6698142
H	1.5054919	-2.3569080	2.0710692
C	4.4920809	-3.3280340	0.7648902

H	5.6268759	-1.3006791	-0.6698800
H	4.3352969	-2.5293491	-0.4659030
H	4.2622669	-1.3818921	-1.8311960
H	1.8283119	3.3374809	-0.5668050
C	-1.5775691	0.1970959	0.0667690
C	-1.7996351	-1.1145701	0.5212130
C	-2.6529121	0.9262399	-0.4716500
C	-3.0678001	-1.6851611	0.4292120
H	-0.9687611	-1.6818581	0.9247400
C	-3.9171811	0.3504269	-0.5638660
H	-2.5036111	1.9513379	-0.7961350
C	-4.1287761	-0.9565361	-0.1137840
H	-3.2267441	-2.7018651	0.7772460
H	-4.7412661	0.9251649	-0.9766720
H	-5.1170421	-1.4023731	-0.1832960

Fig.1. TS **D→E** (R^1=R^3=Ph, R^2=H)
(PCM for CH_2Cl_2)

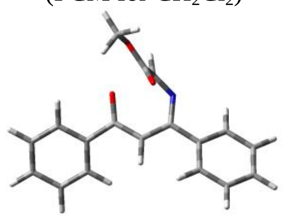

E = -975.35383800, **H (0K)** = -975.063641,
H (298K) = -975.043270, **G (298K)** = -975.114170 au.
Imaginary frequency = 1.

C	1.1581458	-0.4568390	-0.1802813
N	1.1376038	0.5657600	-1.0550223
O	-1.5098552	0.6218030	-0.6472903
C	-1.3110892	-0.5242380	-0.1087273
C	-0.0411442	-0.9982830	0.3074287
C	0.2438388	1.5144330	-0.9724743
C	0.0564418	2.3181310	0.3051817
O	0.5855708	2.0732010	1.3678827
O	-0.7540112	3.3583820	0.0694967
C	-1.0380732	4.2031030	1.2035767
H	-0.0069322	-1.9473280	0.8223857
H	-0.0180392	2.0547720	-1.8807963
H	-1.6873872	4.9909930	0.8240917
H	-1.5444132	3.6294630	1.9834837
H	-0.1125972	4.6246770	1.6027007
C	-2.5207812	-1.3854870	0.0401707
C	-3.7849472	-0.7704170	0.0578447
C	-2.4455892	-2.7844800	0.1528977
C	-4.9424212	-1.5320270	0.1988957
H	-3.8433922	0.3089640	-0.0309523
C	-3.6056052	-3.5453210	0.2901637
H	-1.4845522	-3.2857050	0.1056777
C	-4.8558912	-2.9220230	0.3169677
H	-5.9119982	-1.0424250	0.2199947
H	-3.5334342	-4.6264230	0.3676867
H	-5.7583422	-3.5170500	0.4264027
C	2.4657078	-1.1117910	0.0621457
C	3.5467798	-0.8550450	-0.7992103
C	2.6636278	-1.9700850	1.1575567
C	4.7851188	-1.4540830	-0.5816313
H	3.3981518	-0.1893910	-1.6426013
C	3.9061858	-2.5613520	1.3787257
H	1.8554218	-2.1559650	1.8575837
C	4.9693108	-2.3103280	0.5075397

H	5.2444629	-2.7393130	-1.1696248
H	3.4841009	-3.7232080	2.6321232
H	5.3670049	-3.9207120	1.0169202

H	5.6077928	-1.2530530	-1.2621713
H	4.0454238	-3.2145120	2.2354817
H	5.9362328	-2.7750880	0.6792757

Fig.1. Molecule **E** (R^1=R^3=Ph, R^2=H)
(PCM for CH_2Cl_2)

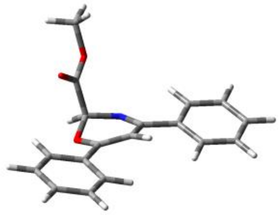

E = -975.36497092, **H (0K)** = -975.072496,
H (298K) = -975.053057, **G (298K)** = -975.122240 au.
Imaginary frequency = 0.

C	-1.1166412	-0.4711600	0.6084596
N	-1.1356972	0.6348660	1.2906886
O	1.2881768	0.4653440	1.4866516
C	1.2939888	-0.5861710	0.6236006
C	0.1239188	-1.0452430	0.1087806
C	0.1292718	1.3162320	1.4053876
C	0.3513288	2.3240410	0.2478196
O	1.3625898	2.3943220	-0.4166004
O	-0.7083842	3.1299260	0.1170216
C	-0.6042592	4.1468990	-0.9029894
H	0.1222008	-1.8565810	-0.6051704
H	0.1484438	1.8830240	2.3409246
H	-1.5384162	4.7046970	-0.8520674
H	0.2468498	4.8010380	-0.7000764
H	-0.4846922	3.6856420	-1.8859104
C	2.6346588	-1.1392890	0.3825666
C	3.7647638	-0.3147850	0.5208826
C	2.8086598	-2.4831250	0.0074346
C	5.0394068	-0.8237390	0.2790766
H	3.6283388	0.7251710	0.7947806
C	4.0843058	-2.9859540	-0.2356504
H	1.9466538	-3.1385520	-0.0701404
C	5.2032548	-2.1583740	-0.1000674
H	5.9056798	-0.1763450	0.3819616
H	4.2073058	-4.0271930	-0.5195714
H	6.1978598	-2.5540130	-0.2859454
C	-2.4116192	-1.1675340	0.3744066
C	-3.6140392	-0.4464330	0.4736486
C	-2.4684782	-2.5354960	0.0633356
C	-4.8379252	-1.0754240	0.2623166
H	-3.5679452	0.6107990	0.7116966
C	-3.6961412	-3.1664980	-0.1428654
H	-1.5558872	-3.1199060	0.0034016
C	-4.8834912	-2.4388060	-0.0468544
H	-5.7585082	-0.5027290	0.3348446
H	-3.7230962	-4.2274120	-0.3753064
H	-5.8388442	-2.9293010	-0.2122474

Fig.1. Molecule **D** (R^1=R^2=H, R^3=MeO)
(PCM for CH_2Cl_2)

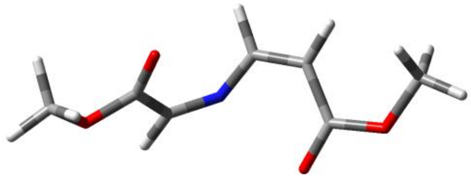

E = -627.77661668, **H (0K)** = -627.615720,
H (298K) = -627.601985, **G (298K)** = -627.656652 au.
Imaginary frequency = 0.

C	0.3545926	0.5070182	1.4563146
N	-0.4017394	1.4145692	0.7427586
O	1.4652896	0.6910482	-1.1884354
C	2.0507796	0.1032012	-0.2918764
C	1.5182226	-0.0614418	1.0644396
C	-1.5735174	1.2406172	0.2915586
C	-2.3192894	-0.0668788	0.3639166
O	-1.9227014	-1.0485128	0.9599516
O	-3.4730294	0.0206102	-0.3061254
C	-4.2925174	-1.1690698	-0.3153874
H	2.0445916	-0.6595058	1.7959146
H	-2.0879454	2.0743732	-0.1851034
H	-5.1698944	-0.9120368	-0.9066504
H	-3.7460754	-1.9968388	-0.7727284
H	-4.5774974	-1.4359038	0.7047756
O	3.2765206	-0.4132488	-0.5828754
C	4.0637546	-1.0914228	0.4059806
H	4.9966956	-1.3531448	-0.0950914
H	4.2819466	-0.4408588	1.2585636
H	3.5718006	-2.0069108	0.7497596
H	0.0154776	0.3567672	2.4805416

Fig.1. TS **D→E** (R^1=R^2=H, R^3=MeO)
(PCM for CH_2Cl_2)

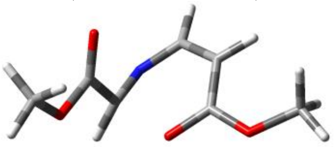

E = -627.75588357, **H (0K)** = -627.594928,

Fig.1. Molecule **E** (R^1=R^2=H, R^3=MeO)
(PCM for CH_2Cl_2)

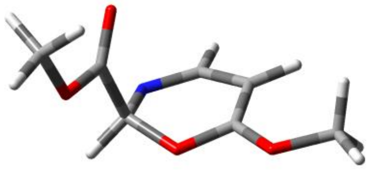

H (298K) = -627.582263, **G (298K)** = -627.633567 au.
Imaginary frequency = 1.

C	0.5918240	1.9834868	0.2985525
N	-0.3739000	2.0303998	-0.6146665
O	0.2103540	-0.5475872	-0.9885505
C	1.2156950	-0.3060942	-0.2447015
C	1.3869340	0.8643058	0.5395485
C	-1.0394300	0.9442998	-0.9417495
C	-1.9058010	0.2354488	0.0958515
O	-1.9145010	0.4861248	1.2810415
O	-2.6701010	-0.6826902	-0.5066735
C	-3.5277450	-1.4548682	0.3597195
H	2.2631700	0.9789408	1.1605355
H	-1.4069730	0.8528738	-1.9625105
H	-4.2169510	-0.7961662	0.8930285
H	-4.0722050	-2.1308722	-0.2980815
H	-2.9278460	-2.0172382	1.0789815
O	2.1482250	-1.2692542	-0.3072075
C	3.3988790	-1.1159522	0.3850845
H	3.9759160	-2.0052862	0.1323115
H	3.9262670	-0.2206392	0.0442055
H	3.2441540	-1.0741972	1.4670985
H	0.8590420	2.9278638	0.7714385

E = -627.77171599, **H (0K)** = -627.608351,
H (298K) = -627.595666, **G (298K)** = -627.647643 au.
Imaginary frequency = 0.

C	0.9262440	2.0520370	0.2851220
N	-0.1586070	2.0650320	-0.4171340
O	0.4146300	-0.2449390	-1.0630650
C	1.4527390	-0.2122090	-0.2105260
C	1.7433130	0.8917120	0.5332960
C	-0.6181670	0.7876610	-0.8604770
C	-1.6479570	0.1874120	0.1314600
O	-1.7431220	0.4779070	1.3031640
O	-2.4002770	-0.7276320	-0.4953660
C	-3.3645860	-1.4212360	0.3247750
H	2.6005840	0.9409640	1.1881980
H	-1.0833980	0.8602700	-1.8447460
H	-3.8737230	-2.1119610	-0.3460860
H	-2.8581210	-1.9648990	1.1256830
H	-4.0728530	-0.7104760	0.7566570
O	2.1227940	-1.3575390	-0.2968780
C	3.3474180	-1.4705590	0.4467780
H	3.7419030	-2.4571100	0.2071780
H	4.0555150	-0.6955870	0.1386980
H	3.1514640	-1.3964070	1.5204970
H	1.2626510	3.0086960	0.6884540

Fig.1. Molecule **D** (R^1=Ph, R^2=H, R^3=MeO)
(PCM for CH_2Cl_2)

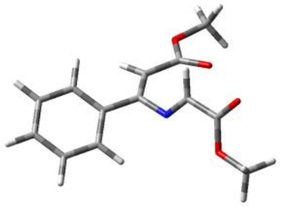

E = -858.84663390, **H (0K)** = -858.604749,
H (298K) = -858.586073, **G (298K)** = -858.653377 au.
Imaginary frequency = 0.

C	-0.0689703	0.4239429	-0.0647618
N	0.8821107	-0.5923531	-0.1874628
O	2.6521167	1.7972649	-0.0859298
C	1.5667227	2.3545479	-0.1511408
C	0.2283407	1.7515459	-0.0932838
C	1.7559367	-0.8132361	0.7044682
C	2.7971257	-1.8839301	0.5461492
O	3.6444667	-2.0718691	1.3986992
O	2.6830277	-2.5749621	-0.5909438
C	3.6639457	-3.6146751	-0.7887358
H	-0.5923123	2.4549469	-0.1641678
H	1.8285827	-0.2617231	1.6449732
H	3.4191497	-4.0637341	-1.7502588
H	4.6692147	-3.1875211	-0.8072128
H	3.5966487	-4.3554441	0.0115092
C	-1.4705433	-0.0664861	-0.0449358
C	-2.4922893	0.6875649	0.5587992
C	-1.7936853	-1.3094551	-0.6179588
C	-3.8045673	0.2199959	0.5689002
H	-2.2551393	1.6286809	1.0449502
C	-3.1086803	-1.7718101	-0.6081058
H	-1.0108963	-1.9002501	-1.0808818
C	-4.1187323	-1.0093101	-0.0174588
H	-4.5811493	0.8118489	1.0449962
H	-3.3441053	-2.7292741	-1.0642338
H	-5.1424663	-1.3726791	-0.0069798

Fig.1. TS **D→E** (R^1=Ph, R^2=H, R^3=MeO)
(PCM for CH_2Cl_2)

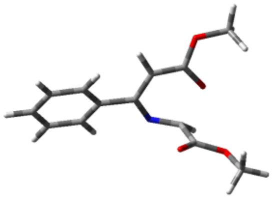

E = -858.82355810, **H (0K)** = -858.581667,
H (298K) = -858.564224, **G (298K)** = -858.627480 au.
Imaginary frequency = 1.

C	-2.5874393	0.3356461	1.0216800
C	-1.9290083	-0.2232449	-0.0864010
C	-2.6788603	-0.9673559	-1.0131070
C	-4.0515753	-1.1341079	-0.8448240
C	-4.6975433	-0.5701359	0.2588960
C	-3.9598543	0.1604161	1.1935390
C	-0.4655393	-0.0639939	-0.2798830
N	0.1258347	-0.8723489	-1.1670360
O	2.3352027	0.4796951	-0.7266430
C	1.5673937	1.2616721	-0.0638480
C	0.2382297	0.9950101	0.3182470
C	1.4281417	-1.0956399	-1.1104800
C	2.0082667	-1.8504899	0.0892070
O	1.4060347	-2.1045559	1.1090390
O	3.2716167	-2.2120979	-0.1697480
C	3.9508457	-2.9238409	0.8859070
H	-2.0248153	0.8862651	1.7685810
H	-2.1706893	-1.4036629	-1.8662350
H	-4.6185843	-1.7047889	-1.5751570
H	-5.7676943	-0.7028689	0.3920630
H	-4.4520673	0.5911821	2.0609390
H	-0.2787553	1.7756611	0.8580260
H	1.9378877	-1.3255389	-2.0449100
H	4.9428887	-3.1464029	0.4951980
H	4.0201047	-2.2980989	1.7788210
H	3.4148787	-3.8449349	1.1261400

O	1.4476967	3.6993489	-0.2982848
C	2.6832167	4.4301019	-0.3596788
H	2.3985627	5.4774039	-0.4612108
H	3.2659147	4.2793649	0.5529662
H	3.2772337	4.1115279	-1.2203388

C	3.4048467	2.7734481	-0.0732440
H	3.6016757	3.7446421	0.3799010
H	4.0804047	2.0168161	0.3327030
H	3.5266237	2.8297251	-1.1574580
O	2.0375597	2.4704601	0.2681660

Fig.1. Molecule **E** (R^1=Ph, R^2=H, R^3=MeO)
(PCM for CH_2Cl_2)

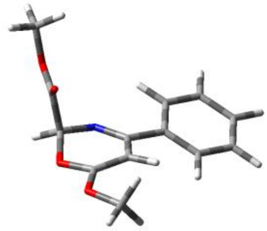

E = -858.83571749, **H (0K)** = -858.591267,
H (298K) = -858.573939, **G (298K)** = -858.637093 au.
Imaginary frequency = 0.

C	2.3711311	0.8444388	0.1578162
C	1.4688051	-0.1952672	0.4326952
C	1.9584211	-1.5098702	0.5150552
C	3.3110161	-1.7774662	0.3204302
C	4.2012911	-0.7344512	0.0457582
C	3.7277441	0.5764738	-0.0312388
C	0.0166091	0.0624928	0.6458422
N	-0.6564269	-0.8218842	1.3236612
O	-2.5003359	0.7900668	1.4205132
C	-1.8226879	1.5933648	0.5855632
C	-0.5814139	1.2773358	0.1260372
C	-2.0748619	-0.5962382	1.4219972
C	-2.8114769	-1.3271072	0.2757812
O	-3.3798439	-0.7797502	-0.6442668
O	-2.7175319	-2.6516772	0.4428432
C	-3.3293309	-3.4624782	-0.5833988
H	2.0224891	1.8713818	0.1150812
H	1.2597571	-2.3119282	0.7276832
H	3.6725361	-2.8004942	0.3794052
H	5.2567041	-0.9431852	-0.1065728
H	4.4139151	1.3937928	-0.2351728
H	-0.0461709	1.9323658	-0.5431788
H	-2.4440859	-0.9822702	2.3750152
H	-3.1664759	-4.4938832	-0.2734508
H	-4.3976339	-3.2447532	-0.6514138
H	-2.8547739	-3.2716322	-1.5486828
C	-1.9098939	3.7533408	-0.3818518
H	-2.6291609	4.5713328	-0.3814868
H	-0.9759059	4.0711698	0.0912502
H	-1.7193449	3.4241058	-1.4076308
O	-2.5263859	2.7015328	0.3777002

Fig.2. Molecule **D** (R^1= R^4=Ph, R^2=H, R^3=MeO)
(PCM for CH_2Cl_2)

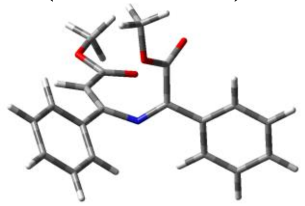

E = -1089.90527442, **H (0K)** = -1089.582404,
H (298K) = -1089.559066, **G (298K)** = -1089.637731 au.
Imaginary frequency = 0.

C	-1.2109531	0.4321879	-0.1706921
N	0.1048429	0.0110729	-0.2980651
O	0.5057859	2.8968319	-0.3870271
C	-0.7167131	2.8877429	-0.3959741
C	-1.6059031	1.7334479	-0.2668731
C	1.0339319	0.0777759	0.5757779
C	0.7890329	0.6602389	1.9660569
O	1.4897719	1.5086259	2.4754269
O	-0.2931821	0.1174849	2.5334809
C	-0.6557101	0.6389219	3.8317459
H	-1.5317271	0.0684939	4.1362499
H	0.1654849	0.4957299	4.5372729
H	-0.8934781	1.7021319	3.7550379
C	2.4037849	-0.3796201	0.2457819
C	2.7249789	-0.6814591	-1.0903711
C	3.3845349	-0.5530991	1.2370039
C	3.9928639	-1.1443731	-1.4246121
H	1.9687209	-0.5416231	-1.8552851
C	4.6532849	-1.0235561	0.8984429
H	3.1642289	-0.3187351	2.2720899
C	4.9617609	-1.3188931	-0.4301141
H	4.2294799	-1.3674241	-2.4610831
H	5.4006629	-1.1562141	1.6752719
H	5.9521269	-1.6806101	-0.6921581
O	-1.4519281	4.0236369	-0.5453301
C	-0.6960721	5.2360009	-0.6860161
H	-1.4344621	6.0328729	-0.7785791
H	-0.0637911	5.4024049	0.1903799
H	-0.0652281	5.1974039	-1.5782981
H	-2.6655621	1.9477579	-0.3280091
C	-2.2038871	-0.6733661	-0.0964901
C	-3.4575271	-0.4802001	0.5093119
C	-1.8967121	-1.9394471	-0.6241091
C	-4.3836151	-1.5197401	0.5684659
H	-3.6986051	0.4778969	0.9587689
C	-2.8272351	-2.9760591	-0.5667901
H	-0.9311881	-2.1001411	-1.0915131
C	-4.0741641	-2.7706651	0.0276469
H	-5.3450631	-1.3547201	1.0466959
H	-2.5772421	-3.9453781	-0.9891081
H	-4.7972901	-3.5800501	0.0753329

Fig.2. TS **D→E** (R^1= R^4=Ph, R^2=H, R^3=MeO)
(PCM for CH_2Cl_2)

Fig.2. Molecule **E** (R^1= R^4=Ph, R^2=H, R^3=MeO)
(PCM for CH_2Cl_2)

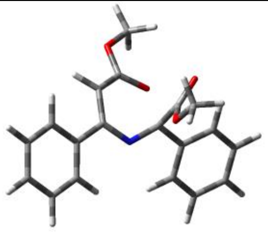

E = -1089.87985879, **H (0K)** = --1089.557441,
H (298K) = -1089.535288, **G (298K)** = -1089.609977 au.
Imaginary frequency = 1.

C	-1.3567085	-0.0826856	-0.3560859
N	-0.3362845	-0.9407536	-0.2830379
O	1.2263545	1.0905814	-0.6893969
C	0.1476315	1.7636774	-0.8179269
C	-1.1466505	1.2994794	-0.5116029
C	0.8588505	-0.5005166	0.1384651
C	0.9265035	0.0663814	1.5878811
O	1.6633405	0.9493004	1.9709701
O	0.0970835	-0.6208866	2.3816181
C	0.1067025	-0.2382536	3.7740751
H	-0.6020215	-0.9065056	4.2612361
H	1.1077085	-0.3627996	4.1932271
H	-0.2077865	0.8023784	3.8810231
C	2.0628775	-1.3089586	-0.2493899
C	1.8961805	-2.4604736	-1.0318779
C	3.3542455	-0.9286006	0.1473251
C	3.0017545	-3.2205046	-1.4096539
H	0.8949925	-2.7493536	-1.3307699
C	4.4582525	-1.6885726	-0.2378929
H	3.4913705	-0.0343686	0.7445561
C	4.2859805	-2.8368116	-1.0145759
H	2.8603275	-4.1137216	-2.0118629
H	5.4542085	-1.3828226	0.0701991
H	5.1473155	-3.4300486	-1.3093069
O	0.2265765	2.9935524	-1.3384419
C	1.5416855	3.5104514	-1.6269639
H	1.3816765	4.5494204	-1.9137589
H	2.1805315	3.4487094	-0.7431359
H	1.9989745	2.9564224	-2.4500659
H	-1.9642285	1.9766644	-0.7141629
C	-2.7261565	-0.6627686	-0.3869679
C	-3.8686995	0.1279684	-0.1773739
C	-2.8946335	-2.0403066	-0.6090789
C	-5.1413385	-0.4414086	-0.1971099
H	-3.7696755	1.1891484	0.0261471
C	-4.1670335	-2.6066396	-0.6384939
H	-2.0149435	-2.6553636	-0.7625879
C	-5.2963005	-1.8093436	-0.4332129
H	-6.0122145	0.1847944	-0.0245569
H	-4.2780335	-3.6721116	-0.8206589
H	-6.2887815	-2.2511296	-0.4528849

Fig.2. Molecule **D** (R^1=R^3=Me, R^2=R^4=Ph)
(PCM for CH_2Cl_2)

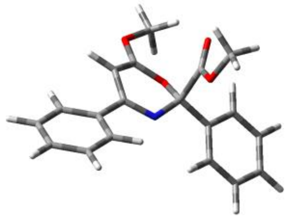

E = -1089.88950085, **H (0K)** = -1089.565365,
H (298K) = -1089.543121, **G (298K)** = -1089.617576 au.
Imaginary frequency = 0.

C	-1.3760007	0.6772114	0.0579005
N	-0.3889307	-0.1768256	0.0892285
O	1.1143753	1.6647904	-0.4143365
C	0.0961823	2.5229374	-0.2790735
C	-1.1623147	2.1042344	0.0398705
C	0.9131003	0.3842534	0.2860945
C	1.1805213	0.7027474	1.7954615
O	1.7484343	1.6999164	2.1910165
O	0.7558363	-0.2923726	2.5778615
C	1.0207833	-0.1392826	3.9893945
H	0.6241153	-1.0395116	4.4568215
H	2.0957113	-0.0547636	4.1649755
H	0.5156563	0.7492704	4.3745115
C	2.0287093	-0.5283096	-0.2165465
C	1.7459173	-1.8193376	-0.6707855
C	3.3550333	-0.0738336	-0.2057775
C	2.7784853	-2.6445036	-1.1219805
H	0.7178663	-2.1614106	-0.6693975
C	4.3835763	-0.8994676	-0.6587905
H	3.5799823	0.9256814	0.1531235
C	4.0977383	-2.1879096	-1.1185645
H	2.5491903	-3.6456036	-1.4770995
H	5.4078253	-0.5369206	-0.6512055
H	4.8996033	-2.8317446	-1.4696455
O	0.3770453	3.7914324	-0.5520315
C	1.7649193	4.1874764	-0.6404575
H	1.7416213	5.2750874	-0.6995685
H	2.3079033	3.8604304	0.2483035
H	2.2263523	3.7709584	-1.5384255
H	-1.9608407	2.8251074	0.1362205
C	-2.7597867	0.1293224	0.0099005
C	-3.8309937	0.8754424	-0.5068045
C	-3.0084297	-1.1678526	0.4893675
C	-5.1180947	0.3376324	-0.5439955
H	-3.6587247	1.8710554	-0.9034195
C	-4.2942117	-1.7019846	0.4570515
H	-2.1800857	-1.7396976	0.8933515
C	-5.3542027	-0.9506436	-0.0601655
H	-5.9349607	0.9248274	-0.9542905
H	-4.4723957	-2.7035856	0.8389985
H	-6.3574687	-1.3672886	-0.0848735

Fig.2. TS **D→E** (R^1=R^3=Me, R^2=R^4=Ph)
(PCM for CH_2Cl_2)

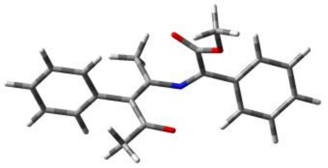

E = -1053.98492408, **H (0K)** = -1053.640215,
H (298K) = -1053.616113, **G (298K)** = -1053.695751

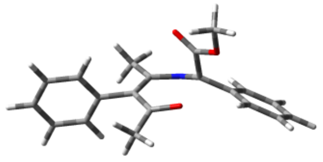

E = -1053.96740717, **H (0K)** = -1053.623361,
H (298K) = -1053.600183, **G (298K)** = -1053.677144

au.
Imaginary frequency = 0.

C	-0.6997460	-0.3730950	-0.8042040
N	0.6429700	-0.6480110	-0.6370240
O	-0.0638170	-1.1068130	1.8777300
C	-1.2362800	-0.8945710	1.5611030
C	-1.6372040	-0.5193630	0.1859250
C	1.6136960	0.1420830	-0.3934310
C	-1.0426860	-0.0865350	-2.2491320
H	-0.6804890	-0.9036230	-2.8834180
H	-2.1169020	0.0247590	-2.3937240
H	-0.5386500	0.8312410	-2.5670250
C	-2.3307250	-1.0294650	2.6103450
H	-2.9133180	-0.1051140	2.6931720
H	-3.0372450	-1.8234620	2.3433270
H	-1.8701030	-1.2623280	3.5725910
C	-3.0911020	-0.3043780	-0.1133170
C	-3.6365510	0.9896460	-0.1107030
C	-3.9432030	-1.3870000	-0.3853270
C	-4.9927800	1.1963520	-0.3716410
H	-2.9857510	1.8362040	0.0926860
C	-5.2999140	-1.1833940	-0.6450310
H	-3.5352620	-2.3947400	-0.3976930
C	-5.8288520	0.1095670	-0.6378480
H	-5.3953140	2.2059220	-0.3675120
H	-5.9425130	-2.0342050	-0.8559650
H	-6.8844970	0.2690270	-0.8405080
C	1.3364200	1.6131670	-0.0983980
O	0.3911410	2.2230740	-0.5574170
O	2.2173920	2.1258840	0.7674190
C	1.9904190	3.4978880	1.1615250
H	2.7941100	3.7339270	1.8571890
H	2.0284940	4.1523660	0.2880060
H	1.0171150	3.5914830	1.6480340
C	2.9949890	-0.3966790	-0.3308840
C	3.1844270	-1.7394720	0.0374240
C	4.1117190	0.3811830	-0.6773640
C	4.4632240	-2.2897790	0.0626250
H	2.3187260	-2.3303430	0.3168650
C	5.3894250	-0.1774310	-0.6644480
H	3.9852210	1.4194210	-0.9656860
C	5.5693120	-1.5113490	-0.2917390
H	4.5988920	-3.3252240	0.3622390
H	6.2446500	0.4310340	-0.9444370
H	6.5667950	-1.9416920	-0.2736970

au.
Imaginary frequency = 0.

C	-0.4905948	-0.7116888	-1.0026704
N	0.8350465	-0.8089890	-1.0266378
O	0.7452000	0.3518301	1.3737097
C	-0.5096707	0.1055374	1.3138614
C	-1.2059607	-0.2526139	0.1303076
C	1.6527462	-0.0189986	-0.3567155
C	-1.2129520	-1.2967435	-2.1905886
H	-0.4898229	-1.6250925	-2.9398639
H	-1.8448210	-2.1439666	-1.9042418
H	-1.8738661	-0.5433990	-2.6335683
C	-1.2402296	0.1896133	2.6400209
H	-2.0443959	0.9310493	2.5801193
H	-1.7045473	-0.7666750	2.9021369
H	-0.5389389	0.4820773	3.4238147
C	-2.6994865	-0.3833751	0.1668343
C	-3.5121190	0.7113256	-0.1695453
C	-3.3249349	-1.5883745	0.5284312
C	-4.9048831	0.6073554	-0.1445312
H	-3.0422303	1.6495613	-0.4535959
C	-4.7167416	-1.6962618	0.5547958
H	-2.7113043	-2.4467642	0.7915396
C	-5.5111122	-0.5974174	0.2180900
H	-5.5152258	1.4670422	-0.4087637
H	-5.1801789	-2.6377376	0.8381260
H	-6.5945189	-0.6799184	0.2379963
C	1.5433335	1.4854783	-0.6723849
O	0.7137600	1.9434280	-1.4287514
O	2.4657515	2.2136541	-0.0360227
C	2.4017244	3.6383040	-0.2567836
H	3.2292713	4.0564009	0.3148220
H	2.5143013	3.8615068	-1.3200078
H	1.4478439	4.0328688	0.1010494
C	2.9720449	-0.5953940	0.0506902
C	3.4916352	-1.6664179	-0.6941806
C	3.6928097	-0.1217030	1.1585107
C	4.7142173	-2.2414257	-0.3491236
H	2.9305459	-2.0350165	-1.5457701
C	4.9067704	-0.7102173	1.5086093
H	3.2895430	0.6871072	1.7537249
C	5.4256158	-1.7661656	0.7541641
H	5.1082657	-3.0624111	-0.9417658
H	5.4486516	-0.3431896	2.3759355
H	6.3762364	-2.2162889	1.0269219

Fig.2. Molecule **E** (R^1=R^3=Me, R^2=R^4=Ph)
(PCM for CH_2Cl_2)

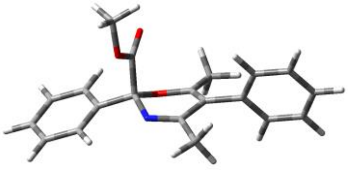

E = -1053.99147530, **H (0K)** = -1053.644704,
H (298K) = -1053.621854, **G (298K)** = -1053.697302
au.
Imaginary frequency = 0.

C	0.4374276	-0.1421081	1.4438814
N	-0.8504204	-0.0354981	1.4616894
O	-0.8819024	-1.0289101	-0.7364566
C	0.4741656	-0.9584311	-0.8115826
C	1.1978376	-0.4463901	0.2202784

Fig.2. Molecule **D** (R^1=R^3=R^4=Ph, R^2=H)
(PCM for CH_2Cl_2)

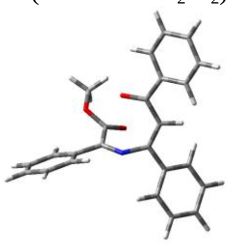

E = -1206.41415286, **H (0K)** = -1206.043040,
H (298K) = -1206.017852, **G (298K)** = -1206.100730
au.
Imaginary frequency = 0.

C	0.1619650	1.2281730	-0.1650270
N	-0.9351950	0.4670420	-0.5257820
O	1.0454720	-1.5807150	0.1317530

C	-1.5042124	-0.0990841	0.1762464
C	1.1701836	-0.0227841	2.7552984
H	0.4530876	0.1260729	3.5648774
H	1.7678036	-0.9195521	2.9552924
H	1.8678446	0.8222329	2.7320044
C	0.9774706	-1.5345561	-2.0945436
H	2.0664096	-1.5509841	-2.1330326
H	0.5973716	-2.5556581	-2.2182516
H	0.5916176	-0.9415011	-2.9321196
C	2.6876796	-0.3507131	0.1950714
C	3.3147936	0.9044619	0.1300994
C	3.4945646	-1.4984901	0.2567044
C	4.7065626	1.0093069	0.1220474
H	2.7038666	1.8021009	0.0774374
C	4.8871376	-1.3957441	0.2440184
H	3.0241006	-2.4763021	0.3184874
C	5.4970686	-0.1412221	0.1777824
H	5.1730026	1.9893389	0.0676104
H	5.4942506	-2.2959001	0.2902594
H	6.5806106	-0.0602961	0.1701044
C	-1.4815704	1.2957859	-0.5316776
O	-1.2733444	1.4526679	-1.7162096
O	-1.7793254	2.2772019	0.3263704
C	-1.8682804	3.6022179	-0.2398126
H	-2.1247184	4.2560559	0.5927784
H	-0.9102994	3.8932209	-0.6766746
H	-2.6445794	3.6338779	-1.0079036
C	-2.9645684	-0.5325781	0.2949884
C	-3.6069024	-0.5623491	1.5360044
C	-3.6801934	-0.8694721	-0.8625226
C	-4.9487024	-0.9410371	1.6209574
H	-3.0467894	-0.2948641	2.4242944
C	-5.0194004	-1.2488911	-0.7739566
H	-3.1854414	-0.8392621	-1.8280646
C	-5.6575894	-1.2860991	0.4688284
H	-5.4387954	-0.9668401	2.5905734
H	-5.5644264	-1.5141891	-1.6757946
H	-6.7013494	-1.5806401	0.5371414

C	1.8397690	-0.6542840	-0.0900030
C	1.4437020	0.7512770	-0.0878710
C	-1.5898960	-0.3896220	0.1556740
C	-1.2224110	-0.6295680	1.6189480
O	-0.7990380	0.2462320	2.3438180
O	-1.4071080	-1.9009170	1.9847930
C	-0.9885640	-2.2330360	3.3263390
H	2.2325850	1.4915090	-0.0465920
H	-1.2144700	-3.2917030	3.4451210
H	0.0830240	-2.0519350	3.4355200
H	-1.5395080	-1.6348530	4.0553580
C	3.2870260	-0.9793680	-0.3433730
C	3.7507030	-2.2530630	0.0252460
C	4.1808410	-0.0842410	-0.9532000
C	5.0765040	-2.6179800	-0.1928400
H	3.0518410	-2.9438930	0.4852740
C	5.5074070	-0.4527980	-1.1809300
H	3.8413030	0.8943190	-1.2770280
C	5.9595920	-1.7169990	-0.7966410
H	5.4238540	-3.6033050	0.1058770
H	6.1860480	0.2464110	-1.6614560
H	6.9941950	-2.0008180	-0.9693670
C	-0.1186070	2.6868020	-0.0920620
C	-1.1887910	3.2478420	-0.8106590
C	0.6702610	3.5272050	0.7126410
C	-1.4476980	4.6158890	-0.7422590
H	-1.8037210	2.6064810	-1.4328740
C	0.4066700	4.8937970	0.7809710
H	1.4724140	3.1036270	1.3087090
C	-0.6506590	5.4442030	0.0512800
H	-2.2719550	5.0355680	-1.3122900
H	1.0210650	5.5278780	1.4140180
H	-0.8552580	6.5097970	0.1063860
C	-2.7127900	-1.1221400	-0.4782980
C	-3.7857840	-1.6311190	0.2723410
C	-2.7315790	-1.2657460	-1.8771100
C	-4.8536710	-2.2631590	-0.3642580
H	-3.7947400	-1.5294290	1.3516300
C	-3.7919600	-1.9092610	-2.5076990
H	-1.9012640	-0.8747600	-2.4557170
C	-4.8584010	-2.4082610	-1.7527280
H	-5.6813100	-2.6439300	0.2271700
H	-3.7871740	-2.0247510	-3.5878240
H	-5.6869450	-2.9095490	-2.2452490

Fig.2. TS **D→E** ($R^1=R^3=R^4=Ph$, $R^2=H$)
(PCM for CH_2Cl_2)

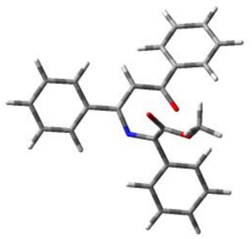

E = -1206.39756893, **H (0K)** = -1206.027170,
H (298K) = -1206.002908, **G (298K)** = --1206.082683
au.
Imaginary frequency = 1.

C	-1.5136920	0.2747050	0.0107990
N	-1.0008820	-0.9387890	-0.1750750
O	1.3735670	0.2502850	0.0976920
C	0.7019280	1.3462270	-0.0098580

Fig.2. Molecule **E** ($R^1=R^3=R^4=Ph$, $R^2=H$)
(PCM for CH_2Cl_2)

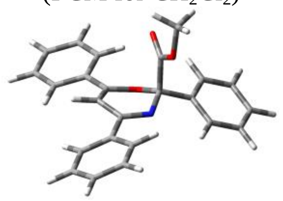

E = -1206,41837726, **H (0K)** = -1206,045999,
H (298K) = -1206,021741, **G (298K)** = -1206,101661
au.
Imaginary frequency = 0.

C	-1.2071772	0.8494862	0.2391267
N	-0.9762422	-0.4249838	0.1307277
O	1.3783478	0.0927192	-0.1058463
C	1.1355008	1.3987002	0.1775477
C	-0.1339182	1.8063222	0.4448587

C	-0.6952460	1.4201600	0.1176490
C	0.1899220	-1.3040260	0.2743090
C	0.3778560	-1.2268510	1.8033790
O	-0.4556960	-0.7597570	2.5491960
O	1.5442710	-1.7420810	2.2042170
C	1.8044680	-1.6733620	3.6220250
H	-1.1597590	2.3937130	0.0616890
H	2.7735740	-2.1507510	3.7610980
H	1.8370390	-0.6316410	3.9495090
H	1.0279050	-2.2064350	4.1749020
C	1.5100030	2.5633460	-0.3099520
C	2.8764530	2.5663380	0.0198500
C	0.9567360	3.7038120	-0.9172750
C	3.6656620	3.6853250	-0.2363160
H	3.3052120	1.6860800	0.4863910
C	1.7487990	4.8214370	-1.1752830
H	-0.0867020	3.7115330	-1.2145550
C	3.1036850	4.8170680	-0.8332740
H	4.7184140	3.6765030	0.0319910
H	1.3096460	5.6930250	-1.6523700
H	3.7186980	5.6899270	-1.0342090
C	-2.9900670	0.4046630	-0.0956800
C	-3.7428880	-0.6002760	-0.7268090
C	-3.6636230	1.5099560	0.4512700
C	-5.1283660	-0.4943920	-0.8231300
H	-3.2262440	-1.4559200	-1.1479480
C	-5.0515760	1.6092960	0.3634570
H	-3.1073490	2.2812920	0.9743000
C	-5.7881090	0.6111460	-0.2786400
H	-5.6948200	-1.2747010	-1.3239040
H	-5.5578160	2.4649930	0.8012530
H	-6.8691940	0.6919970	-0.3508660
C	0.9064960	-2.3682810	-0.4984510
C	2.3049680	-2.4909810	-0.4901300
C	0.1482450	-3.2626180	-1.2703990
C	2.9261350	-3.4872090	-1.2410780
H	2.9002000	-1.7935640	0.0845380
C	0.7735750	-4.2671860	-2.0086650
H	-0.9317300	-3.1673040	-1.2779050
C	2.1647190	-4.3820350	-1.9980150
H	4.0099900	-3.5634050	-1.2361790
H	0.1720110	-4.9576970	-2.5933290
H	2.6534660	-5.1612850	-2.5764280

C	0.3766228	-0.8406508	0.3891957
C	0.6423168	-0.9826698	1.9245627
O	1.6944218	-0.6984408	2.4573757
O	-0.4104182	-1.5139938	2.5531077
C	-0.2382062	-1.7641348	3.9649567
H	-0.3366442	2.8350882	0.7069247
H	-1.1806382	-2.1961428	4.2989617
H	0.5855558	-2.4631858	4.1272787
H	-0.0343112	-0.8297298	4.4924967
C	2.3314218	2.2512282	0.1087377
C	3.6020248	1.6951302	0.3374667
C	2.2259108	3.6242032	-0.1765053
C	4.7396668	2.4987332	0.2898627
H	3.6820988	0.6405172	0.5742537
C	3.3653448	4.4234472	-0.2191493
H	1.2541588	4.0594262	-0.3881723
C	4.6253088	3.8632942	0.0130937
H	5.7162128	2.0602452	0.4747647
H	3.2723748	5.4816842	-0.4457663
H	5.5134708	4.4877892	-0.0253543
C	-2.6129432	1.3184252	0.1032577
C	-3.6689962	0.4275762	0.3609977
C	-2.9169052	2.6340822	-0.2816243
C	-4.9924792	0.8431952	0.2401217
H	-3.4321192	-0.5860418	0.6662177
C	-4.2435462	3.0477492	-0.4086273
H	-2.1186682	3.3341412	-0.5071433
C	-5.2845132	2.1553292	-0.1458763
H	-5.7985052	0.1457382	0.4511937
H	-4.4622372	4.0665932	-0.7159473
H	-6.3173742	2.4794182	-0.2396433
C	0.6990378	-2.1822198	-0.2689663
C	2.0047888	-2.6889868	-0.2055073
C	-0.2942062	-2.9190578	-0.9202883
C	2.3129868	-3.9124368	-0.7997223
H	2.7774768	-2.1255848	0.3078017
C	0.0170948	-4.1448378	-1.5131513
H	-1.3013232	-2.5215118	-0.9594813
C	1.3193308	-4.6442488	-1.4557443
H	3.3288478	-4.2946778	-0.7488603
H	-0.7611142	-4.7084518	-2.0207853
H	1.5596098	-5.5984368	-1.9168593

Fig.2. Molecule **D** (R^1=Ph,R^2=H, R^3=MeO, R^4=CO_2Me) (PCM for CH_2Cl_2)

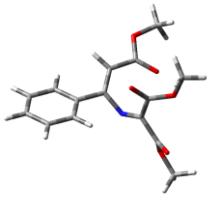

E = -1086.71380299, **H (0K)** = -1086.429380, **H (298K)** = -1086.406034, **G (298K)** = -1086.484311 au.

Imaginary frequency = 0.

C	-3.5050680	0.6534810	-0.5612030
C	-2.3739300	-0.0613190	-0.1321860
C	-2.5540700	-1.3110370	0.4826430
C	-3.8353450	-1.8220560	0.6839570
C	-4.9542930	-1.1004360	0.2623200
C	-4.7844280	0.1374900	-0.3625700
C	-1.0038180	0.4892300	-0.3230880

Fig.2. TS **D→E** (R^1=Ph,R^2=H, R^3=MeO, R^4=CO_2Me) (PCM for CH_2Cl_2)

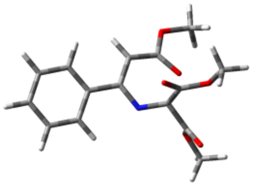

E = -1086.69120224, **H (0K)** = --1086.407071, **H (298K)** = -1086.384914, **G (298K)** = -1086.459224 au.

Imaginary frequency = 1.

C	-3.5650180	1.0211160	0.1947810
C	-2.6274170	0.0156410	-0.0960690
C	-3.0849580	-1.2992350	-0.2887030
C	-4.4431970	-1.5970260	-0.2086250
C	-5.3676050	-0.5886000	0.0771560
C	-4.9229880	0.7196320	0.2835400
C	-1.1754330	0.3071770	-0.1794350

N	-0.0575480	-0.4610300	-0.7180410
O	1.6339390	1.9086700	-0.5985740
C	0.5464740	2.4595120	-0.5020610
C	-0.7571220	1.8242580	-0.2884270
C	0.9059710	-0.9099190	-0.0278020
C	1.1751960	-0.5142760	1.4173290
O	0.2919630	-0.3289670	2.2274160
O	2.4798490	-0.3763290	1.6413510
C	2.8541310	0.0408540	2.9746550
H	-3.3823500	1.6023870	-1.0735250
H	-1.6918790	-1.8710270	0.8281390
H	-3.9585630	-2.7846600	1.1720130
H	-5.9520660	-1.5023320	0.4142850
H	-5.6484980	0.6991390	-0.7060150
H	-1.5865500	2.4950560	-0.1024800
H	3.9409200	0.1024160	2.9616440
H	2.4128950	1.0141860	3.1991450
H	2.5156750	-0.6966170	3.7054110
C	1.6135600	4.5558200	-0.7493590
H	1.3107790	5.6028710	-0.7614890
H	2.3093090	4.3689850	0.0729130
H	2.0943110	4.2873970	-1.6937350
O	0.4009480	3.8063740	-0.5672490
C	1.7925430	-1.9907780	-0.6086020
O	2.2652800	-2.8679930	0.0862680
O	1.9454580	-1.8763690	-1.9258620
C	2.7353800	-2.9105510	-2.5556870
H	2.7604380	-2.6486430	-3.6122070
H	3.7432760	-2.9216300	-2.1351100
H	2.2645650	-3.8850480	-2.4090170

Fig.2. Molecule **E** (R^1=Ph,R^2=H, R^3=MeO, R^4=CO_2Me) (PCM for CH_2Cl_2)

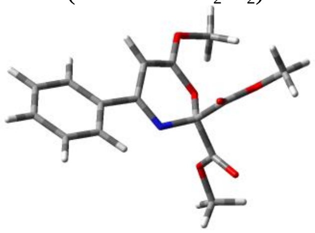

E = -1086.70221980, **H (0K)** = -1086.416492, **H (298K)** = -1086.394204, **G (298K)** = -1086.469117 au.

Imaginary frequency = 0.

C	-3.4965622	1.3459178	-0.3447187
C	-2.5658342	0.4123208	0.1384583
C	-3.0376522	-0.7826982	0.7076823
C	-4.4043512	-1.0339282	0.7963443
C	-5.3234602	-0.0972442	0.3131433
C	-4.8656942	1.0910628	-0.2594817
C	-1.1001582	0.6552148	0.0589973
N	-0.3115392	-0.3863792	0.1030133
O	1.5093578	1.0572968	-0.6253837
C	0.6979848	2.1199928	-0.5112807
C	-0.5932732	1.9978238	-0.0890667
C	1.0812528	-0.0909492	0.1730653
C	1.5334308	0.1531598	1.6530003
O	0.8630628	-0.1136362	2.6218073
O	2.7660308	0.6751078	1.6874913
C	3.3165878	0.8932818	3.0057693
H	-3.1555102	2.2650488	-0.8104407
H	-2.3162052	-1.4995462	1.0842343
H	-4.7548282	-1.9585482	1.2466933

N	-0.3401360	-0.7417630	-0.2008940
O	1.6011240	0.9503700	-0.5965330
C	0.6590190	1.8101290	-0.7191420
C	-0.6943480	1.6096350	-0.3794910
C	0.9274320	-0.5819410	0.1496370
C	1.2352210	-0.1840540	1.6146390
O	0.3828870	-0.1032680	2.4701920
O	2.5374070	0.0384380	1.7996620
C	2.9304090	0.3903820	3.1439690
H	-3.2359280	2.0385820	0.3788900
H	-2.3623350	-2.0778650	-0.5070660
H	-4.7812300	-2.6171830	-0.3680310
H	-6.4268380	-0.8212980	0.1431060
H	-5.6336630	1.5069780	0.5182420
H	-1.3658840	2.4367940	-0.5598440
H	4.0117870	0.5134850	3.1057560
H	2.4462010	1.3217110	3.4463280
H	2.6563500	-0.4078710	3.8372670
C	2.3391170	3.2527510	-1.5888170
H	2.3652900	4.2974940	-1.8964000
H	2.9797020	3.0912300	-0.7189590
H	2.6615170	2.6070600	-2.4086180
O	0.9585390	2.9949570	-1.2569350
C	1.8654650	-1.6590400	-0.3965930
O	2.3481270	-2.5123170	0.3181180
O	2.0089120	-1.5812810	-1.7163130
C	2.8157740	-2.6181770	-2.3199030
H	2.8313020	-2.3869480	-3.3838480
H	3.8257700	-2.5986710	-1.9047600
H	2.3641050	-3.5966990	-2.1429750

Fig.2. Molecule **D** (R^1=R^3=Me,R^2= Ph, R^4=CO_2Me) (PCM for CH_2Cl_2)

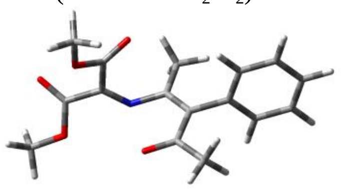

E = -1050.79561378, **H (0K)** = -1050.489271, **H (298K)** = --1050.465180, **G (298K)** = -1050.544984 au.

Imaginary frequency = 0.

C	0.3607620	-0.4358850	-0.7735310
N	-0.9747350	-0.7806330	-0.6011270
O	-0.2749550	-1.0805860	1.9045180
C	0.8970940	-0.8537970	1.6034430
C	1.2981090	-0.5040810	0.2184220
C	-1.9649010	-0.0078500	-0.4246690
C	-1.8179410	1.4813850	-0.1606320
O	-0.9051720	2.1510810	-0.5995010
O	-2.7702430	1.9220240	0.6616800
C	-2.6931200	3.3174210	1.0327660
H	-3.5299590	3.4812070	1.7095540
H	-1.7435460	3.5202630	1.5324930
H	-2.7845450	3.9457080	0.1441910
C	0.6781090	-0.1605260	-2.2254930
H	0.1611390	0.7503240	-2.5437550
H	1.7497760	-0.0381230	-2.3796100
H	0.3184840	-0.9862510	-2.8492980
C	1.9832440	-0.9382050	2.6626830
H	2.7236000	-1.7042180	2.4060790
H	2.5265020	0.0101940	2.7419540

H	-6.3899612	-0.2930522	0.3833213
H	-5.5735292	1.8199598	-0.6441377
H	-1.2148762	2.8773378	-0.0098527
H	4.3090448	1.3099088	2.8398403
H	2.6915758	1.5939238	3.5636823
H	3.3800108	-0.0534292	3.5466563
C	2.6349428	3.3454588	-1.1798837
H	2.8383528	4.4057388	-1.3239277
H	3.2084688	2.9539858	-0.3373747
H	2.8751108	2.7876738	-2.0875337
O	1.2169618	3.2753718	-0.9037527
C	1.8607688	-1.3094132	-0.3636697
O	2.2908798	-2.1749912	0.3686183
O	1.9376038	-1.3093092	-1.6916687
C	2.5894608	-2.4575292	-2.2817647
H	2.5684038	-2.2791732	-3.3557397
H	3.6176328	-2.5326272	-1.9215017
H	2.0432948	-3.3689962	-2.0298147

H	1.5243100	-1.1812820	3.6230090
C	2.7400670	-0.2239050	-0.0795720
C	3.6418100	-1.2683970	-0.3390080
C	3.2220950	1.0947100	-0.0849790
C	4.9884110	-1.0025210	-0.5947930
H	3.2814260	-2.2940570	-0.3444210
C	4.5683040	1.3626150	-0.3414670
H	2.5313530	1.9112630	0.1087810
C	5.4554410	0.3140780	-0.5953540
H	5.6714180	-1.8234990	-0.7962020
H	4.9229130	2.3898980	-0.3437900
H	6.5032840	0.5214510	-0.7947740
C	-3.3650060	-0.5704400	-0.5125400
O	-4.2868280	0.0649680	-0.9843720
O	-3.4413740	-1.8233150	-0.0628550
C	-4.7378210	-2.4502750	-0.1787050
H	-4.6150890	-3.4443990	0.2484040
H	-5.4839020	-1.8792970	0.3784360
H	-5.0333830	-2.5141070	-1.2283590

Fig.2. . TS **D→E** (R^1=R^3=Me,R^2= Ph, R^4=CO_2Me) (PCM for CH_2Cl_2)

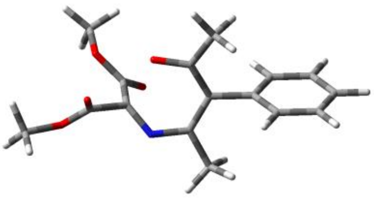

E = -1050.77877457, **H (0K)** = -1050.473100,
H (298K) = -1050.449889, **G (298K)** = -1050.526858 au.
Imaginary frequency = 1.

C	0.6866440	-0.7087330	-0.7091374
N	-0.6226220	-1.0001610	-0.5868484
O	-0.5081060	0.3193750	1.7185076
C	0.7511160	0.2087000	1.5701066
C	1.4033710	-0.0965510	0.3313276
C	-1.4476590	-0.2167230	0.0519176
C	-1.5691160	1.2630430	-0.3208064
O	-0.9039340	1.8004450	-1.1781914
O	-2.5093870	1.8584700	0.4180806
C	-2.7039990	3.2692340	0.1790836
H	-3.5154880	3.5654100	0.8420466
H	-1.7908350	3.8190430	0.4179826
H	-2.9744420	3.4403900	-0.8651014
C	1.3711050	-1.2539310	-1.9350934
H	1.8732350	-0.4417440	-2.4723574
H	2.1404240	-1.9892520	-1.6782074
H	0.6362920	-1.7195580	-2.5948194
C	1.5693940	0.3912470	2.8316306
H	2.2244470	-0.4660420	3.0142596
H	2.2141490	1.2716110	2.7297046
H	0.8995080	0.5346320	3.6811616
C	2.8977270	0.0000430	0.2537166
C	3.7247170	-1.0972570	0.5458096
C	3.5025790	1.2105130	-0.1199674
C	5.1142870	-0.9879310	0.4670876
H	3.2725140	-2.0417230	0.8389306
C	4.8925430	1.3228740	-0.1992864
H	2.8739510	2.0665730	-0.3515184
C	5.7020430	0.2234470	0.0944076
H	5.7371680	-1.8481580	0.6977296
H	5.3416540	2.2685310	-0.4910974

Fig.2. Molecule **E** (R^1=R^3=Me,R^2= Ph, R^4=CO_2Me) (PCM for CH_2Cl_2)

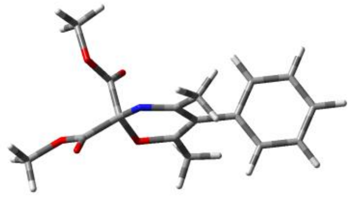

E = -1050.80637090, **H (0K)** = -1050.498140,
H (298K) = -1050.475167, **G (298K)** = -1050.551268 au.
Imaginary frequency = 0.

C	0.3652263	-0.3101171	1.3107300
N	-0.9240787	-0.3001191	1.4034050
O	-1.0017597	-1.5301731	-0.7014880
C	0.3490163	-1.4001661	-0.8312550
C	1.0866023	-0.7527731	0.1059570
C	-1.6594477	-0.6360341	0.2152020
C	-2.9606217	-1.3899441	0.5938690
O	-3.2039927	-1.8481481	1.6845080
O	-3.7555227	-1.4855161	-0.4794840
C	-4.9911927	-2.2086841	-0.2900240
H	-5.4968187	-2.1754191	-1.2539730
H	-4.7819317	-3.2405531	0.0005270
H	-5.5955327	-1.7252021	0.4806450
C	1.1512083	0.0977989	2.5292380
H	1.8343063	-0.7013211	2.8388710
H	1.7709213	0.9753069	2.3118720
H	0.4679213	0.3321309	3.3474480
C	0.8287253	-2.0802231	-2.0711660
H	0.3790003	-1.5988481	-2.9476050
H	1.9145723	-2.0436871	-2.1555100
H	0.5019853	-3.1268571	-2.0724120
C	2.5678093	-0.5959981	-0.0059140
C	3.1255703	0.6521959	-0.3270520
C	3.4332543	-1.6777571	0.2229370
C	4.5088903	0.8139759	-0.4199200
H	2.4671773	1.4970509	-0.5123660
C	4.8172543	-1.5184351	0.1263840
H	3.0153753	-2.6478131	0.4791990
C	5.3586223	-0.2716191	-0.1939020
H	4.9222263	1.7866069	-0.6730560
H	5.4712793	-2.3678251	0.3045460

H	6.7835600	0.3093890	0.0331916
C	-2.7088460	-0.8909690	0.5922166
O	-2.7877530	-1.4762960	1.6462186
O	-3.6900730	-0.7809190	-0.3118544
C	-4.9385770	-1.4264520	0.0275856
H	-5.5997150	-1.2373620	-0.8167804
H	-4.7824140	-2.4986980	0.1639196
H	-5.3472060	-0.9939180	0.9433816

H	6.4354883	-0.1465361	-0.2673460
C	-2.0020367	0.6671709	-0.5607970
O	-1.5508147	0.9537949	-1.6466980
O	-2.8319687	1.4279659	0.1599980
C	-3.2091667	2.6917329	-0.4303790
H	-3.8837407	3.1560379	0.2874330
H	-2.3245327	3.3131149	-0.5859450
H	-3.7145857	2.5256119	-1.3842930

Fig.2. Molecule **D** (R^1=R^3=Ph,R^2=H, R^4=CO_2Me)
(PCM for CH_2Cl_2)

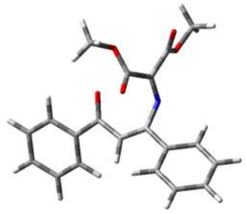

E = -1203.22421965, **H (0K)** = -1202.891627,
H (298K) = -1202.866403, **G (298K)** = -1202.949425 au.
Imaginary frequency = 0.

C	-0.0967390	1.2176422	0.0029372
N	-0.8471180	0.1537512	-0.4903718
O	1.6659090	-1.1520288	0.2502832
C	2.1112170	-0.0047738	0.1051232
C	1.2606110	1.1869922	0.1528752
C	-1.1719680	-0.9131588	0.1093672
C	-2.0636360	-1.9074798	-0.6041618
O	-2.9639810	-2.4874258	-0.0321488
O	-1.7635730	-2.0239078	-1.8971478
C	-2.6097530	-2.9163318	-2.6570878
H	-2.2118940	-2.8955328	-3.6704128
H	-2.5600000	-3.9254138	-2.2421588
H	-3.6425900	-2.5618138	-2.6389388
C	-0.8327530	-1.2001338	1.5648172
O	-0.7377410	-0.3294448	2.4025372
O	-0.6591230	-2.5042398	1.7712332
C	-0.2636470	-2.8825948	3.1079392
H	-0.1757460	-3.9676318	3.0864492
H	0.6948710	-2.4211688	3.3555972
H	-1.0226480	-2.5693758	3.8279322
H	1.7496240	2.1431712	0.2904352
C	-0.8684670	2.4792742	0.1290902
C	-2.0104700	2.7070922	-0.6587498
C	-0.4745380	3.4605032	1.0555942
C	-2.7252730	3.8975072	-0.5373498
H	-2.3215390	1.9553352	-1.3761678
C	-1.1939450	4.6474452	1.1753562
H	0.3788500	3.2793102	1.7013752
C	-2.3193400	4.8719122	0.3774742
H	-3.5991210	4.0648222	-1.1606738
H	-0.8819080	5.3933282	1.9008222
H	-2.8797460	5.7976042	0.4735892
C	3.5866600	0.1898722	-0.0961368
C	4.1396220	1.3684882	-0.6229448
C	4.4432520	-0.8736108	0.2338912
C	5.5179420	1.4811782	-0.8085008
H	3.4975530	2.1931172	-0.9153448
C	5.8192440	-0.7574048	0.0583952
H	4.0079330	-1.7845828	0.6311672
C	6.3604240	0.4221442	-0.4634608
H	5.9326470	2.3946332	-1.2253438

Fig.2. TS **D→E** (R^1=R^3=Ph,R^2=H, R^4=CO_2Me)
(PCM for CH_2Cl_2)

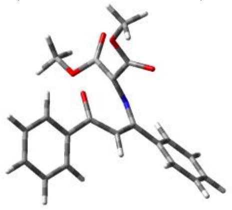

E = -1203.20982906, **H (0K)** = -1202.877682,
H (298K) = --1202.853399, **G (298K)** = -1202.933646 au.
Imaginary frequency = 1.

C	1.3045270	-0.4835140	-0.0196170
N	1.2056230	0.7807160	-0.4735480
O	-1.4081280	0.5552000	-0.0617680
C	-1.1508040	-0.6960250	0.0549830
C	0.1481480	-1.2185210	0.2777080
C	0.2636010	1.5802050	-0.0398040
C	-0.0451610	2.7699750	-0.9418780
O	0.2018990	3.9127440	-0.6174160
O	-0.5372680	2.3890950	-2.1182070
C	-0.7870730	3.4522230	-3.0650200
H	-1.2022470	2.9621090	-3.9443010
H	-1.4990960	4.1680280	-2.6485090
H	0.1462100	3.9634010	-3.3113950
C	0.1132120	1.8407000	1.4680590
O	0.8675070	1.3802030	2.2967320
O	-0.9340140	2.6234570	1.7303230
C	-1.1618150	2.9223320	3.1239670
H	-2.0297470	3.5799130	3.1398570
H	-1.3639440	2.0029090	3.6782670
H	-0.2895320	3.4234040	3.5495320
H	0.2367550	-2.2828050	0.4392970
C	2.6461350	-1.1101040	-0.0533950
C	3.6811230	-0.5057240	-0.7883790
C	2.9213310	-2.2910320	0.6578240
C	4.9506140	-1.0770050	-0.8259460
H	3.4726250	0.4083160	-1.3336800
C	4.1951310	-2.8552180	0.6269310
H	2.1494610	-2.7569680	1.2618460
C	5.2120720	-2.2542430	-0.1193590
H	5.7374200	-0.6035390	-1.4064290
H	4.3947640	-3.7622600	1.1902700
H	6.2034700	-2.6978430	-0.1456880
C	-2.3201400	-1.6084910	-0.0887770
C	-2.1830400	-2.9593590	-0.4524560
C	-3.6081910	-1.0922230	0.1384100
C	-3.3074030	-3.7738530	-0.5753100
H	-1.2027940	-3.3711550	-0.6680770
C	-4.7295790	-1.9091140	0.0182180
H	-3.7134380	-0.0497130	0.4184590
C	-4.5819400	-3.2526960	-0.3377190
H	-3.1888640	-4.8138940	-0.8652580

H	6.4717580	-1.5837418	0.3265122
H	7.4341260	0.5135172	-0.6029968

H	-5.7184010	-1.4999990	0.2050110
H	-5.4564610	-3.8903760	-0.4323190

Fig.2. Molecule **E** (R^1=R^3=Ph,R^2=H, R^4=CO_2Me) (PCM for CH_2Cl_2)

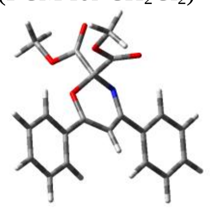

E = -1203.23241389, **H (0K)** = -1202.898200, **H (298K)** = -1202.873976, **G (298K)** = -1202.953821 au.

Imaginary frequency = 0.

C	-1.4628460	0.3755520	0.0462080
N	-1.2768080	-0.8945290	-0.1668530
O	1.0731400	-0.4136400	-0.5381860
C	0.8893630	0.8745840	-0.1344700
C	-0.3489620	1.2870240	0.2453540
C	0.0820390	-1.3407350	-0.0465720
C	0.4167340	-1.6940080	1.4411120
O	-0.3985170	-1.7559950	2.3311030
O	1.7280080	-1.9234890	1.5782770
C	2.1646810	-2.3001910	2.9028510
H	3.2402130	-2.4507850	2.8214110
H	1.9392870	-1.5029850	3.6145570
H	1.6675050	-3.2216970	3.2135830
C	0.2218770	-2.6344290	-0.8773060
O	0.0731200	-3.7353330	-0.3907050
O	0.4563210	-2.3816370	-2.1620990
C	0.5315180	-3.5388490	-3.0256930
H	0.7377370	-3.1429710	-4.0189310
H	1.3358140	-4.2010620	-2.6982080
H	-0.4178580	-4.0784990	-3.0135420
H	-0.5038540	2.2940380	0.6051250
C	-2.8595370	0.8853480	0.0462550
C	-3.1498500	2.2396160	-0.1849100
C	-3.9214660	-0.0063770	0.2761700
C	-4.4700340	2.6911250	-0.1869590
H	-2.3487870	2.9432040	-0.3878670
C	-5.2378020	0.4467170	0.2792570
H	-3.6945870	-1.0508400	0.4605520
C	-5.5166860	1.7975720	0.0476120
H	-4.6793370	3.7403560	-0.3751960
H	-6.0482490	-0.2521140	0.4670340
H	-6.5442580	2.1506370	0.0512540
C	2.1094260	1.6896870	-0.2098290
C	3.3701380	1.0684400	-0.1638980
C	2.0392050	3.0899280	-0.3224290
C	4.5329470	1.8345510	-0.2173850
H	3.4264460	-0.0101130	-0.0689250
C	3.2043040	3.8503270	-0.3717040
H	1.0735420	3.5803870	-0.3948040
C	4.4542580	3.2255020	-0.3194010
H	5.5017510	1.3451170	-0.1751130
H	3.1380220	4.9305690	-0.4630980
H	5.3618590	3.8209180	-0.3625520

Molecule **3a**

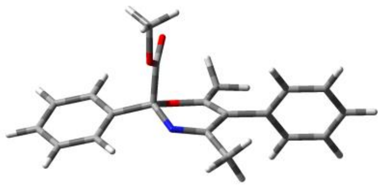

E = -1053,98261270, **H (0K)** = -1053,635630, **H (298K)** = -1053,612746, **G (298K)** = -1053,688512 au.

Imaginary frequency = 0.

C	0.7189370	-0.1118880	1.3054990
N	-0.5664810	-0.0020970	1.3228850
O	-0.5943700	-0.9419850	-0.9011130
C	0.7580990	-0.8659680	-0.9721100
C	1.4823650	-0.3887900	0.0757770
C	-1.2215470	-0.0402400	0.0374270
C	1.4474710	-0.0221450	2.6223890
H	0.7244390	0.1171350	3.4281460
H	2.0375150	-0.9269910	2.8096250
H	2.1531730	0.8167360	2.6194200
C	1.2622400	-1.3884450	-2.2780620
H	2.3515220	-1.4093250	-2.3157590
H	0.8729260	-2.3992860	-2.4490200
H	0.8789730	-0.7527050	-3.0850100
C	2.9718810	-0.2961480	0.0545400
C	3.6047910	0.9566670	0.0110760
C	3.7760680	-1.4462600	0.0998850
C	4.9962480	1.0570530	0.0093070
H	2.9957490	1.8561400	-0.0320870
C	5.1684570	-1.3485690	0.0930480
H	3.3008660	-2.4229080	0.1447630
C	5.7827020	-0.0960220	0.0492630
H	5.4663320	2.0363170	-0.0288210
H	5.7726080	-2.2515140	0.1266350
H	6.8666440	-0.0185520	0.0463250
C	-1.2027980	1.3696510	-0.6376820
O	-0.9658530	1.5606690	-1.8095640
O	-1.5346400	2.3297890	0.2381390
C	-1.6304300	3.6556320	-0.3101900
H	-1.9140080	4.2958140	0.5252760
H	-0.6697270	3.9687710	-0.7274810
H	-2.3886970	3.6893400	-1.0970340
C	-2.6771370	-0.4864880	0.1492530
C	-3.3144710	-0.5436930	1.3910450
C	-3.3913590	-0.8055720	-1.0130970
C	-4.6527230	-0.9330310	1.4709360
H	-2.7499610	-0.2881120	2.2800490
C	-4.7272760	-1.1951210	-0.9283970
H	-2.8958310	-0.7533520	-1.9773230
C	-5.3616460	-1.2604150	0.3144030
H	-5.1408690	-0.9811740	2.4409550
H	-5.2731390	-1.4471350	-1.8338560
H	-6.4034750	-1.5635550	0.3794480

Molecule **3a'**

Molecule **3d**

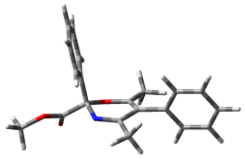

E = --1053,97937122, **H (0K)** = -1053,632594,
H (298K) = -1053,609578, **G (298K)** = -1053,686670 au.
Imaginary frequency = 0.

C	-0.8202842	-1.6423992	-1.0573954
N	0.4631208	-1.7730602	-1.0653004
O	0.4094548	-1.5788912	1.3612936
C	-0.9090862	-1.2793892	1.3124536
C	-1.5837102	-1.2392252	0.1333176
C	1.1715568	-1.4064482	0.1374336
C	-1.5592942	-1.9765052	-2.3291604
H	-0.8470952	-2.3118172	-3.0853554
H	-2.3062872	-2.7599442	-2.1556074
H	-2.1041462	-1.1021852	-2.7037634
C	-1.4646662	-1.0707252	2.6860386
H	-2.5445202	-0.9228892	2.6675736
H	-1.2257352	-1.9368412	3.3143956
H	-0.9895342	-0.1974022	3.1494846
C	-3.0405662	-0.9246152	0.0465786
C	-3.4700522	0.3142118	-0.4561824
C	-4.0138722	-1.8565892	0.4411456
C	-4.8288702	0.6136528	-0.5587654
H	-2.7275912	1.0480588	-0.7596794
C	-5.3738702	-1.5572442	0.3438256
H	-3.6966772	-2.8231932	0.8243546
C	-5.7852042	-0.3214032	-0.1578584
H	-5.1405212	1.5797498	-0.9472784
H	-6.1114312	-2.2917542	0.6567926
H	-6.8437532	-0.0882002	-0.2357374
C	2.3531688	-2.4008582	0.3094746
O	2.4837048	-3.1721542	1.2291046
O	3.2122528	-2.2785042	-0.7142104
C	4.3394198	-3.1681732	-0.6768494
H	4.0060848	-4.2091582	-0.6996474
H	4.9251518	-2.9330952	-1.5657224
H	4.9290648	-3.0022222	0.2287696
C	1.7015858	0.0352098	0.0818546
C	1.8554148	0.7009328	-1.1382294
C	2.0748788	0.6787418	1.2682886
C	2.3605738	2.0013878	-1.1688064
H	1.5806378	0.1929938	-2.0559854
C	2.5786558	1.9791308	1.2348216
H	1.9629878	0.1604968	2.2157046
C	2.7214058	2.6451888	0.0160606
H	2.4721228	2.5119668	-2.1219574
H	2.8598588	2.4715608	2.1621676
H	3.1135658	3.6585368	-0.0097584

Molecule **3e**

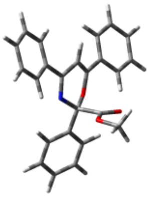

E = -1206,40862646, **H (0K)** = -1206,036033,
H (298K) = -1206,011822, **G (298K)** = -1206,091394 au.
Imaginary frequency = 0.

C	1.4694066	-1.5129673	-0.1282941
N	1.2716856	-0.2333963	-0.2115921
O	-1.0965694	-0.6850213	-0.4800361
C	-0.8870344	-1.9972953	-0.2126521
C	0.3691826	-2.4467473	0.0459049
C	-0.0719484	0.2189667	0.0255129
C	-0.3621904	0.3672147	1.5548869
O	-1.4386064	0.1390487	2.0618329
O	0.7047856	0.8358537	2.2159049
C	0.4983146	1.0830337	3.6184029
H	0.5381156	-3.4838743	0.3000929
H	1.4518956	1.4577597	3.9903459
H	-0.2913654	1.8251667	3.7624669
H	0.2193046	0.1593847	4.1318199
C	-2.1080314	-2.8151103	-0.2748111
C	-3.3442324	-2.2470003	0.0751909
C	-2.0579484	-4.1630773	-0.6667091
C	-4.5034194	-3.0189483	0.0414539
H	-3.3730754	-1.2133893	0.4005729
C	-3.2195994	-4.9300653	-0.6986351
H	-1.1108864	-4.5986433	-0.9711641
C	-4.4453394	-4.3597583	-0.3449501
H	-5.4540854	-2.5743703	0.3228999
H	-3.1714614	-5.9696463	-1.0109781
H	-5.3519234	-4.9583253	-0.3738451
C	2.8669666	-2.0097573	-0.2491071
C	3.9346576	-1.1376113	0.0203589
C	3.1525356	-3.3301353	-0.6281081
C	5.2510506	-1.5774813	-0.0824391
H	3.7061766	-0.1196543	0.3171879
C	4.4723676	-3.7685763	-0.7362651
H	2.3435856	-4.0143163	-0.8649251
C	5.5249586	-2.8950613	-0.4610271
H	6.0670596	-0.8937923	0.1365119
H	4.6771036	-4.7918953	-1.0393121
H	6.5532226	-3.2379533	-0.5403881
C	-0.3441414	1.5686267	-0.6372081
C	-1.6368164	2.1084727	-0.6032751
C	0.6840846	2.2793947	-1.2614411
C	-1.8976314	3.3405877	-1.2006891
H	-2.4343774	1.5618857	-0.1107171
C	0.4190336	3.5136127	-1.8575801
H	1.6797536	1.8524577	-1.2765561
C	-0.8699044	4.0469677	-1.8299511
H	-2.9042424	3.7495107	-1.1738451
H	1.2236326	4.0577367	-2.3454401
H	-1.0737444	5.0083617	-2.2943321

Molecule **3o**

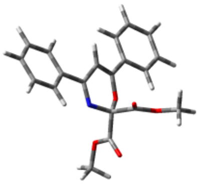

E = -1203,21905801, **H (0K)** = -1202,884711,
H (298K) = -1202,860486, **G (298K)** = -1202,940349 au.
Imaginary frequency = 0.

C	-1.3884823	0.6270172	0.1872647
N	-1.2152943	-0.6424948	-0.0168553
O	1.1433867	-0.1904278	-0.3893713
C	0.9692987	1.1000312	0.0013077
C	-0.2639743	1.5321172	0.3717197
C	0.1350467	-1.1094748	0.0943207
C	0.4616617	-1.4807718	1.5768457
O	-0.3511133	-1.5457678	2.4642287
O	1.7784957	-1.7164788	1.7181317
C	2.1825207	-2.1383658	3.0332007
H	3.2602867	-2.2903888	2.9726827
H	1.9411897	-1.3696688	3.7718097
H	1.6753647	-3.0681318	3.3025827
C	0.2677257	-2.3911318	-0.7555653
O	0.2258267	-3.5038288	-0.2826183
O	0.3776327	-2.1087158	-2.0568273
C	0.4317977	-3.2532428	-2.9278313
H	0.5268327	-2.8464918	-3.9344773
H	1.2923947	-3.8800328	-2.6804763
H	-0.4822223	-3.8450178	-2.8337063
H	-0.4076463	2.5420772	0.7287307
C	-2.7819363	1.1448122	0.1983617
C	-3.0720643	2.4962632	-0.0423333
C	-3.8410343	0.2575862	0.4518837
C	-4.3903713	2.9516532	-0.0302953
H	-2.2704843	3.1937222	-0.2657363
C	-5.1552683	0.7146922	0.4691967
H	-3.6071293	-0.7842468	0.6419097
C	-5.4347093	2.0632202	0.2285817
H	-4.6006343	3.9995562	-0.2266873
H	-5.9648743	0.0197912	0.6756057
H	-6.4615793	2.4189462	0.2438497
C	2.2014657	1.9000062	-0.0689983
C	3.4519537	1.2618602	-0.0060943
C	2.1540837	3.2990342	-0.1942373
C	4.6256967	2.0109062	-0.0544863
H	3.4870997	0.1830542	0.0961077
C	3.3295767	4.0429202	-0.2377543
H	1.1951407	3.8005822	-0.2819043
C	4.5692867	3.4013532	-0.1680323
H	5.5871997	1.5077282	-0.0000303
H	3.2796657	5.1234972	-0.3392083
H	5.4860987	3.9832852	-0.2075343

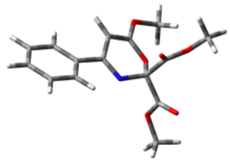

E = -1086,68912358, **H (0K)** = -1086,403125,
H (298K) = -1086,380868, **G (298K)** = -1086,455620 au.
Imaginary frequency = 0.

C	-3.1776497	1.3156335	-0.1773996
C	-2.2442387	0.4014265	0.3336684
C	-2.7093207	-0.7712255	0.9509494
C	-4.0748157	-1.0178975	1.0588114
C	-4.9977227	-0.1009225	0.5474524
C	-4.5456237	1.0643395	-0.0732186
C	-0.7794847	0.6378815	0.2370134
N	0.0038033	-0.4006955	0.3108704
O	1.8323043	1.0142405	-0.4603556
C	1.0234883	2.0800835	-0.3726896
C	-0.2675237	1.9772245	0.0443784
C	1.3972083	-0.1189595	0.3645624
C	1.8570983	0.1454555	1.8360274
O	1.2059673	-0.1241615	2.8124174
O	3.0851693	0.6993675	1.8579994
C	3.6365233	0.8907955	3.1741044
H	-2.8375607	2.2155315	-0.6807486
H	-1.9799187	-1.4693785	1.3468694
H	-4.4220347	-1.9247935	1.5466914
H	-6.0638877	-0.2941165	0.6331974
H	-5.2572777	1.7776145	-0.4803706
H	-0.8835067	2.8619245	0.1071374
H	4.6236603	1.3260365	3.0169244
H	3.0047303	1.5643885	3.7587574
H	3.7151623	-0.0680885	3.6921734
C	2.9743543	3.2718965	-1.0369926
H	3.1992363	4.3273295	-1.1925006
H	3.5308803	2.8804045	-0.1821246
H	3.2271473	2.6976845	-1.9321946
O	1.5570083	3.2281355	-0.7872966
C	2.1731463	-1.3425405	-0.1653786
O	2.7363223	-2.1320495	0.5576074
O	2.0955543	-1.4330915	-1.4964106
C	2.7241313	-2.5956105	-2.0671686
H	2.5671653	-2.5109705	-3.1423626
H	3.7912833	-2.6072535	-1.8308726
H	2.2603903	-3.5058055	-1.6789236

Molecule **3n**

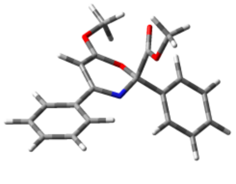

E = -1089,88073946, **H (0K)** = -1089,556222,

Molecule **4a**

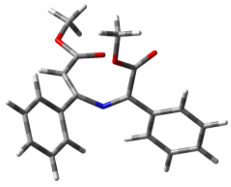

E = -1089,89396454, **H (0K)** = -1089,570867,

H (298K) = -1089,534070, **G (298K)** = -1089,608062 au.
Imaginary frequency = 0.

C	-1.0947120	0.4236283	0.0825045
N	-0.1121210	-0.4296197	0.1507115
O	1.3978500	1.3837443	-0.4281655
C	0.3851250	2.2512893	-0.3123855
C	-0.8752020	1.8503503	0.0128595
C	1.1931610	0.1300663	0.3185395
C	1.4744890	0.5067433	1.8110325
O	2.0729900	1.5016453	2.1613125
O	1.0233090	-0.4391407	2.6430255
C	1.3041500	-0.2182267	4.0370055
H	0.8814610	-1.0775607	4.5573655
H	2.3828670	-0.1574157	4.2034625
H	0.8358700	0.7085013	4.3781655
C	2.2978940	-0.8081737	-0.1582215
C	1.9953000	-2.0994447	-0.5966175
C	3.6306030	-0.3753547	-0.1436995
C	3.0169430	-2.9469837	-1.0286095
H	0.9602100	-2.4199287	-0.5961635
C	4.6477850	-1.2242257	-0.5770455
H	3.8666170	0.6251603	0.2054035
C	4.3431690	-2.5130737	-1.0212365
H	2.7736980	-3.9490187	-1.3722865
H	5.6784890	-0.8796067	-0.5664355
H	5.1366180	-3.1754837	-1.3575095
O	0.6809050	3.5132473	-0.6117545
C	2.0715850	3.8949473	-0.6278385
H	2.0622660	4.9831843	-0.6959195
H	2.5645090	3.5674673	0.2901645
H	2.5789860	3.4685223	-1.4969225
H	-1.6674240	2.5792543	0.0987275
C	-2.4788290	-0.1230947	0.0560025
C	-3.5516590	0.6075943	-0.4764005
C	-2.7240260	-1.4056987	0.5730055
C	-4.8386310	0.0698243	-0.4920005
H	-3.3785470	1.5904953	-0.9037035
C	-4.0095260	-1.9394617	0.5617845
H	-1.8898510	-1.9628977	0.9855575
C	-5.0719660	-1.2033287	0.0294025
H	-5.6577660	0.6448773	-0.9154895
H	-4.1861190	-2.9305077	0.9717175
H	-6.0755300	-1.6206347	0.0213445

H (298K) = -1089,547598, **G (298K)** = -1089,625835 au.
Imaginary frequency = 0.

C	-1.7340550	0.0936145	-0.6715220
N	-0.4122790	-0.3071565	-0.7126390
O	-0.1323340	2.6308165	-0.7793140
C	-1.3188490	2.5378375	-1.0549200
C	-2.1716880	1.3587005	-0.9323770
C	0.5626040	-0.0918875	0.0821380
C	0.4143930	0.6842235	1.3890430
O	1.2915800	1.3470615	1.8944760
O	-0.8074400	0.5166825	1.9210140
C	-1.0593330	1.2629655	3.1243540
H	-2.0761150	1.0038815	3.4189650
H	-0.3459990	0.9816295	3.9032860
H	-0.9722260	2.3346915	2.9292020
C	1.9103030	-0.6040455	-0.2755370
C	2.1602760	-0.9762095	-1.6086210
C	2.9301390	-0.7697095	0.6762950
C	3.3939890	-1.4985595	-1.9797350
H	1.3738620	-0.8396715	-2.3430710
C	4.1629870	-1.3030035	0.3014030
H	2.7666630	-0.4765395	1.7055540
C	4.4006320	-1.6665375	-1.0239330
H	3.5743830	-1.7716515	-3.0159020
H	4.9406850	-1.4290355	1.0495800
H	5.3654720	-2.0744535	-1.3135340
O	-2.0397930	3.5949545	-1.5244990
C	-1.3045310	4.8161075	-1.6655230
H	-2.0250160	5.5526195	-2.0243560
H	-0.8809220	5.1291365	-0.7067490
H	-0.4889580	4.6978665	-2.3847860
H	-3.2257140	1.5109915	-1.1288660
C	-2.6970930	-1.0324965	-0.5303160
C	-3.9699310	-0.8214845	0.0250440
C	-2.3440120	-2.3310015	-0.9335910
C	-4.8721050	-1.8753195	0.1525680
H	-4.2416120	0.1677225	0.3801830
C	-3.2491220	-3.3828825	-0.8059760
H	-1.3611850	-2.5018125	-1.3592910
C	-4.5167660	-3.1598055	-0.2652880
H	-5.8508370	-1.6949405	0.5891070
H	-2.9636700	-4.3792925	-1.1326220
H	-5.2206500	-3.9814785	-0.1638230

Molecule **4b**

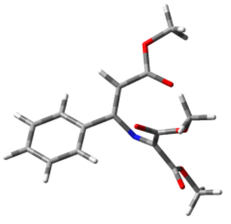

E = -1086,70133287, **H (0K)** = -1086,416656,
H (298K) = -1086,393313, **G (298K)** = -1086,471359 au.
Imaginary frequency = 0.

C	-2.8190268	-0.0090898	-0.6442460
C	-1.6989988	-0.6684218	-0.1144850
C	-1.8883358	-1.8535848	0.6124320
C	-3.1714438	-2.3560298	0.8198850
C	-4.2809848	-1.6915188	0.2938340

Molecule **4g**

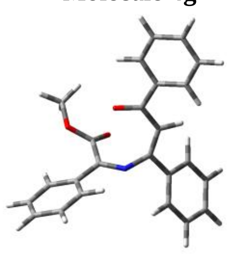

E = -1206,40306201, **H (0K)** = -1206,031770,
H (298K) = -1206,006664, **G (298K)** = -1206,088934 au.
Imaginary frequency = 0.

C	-0.1866517	1.3698218	-0.3139387
N	-1.2791297	0.6040838	-0.6798377
O	0.7307143	-1.4206902	0.0630173
C	1.5063523	-0.4976682	-0.2169677

C	-4.1005108	-0.5174758	-0.4398240
C	-0.3261978	-0.1248358	-0.3099730
N	0.6115442	-1.0812028	-0.7008890
O	2.3199802	1.2976902	-0.4474890
C	1.2268092	1.8410012	-0.4662110
C	-0.0840148	1.2106092	-0.2904470
C	1.5997612	-1.5131588	-0.0363190
C	1.9065242	-1.1019648	1.3949590
O	1.0461172	-0.8791728	2.2180150
O	3.2212622	-1.0045098	1.5975010
C	3.6159992	-0.5665208	2.9105110
H	-2.6814348	0.8897372	-1.2375890
H	-1.0312428	-2.3595548	1.0437100
H	-3.3041698	-3.2673098	1.3965560
H	-5.2803178	-2.0881248	0.4514850
H	-4.9575558	-0.0002158	-0.8625470
H	-0.9195328	1.8820172	-0.1348420
H	4.7051802	-0.5482148	2.8935470
H	3.2143652	0.4298832	3.1105300
H	3.2509912	-1.2647158	3.6678010
C	2.3045932	3.9150572	-0.7804070
H	2.0097892	4.9600702	-0.8845280
H	2.9433772	3.7825952	0.0976290
H	2.8527372	3.5820072	-1.6663070
O	1.0816702	3.1827152	-0.6344590
C	2.4664252	-2.6032228	-0.6266040
O	2.9251972	-3.4949728	0.0555840
O	2.6187882	-2.4828168	-1.9480770
C	3.3871182	-3.5279788	-2.5719980
H	3.4234052	-3.2670208	-3.6293320
H	4.3933352	-3.5657168	-2.1467520
H	2.9002722	-4.4960438	-2.4283790

C	1.0982053	0.9057188	-0.2344797
C	-1.9069507	-0.2829432	-0.0121357
C	-1.5130087	-0.5511912	1.4383513
O	-1.0784047	0.3074718	2.1727053
O	-1.6982887	-1.8326512	1.7855703
C	-1.1808457	-2.1944562	3.0781863
H	1.8801133	1.6545048	-0.2033487
H	-1.4192897	-3.2509512	3.2004423
H	-0.0995817	-2.0379982	3.0968133
H	-1.6491967	-1.5958482	3.8636063
C	2.9443783	-0.8224542	-0.5224117
C	3.4168613	-2.0967312	-0.1709647
C	3.8176783	0.0704378	-1.1620447
C	4.7333843	-2.4632832	-0.4339957
H	2.7265403	-2.7838682	0.3071183
C	5.1352223	-0.2992532	-1.4335407
H	3.4660373	1.0485368	-1.4748697
C	5.5975383	-1.5636622	-1.0651777
H	5.0886483	-3.4504122	-0.1498767
H	5.7989213	0.3990108	-1.9364987
H	6.6255323	-1.8492952	-1.2728117
C	-0.4769867	2.8263458	-0.2449587
C	-1.5167217	3.3887378	-1.0040757
C	0.2742153	3.6614758	0.5983573
C	-1.7795297	4.7556698	-0.9391197
H	-2.1036257	2.7464208	-1.6518687
C	0.0066583	5.0270108	0.6630573
H	1.0452623	3.2289898	1.2281783
C	-1.0181207	5.5800818	-0.1083607
H	-2.5799717	5.1782238	-1.5406457
H	0.5906883	5.6582928	1.3273653
H	-1.2267717	6.6453248	-0.0557537
C	-3.0288617	-1.0123442	-0.6534057
C	-4.0785957	-1.5706342	0.0930853
C	-3.0707567	-1.1008692	-2.0555137
C	-5.1463777	-2.1959612	-0.5493907
H	-4.0650977	-1.5160692	1.1755413
C	-4.1304917	-1.7375332	-2.6929107
H	-2.2569077	-0.6701192	-2.6290947
C	-5.1742447	-2.2853242	-1.9414097
H	-5.9560857	-2.6161822	0.0407713
H	-4.1428997	-1.8098492	-3.7770427
H	-6.0028767	-2.7816482	-2.4395017

Molecule **4h**

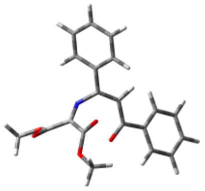

E = -1203,21156823, **H (0K)** = -1202,878683,
H (298K) = -1202,853536, **G (298K)** = -1202,936080 au.
Imaginary frequency = 0.

C	-0.1882104	0.2273438	0.0382553
N	-0.9266134	-0.8413962	-0.4577297
O	1.6145196	-2.1108862	0.3229613
C	2.0374956	-0.9617822	0.1516633
C	1.1684286	0.2180128	0.1915683
C	-1.2098604	-1.9268192	0.1306333
C	-2.0906344	-2.9315672	-0.5789377
O	-2.9781004	-3.5278522	-0.0086167

O	-1.7977314	-3.0396342	-1.8799427	
C	-2.6365794	-3.9455522	-2.6200217	
H	-2.2517374	-3.9278322	-3.6393947	
H	-2.5733264	-4.9522912	-2.1991007	
H	-3.6767524	-3.6106152	-2.5925497	
C	-0.8403324	-2.2238962	1.5759663	
O	-0.7479704	-1.3654122	2.4234563	
O	-0.6430234	-3.5309602	1.7615013	
C	-0.1951994	-3.9042482	3.0760903	
H	-0.0994934	-4.9894592	3.0524603	
H	0.7688756	-3.4345062	3.2866783	
H	-0.9246574	-3.5954142	3.8288893	
H	1.6414376	1.1831628	0.3263503	
C	-0.9725814	1.4818088	0.1598683	
C	-2.0765874	1.7249068	-0.6743637	
C	-0.6263454	2.4397108	1.1269893	
C	-2.7988304	2.9108938	-0.5591657	
H	-2.3490524	0.9858608	-1.4202397	
C	-1.3533824	3.6223228	1.2406943	
H	0.1930156	2.2366998	1.8094573	
C	-2.4391514	3.8640518	0.3957463	
H	-3.6435484	3.0917688	-1.2183707	
H	-1.0799794	4.3505228	1.9994013	
H	-3.0065104	4.7863178	0.4872133	
C	3.5086216	-0.7496672	-0.0762667	
C	4.0436076	0.4366308	-0.6015237	
C	4.3778836	-1.8090972	0.2286533	
C	5.4175136	0.5629488	-0.8073337	
H	3.3884466	1.2569118	-0.8774617	
C	5.7493316	-1.6802352	0.0316623	
H	3.9497986	-2.7264252	0.6190793	
C	6.2732656	-0.4918752	-0.4859897	
H	5.8185596	1.4836768	-1.2224027	
H	6.4125556	-2.5049012	0.2790553	
H	7.3441236	-0.3906522	-0.6420297	

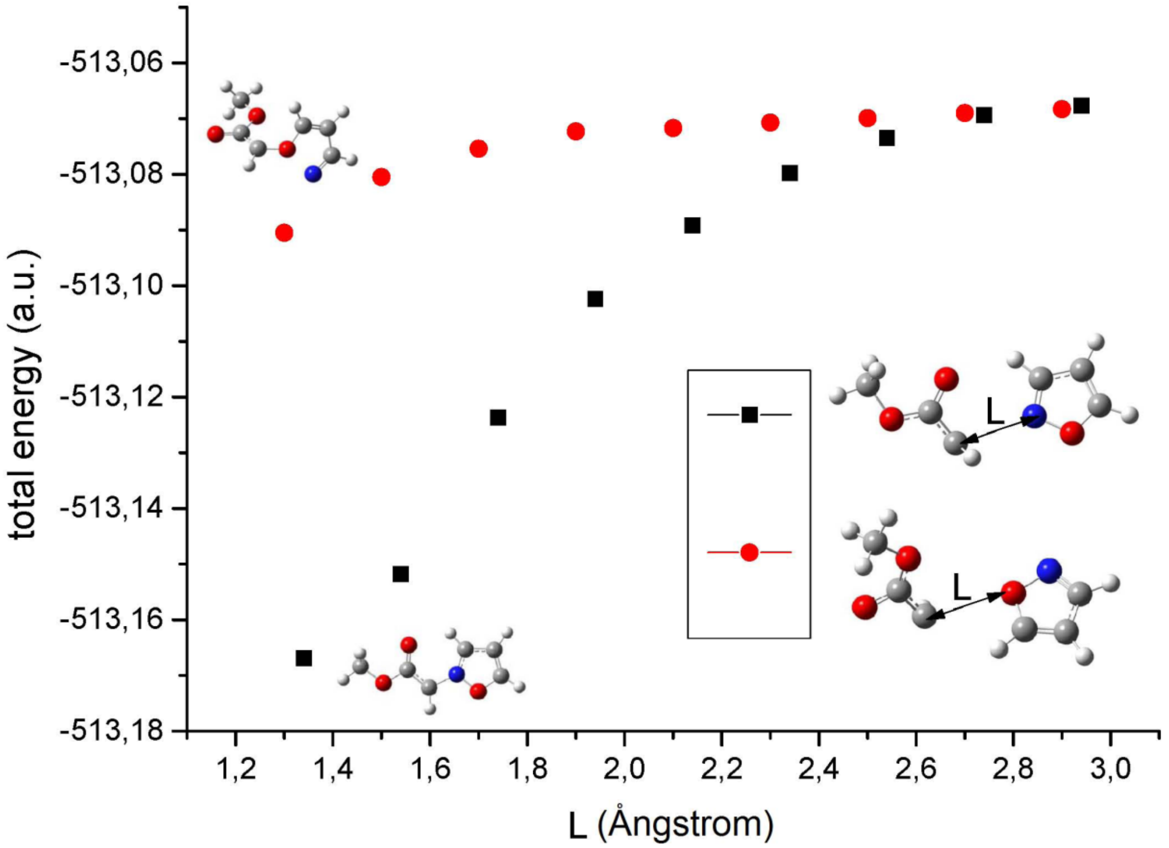

Energy profile of the methoxycarbonylcarbene attack on the nitrogen and the oxygen of the isoxazole calculated at the DFT/B3LYP/6-31G (d) level.
